# Adverse events following first and second dose COVID-19 vaccination in England, October 2020 to September 2021: a national vaccine surveillance platform self-controlled case series study

**DOI:** 10.2807/1560-7917.ES.2023.28.3.2200195

**Published:** 2023-01-19

**Authors:** Ruby SM Tsang, Mark Joy, Rachel Byford, Chris Robertson, Sneha N Anand, William Hinton, Nikhil Mayor, Debasish Kar, John Williams, William Victor, Ashley Akbari, Declan T Bradley, Siobhan Murphy, Dermot O’Reilly, Rhiannon K Owen, Antony Chuter, Jillian Beggs, Gary Howsam, Aziz Sheikh, FD Richard Hobbs, Simon de Lusignan

**Affiliations:** 1 Nuffield Department of Primary Care Health Sciences, University of Oxford, Oxford, United Kingdom; 2 Department of Mathematics and Statistics, University of Strathclyde, Glasgow, United Kingdom; 3 Public Health Scotland, Glasgow, United Kingdom; 4 Chelsea and Westminster Hospital NHS Foundation Trust, London, United Kingdom; 5 Royal College of General Practitioners, London, United Kingdom; 6 Population Data Science, Swansea University Medical School, Swansea University, United Kingdom; 7 Centre for Public Health, Queen’s University Belfast, Belfast, United Kingdom; 8 Public Health Agency, Belfast, United Kingdom; 9 BREATHE – The Health Data Research Hub for Respiratory Health, Edinburgh, United Kingdom; 10 Usher Institute, University of Edinburgh, Edinburgh, United Kingdom

**Keywords:** Sentinel surveillance, Medical record systems, computerised, COVID-19, COVID-19 Vaccines, Drug-Related Side Effects and Adverse Reactions, SNOMED CT, Primary care, Vaccination

## Abstract

**Background:**

Post-authorisation vaccine safety surveillance is well established for reporting common adverse events of interest (AEIs) following influenza vaccines, but not for COVID-19 vaccines.

**Aim:**

To estimate the incidence of AEIs presenting to primary care following COVID-19 vaccination in England, and report safety profile differences between vaccine brands.

**Methods:**

We used a self-controlled case series design to estimate relative incidence (RI) of AEIs reported to the national sentinel network, the Oxford-Royal College of General Practitioners Clinical Informatics Digital Hub. We compared AEIs (overall and by clinical category) 7 days pre- and post-vaccination to background levels between 1 October 2020 and 12 September 2021.

**Results:**

Within 7,952,861 records, 781,200 individuals (9.82%) presented to general practice with 1,482,273 AEIs, 4.85% within 7 days post-vaccination. Overall, medically attended AEIs decreased post-vaccination against background levels. There was a 3–7% decrease in incidence within 7 days after both doses of Comirnaty (RI: 0.93; 95% CI: 0.91–0.94 and RI: 0.96; 95% CI: 0.94–0.98, respectively) and Vaxzevria (RI: 0.97; 95% CI: 0.95–0.98). A 20% increase was observed after one dose of Spikevax (RI: 1.20; 95% CI: 1.00–1.44). Fewer AEIs were reported as age increased. Types of AEIs, e.g. increased neurological and psychiatric conditions, varied between brands following two doses of Comirnaty (RI: 1.41; 95% CI: 1.28–1.56) and Vaxzevria (RI: 1.07; 95% CI: 0.97–1.78).

**Conclusion:**

COVID-19 vaccines are associated with a small decrease in medically attended AEI incidence. Sentinel networks could routinely report common AEI rates, contributing to reporting vaccine safety.

**Key public health message**

**What did you want to address in this study?**

We wanted to compare how frequently a selected list of adverse events occurred in the 7 days after people received their first and second doses of a COVID-19 vaccine compared to background levels, using real-world data from general practices in England. We also examined differences in safety profiles between the vaccine brands.

**What have we learnt from this study?**

We found that the rates of general practitioner consultations for these adverse events decreased by 3–7% after two doses of Comirnaty or Vaxzevria in the 7 days after vaccination, but increased by 20% after the first dose of Spikevax. The specific types of adverse events reported differed slightly by vaccine brand.

**What are the implications of your findings for public health?**

The rates of adverse events following COVID-19 vaccination appear to be generally low across the three vaccines used in the United Kingdom. Using computerised medical records to study patterns of vaccine adverse events will be important in the future as COVID-19 becomes endemic and ongoing vaccination is required.

## Introduction

The coronavirus disease (COVID-19) immunisation programme in the United Kingdom (UK) began in December 2020, with the UK’s Joint Committee on Vaccination and Immunisation (JCVI) initially recommending COVID-19 vaccination for all adults aged 18 years and over, and prioritising older adults, care home residents and staff, health and social care workers and individuals in clinical risk groups. This was later expanded to include children and young people aged 12 years and over with underlying chronic conditions that put them at risk of serious COVID-19 in July 2021, all 16 to 17-year-olds in August 2021, and all 12 to 15-year-olds (first dose only) in September 2021. The COVID-19 booster programme, of third and later further vaccinations, also commenced in September 2021. The vaccines currently being used until December 2022 in the UK are Comirnaty (BNT162b2 mRNA, BioNTech-Pfizer), Vaxzevria (ChAdOx1-S, Oxford-AstraZeneca) and Spikevax (mRNA-1273, Moderna). Studies have shown that these vaccines are highly effective at reducing severe COVID-19 [1-4].

The safety of COVID-19 vaccines was rigorously assessed through clinical trials before they received emergency use authorisation, and these trials showed that serious adverse events were rare [5-7]. However, to detect rarer adverse events of interest (AEIs) following immunisations, post-licensure follow-up is needed in larger general populations. Examples include the extremely rare adverse event of concurrent thrombosis and thrombocytopenia (‘thrombotic thrombocytopenia syndrome’ (TTS)) that has been reported following vaccination with the first dose of Vaxzevria, and myocarditis and acute pericarditis reported after Comirnaty or Spikevax vaccination. The former was only detected as national immunisation programmes rolled out worldwide, which led the JCVI to advise that adults aged under 40 years of age should be offered an alternative in May 2021 [8]. A summary of adverse events associated with COVID-19 vaccines that were detected post-licensure is presented in the **Box** below.

Box **Summary of COVID-19 vaccine safety signals detected in post-licensure surveillance from October 2020–September 2021**

**Thrombotic thrombocytopenia syndrome**

Thrombotic thrombocytopenia syndrome (TTS), also known as vaccine-induced immune thrombosis and thrombocytopenia (VITT), is a very rare immune condition, in which pathologic antibodies to platelet factor 4 cause blood clots in different parts of the body as well as a low platelet count. A disproportionate number of cases of these rare events have been reported after the first dose of Vaxzevria vaccination [38,50], with the signal later being confirmed in population studies [15,39]. During the investigations, a number of countries suspended the use of Vaxzevria, and later restricted their use to certain age groups.

**Myocarditis and pericarditis**

Cases of myocarditis and pericarditis have been reported following Comirnaty and Spikevax vaccination [39,51]. Large observational studies have since been conducted across different countries, which observed a short-term increase in risk of myocarditis and pericarditis, particularly in younger individuals. The evidence is mixed with regards to whether males or females are at higher risk of experiencing these adverse events [32-34].

**Neurological complications**

A number of cases of rare neurological adverse events such as Guillain–Barré syndrome (GBS) and Bell’s palsy have been reported since large-scale vaccination programmes have commenced around the world. Increased risks of GBS and Bell’s palsy after Vaxzevria vaccination were identified in an English cohort, with the association between Vaxzevria and GBS replicated in an independent Scottish cohort [35]. Subsequent studies describe rare and generally minor neurological events following vaccination [36,37].

Post authorisation surveillance is required to continually assess vaccine safety in the real world and to maintain public confidence in vaccines, including for COVID-19 [9]. While such surveillance platforms are well established in influenza vaccination [10,11], often using computerised medical record (CMR) data [12], no equivalent systems have been established for COVID-19 vaccination in the UK beyond the generic adverse events reporting systems. This study was conducted to estimate the incidence of a list of prespecified AEIs presenting to general practice following first and second doses of a COVID-19 vaccination compared with background levels using ‘real-world’ primary care data, and to explore differences in safety profiles between vaccine brands. 

## Methods

### Data source

**Figure 1:**
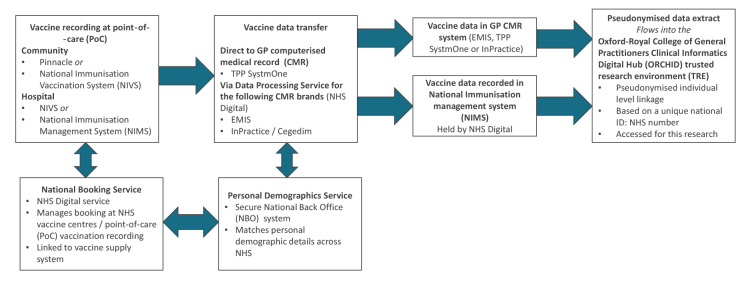
Flow of data from point-of-care vaccination through to the Oxford-Royal College of General Practitioners Clinical Informatics Digital Hub (ORCHID) in England

CMR: computerised medical record; GP: general practitioner; NBS: National Back Office; NHS: National Health Service; NIMS: National Immunisation Management System; NIVS: National Immunisation Vaccination System; POC: point-of-care.

We used data from the Oxford-Royal College of General Practitioners Clinical Informatics Digital Hub (ORCHID), England [13], which were derived from pseudonymised extracts of computerised primary care records. Such a sentinel surveillance database was established in 1957 and has been used for influenza monitoring and assessing influenza vaccine effectiveness since 1967 in influenza vaccine safety surveillance [14]. The UK has registration-based primary care, where one patient registers with a single general practice, and CMRs have been in routine use for over 20 years. At the time of this study, the sentinel network cohort included around 8 million (n = 7,952,861) patient records from general practices across England. COVID-19 vaccine data, including vaccine date, type, and dose of all individuals vaccinated in England, are automatically transferred into the general practice CMR directly or via NHS Digital’s Data Processing Service (DPS) (**Figure 1**). In addition, the ORCHID receives direct feed from the National Immunisation Management System (NIMS), and while there were differences between data sources at the start of vaccination December 2019 to March 2020, the direct DPS transfer route is reliable.

PoC vaccination can be given at the hospital or community. Vaccination bookings and vaccination supplies are managed through a National Booking Service. A Personal Demographic Service (PDS) securely holds all individuals’ demographic data to ensure matching across the English NHS, data are linked using the NHS number, which is pseudonymised before sending into the ORCHID trusted research environment (TRE). Pinnacle is a system where the vaccination information was first stored and was later available via NIMS.

### Prespecified adverse events of interest

Using the pseudonymised data, patients were retrospectively followed up for consultations (attendance in primary care) for prespecified AEIs that were determined based on adverse events reported in clinical trials and post-licensure surveillance (see **Table 1** for the included conditions). This list was developed through mapping potential adverse events listed in the regulatory approval documents published by the Medicines and Healthcare products Regulatory Agency (MHRA) and the European Medicines Agency (EMA) to Systematized Nomenclature of Medicine Clinical Terms (SNOMED CT). The SNOMED CT concept IDs used within the study are shown in Supplementary Table S1. Clinical consultations for adverse events are recorded into primary care CMR systems using SNOMED CT, and then curated into variables for research studies. We have excluded thrombotic and haemorrhagic events from this analysis as they have already been investigated in a separate study [15,16].

**Table 1:** List of adverse events of interest following COVID-19 vaccination included in this study, England, 1 October 2020–12 September 2021

Category	Adverse events of interest
General non-specific	Asthenia, fatigue, fever, fever with chills, malaise, oedema of face
Injection site	Bruising, burning, erythema, induration, inflammation, irritation, pain, pruritus, rash, swelling, urticaria
Ear	Tinnitus
Gastrointestinal	Abdominal pain, diarrhoea, nausea, vomiting
Immune	Anaphylaxis, hypersensitivity reaction
Lymphatic	Lymphadenopathy
Metabolic/nutrition	Decrease in appetite
Musculoskeletal	Joint pain, myalgia, weakness
Neurological	Bell’s palsy, dizziness, drowsiness, Guillain–Barré syndrome, headache, lethargy, paraesthesia, peripheral tremor
Psychiatric	Insomnia
Respiratory	Cough, influenza-like illness, sneezing, sore throat
Skin	Angioedema, eruption of skin, hyperhidrosis
Vascular	Capillary leak syndrome, myocarditis, pericarditis

### Data extraction

We extracted the following data: date of birth, sex, self-reported ethnicity using an ontology to maximise data capture [17], socioeconomic status using the 2019 English Index of Multiple Deprivation (IMD) quintile [18], date of death, dates of registration and deregistration at the general practice, COVID-19 vaccination dates (first and second dose), COVID-19 vaccine brand (first and second dose), AEI date and AEI type. IMD quintile was derived using the postcode of the patient at the individual level at the point of data extraction, after which the postcode is not retained. Where the IMD quintile for the patient was missing, this was imputed using the postcode of the general practice at which they were registered.

### Inclusion/exclusion criteria

We included all patients aged 16 and over on the study index date (1 October 2020; n = 1,819,782) who reported at least one consultation for any of the listed AEIs between the study index date and the latest data extract date (12 September 2021). We described these attendances as ‘medically attended events*’* or ‘medically attended AEIs*’.* The age cut-off of 16 years was selected based on vaccination guidelines at the time of the study.

**Figure 2:**
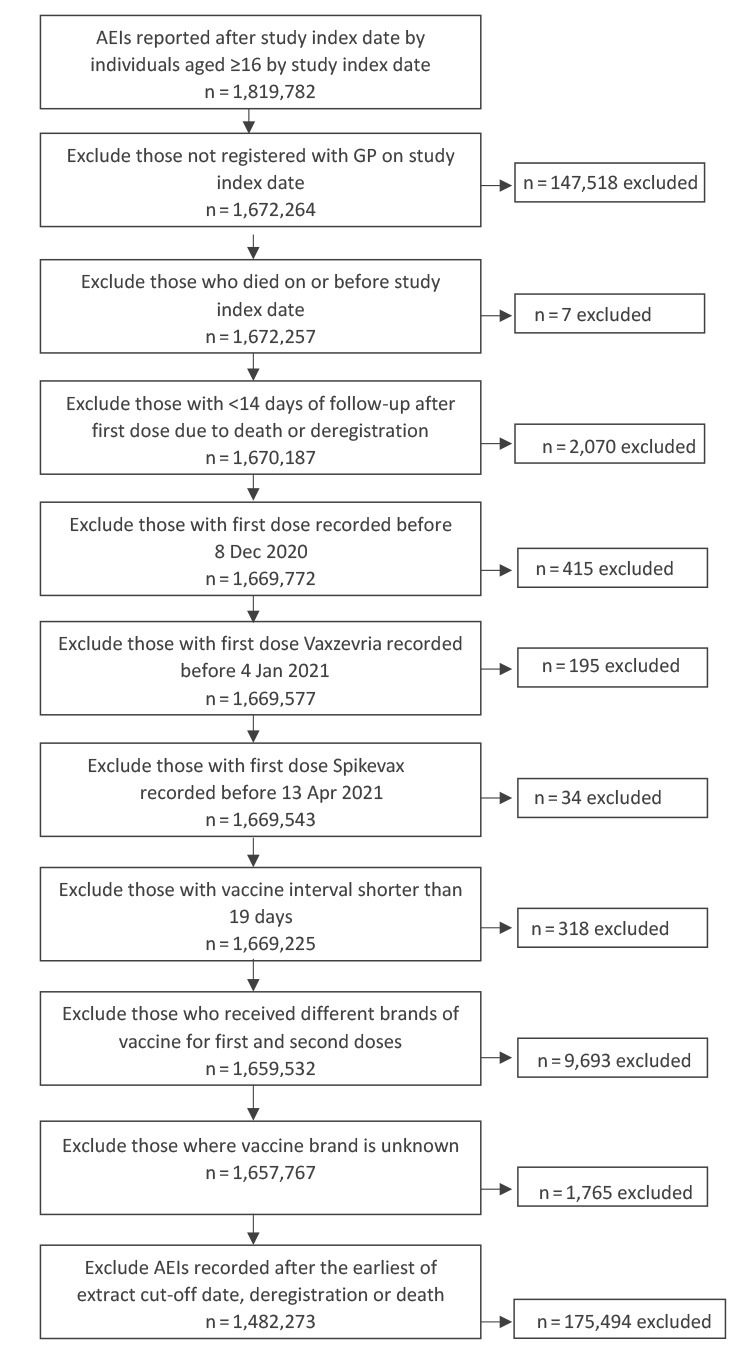
Flow diagram describing the sample selection of individuals reporting adverse events of interest after COVID-19 vaccination, England, 1 October 2020–12 September 2021

AEI: adverse events of interest; COVID-19: coronavirus disease; GP: general practitioner.

Patients were excluded if they: (i) were not registered with a general practice on 1 October 2020, (ii) died on or before 1 October 2020, (iii) had less than 14 days of follow-up after their first dose vaccination because of deregistration or death, (iv) their first dose of any COVID-19 vaccine recorded before 8 December 2020, (v) their first dose Vaxzevria vaccine recorded before 4 January 2021, (vi) their first dose Spikevax vaccine recorded before 13 April 2021, (vii) received their second dose less than 19 days following their first dose, (viii) received different brands of vaccines for their first and second dose, (ix) did not have a vaccine brand recorded or (x) had medically attended AEIs recorded after the extract cut-off date or were censored (deregistration or death, which may suggest error in their records). Inclusion/exclusion at each step is shown in the flow diagram in **Figure 2**. 

Medically attended AEIs recorded after the earliest of extract up-to date, deregistration date and date of death for the individual were also excluded. UK primary care records include a deregistration date and date of death, so this enabled us to only include data for the period where the vaccinated person was at risk of experiencing a medically attended AEI. We excluded any COVID-19 vaccines administered before 8 December 2020 as this was the first date of the licenced use of Comirnaty in the UK, with Vaxzevria and Spikevax being available for licenced administration from 4 January and 13 April 2021, respectively. Some vaccination from clinical trials and overseas administration is recorded before these dates in primary care CMR systems.

Although excluded from further analysis, we also report the characteristics of the unvaccinated population, which are individuals without a vaccination record as of data extraction date. The individuals included in this group were all over 16 years of age.

### Self-controlled case series design

A self-controlled case series (SCCS) design was used to analyse the rates of adverse events of interest post vaccination [19,20]. The SCCS method is a case-only method, in which the rate of events during pre-defined risk periods are compared with the rate of events during the rest of the observation period, i.e. control period. The incidence of events within this control period is considered to be a reflection of background levels of such events unrelated to the intervention. Each individual is their own control in such an analysis, and potential time-invariant confounding effects of between-person characteristics are thus eliminated. This method is particularly useful for evaluating vaccine safety, as it is often difficult to identify a comparator group since most in the population will receive a vaccine, and those who may not be suitable comparators, i.e. they were not vaccinated for medical reasons.

We conducted separate SCCS models for the three brands of vaccines. Individuals meeting the aforementioned inclusion/exclusion criteria and received one or two doses of Comirnaty, Vaxzevria or Spikevax were included in the respective models. Those with no record of having had any COVID-19 vaccines were classified as unvaccinated. The observation period began on the study index date of 1 October 2020, and ended on the earliest of the patient’s death, deregistration from their general practice, or data extract end date. We only included vaccinated individuals in the SCCS.

### Model description

For all models, we defined pre-exposure and risk periods relative to the day of vaccination (day 0), with days −7 to −1 as the pre-exposure period and days 0 to 7 as the risk period for both doses. The time outside of these defined periods is used as control (i.e. background levels), and we computed the relative incidence (RI) of medically attended AEIs in the pre-exposure and risk periods compared with control. The duration of 7 days was chosen because mild or moderate AEIs tend to have an onset shortly after vaccination. In addition, in the early stages of the national vaccination programme rollout, the guidance provided by Joint Committee on Vaccination and Immunisation (JCVI) recommended a second dose of Comirnaty vaccine after 21 days. 

Model 1 included the vaccine main effect. We chose to use a calendar month effect to account for variation at different times of the rollout. While it is expected that some of the prespecified conditions may exhibit a seasonal pattern, we did not expect the model to show very strong seasonal patterns and therefore did not account for this by week. Model 2 additionally included an age interaction (with age centred at 50 years) to account for potential effects of the vaccine rollout by age groups. We chose to centre age at 50 years as this is close to the median age of our vaccinated populations, with an average age of 52 years for Comirnaty and 56 years for Vaxzevria COVID-19 vaccination. Finally, we performed Model 2 separately for the different categories of AEIs to explore differences between the safety profiles of the three brands of vaccines.

We carried out a sensitivity analysis where we repeated Model 2 but for a 21-day post vaccine risk period. We compared incidence in the control period with the pre-exposure period and three successive observational periods: (i) 0 to 7 days after vaccination (as in the main study), (ii) days 8–14 after vaccination and (iii) days 15–21 post vaccination. We hypothesised that the RI of AEIs would decline in successive weeks following the week of vaccination.

### Statistical analyses

We computed descriptive statistics to provide an overview of the demographic characteristics of the study sample. For missing data in the ethnicity variable, no imputation was required for modelling purposes. 

All statistical analyses for all models were conducted in R version 4.1.2 [21], using the following packages: *dplyr* (version 1.0.7) [22], *lubridate* (version 1.8.0) [23], *SCCS* (version 1.2) [24], and *tableone* (version 0.12.0) [25]. Graphical output was generated using *ggplot2* (version 3.3.5) [26].

## Results

### Sample demographics

**Figure 3:**
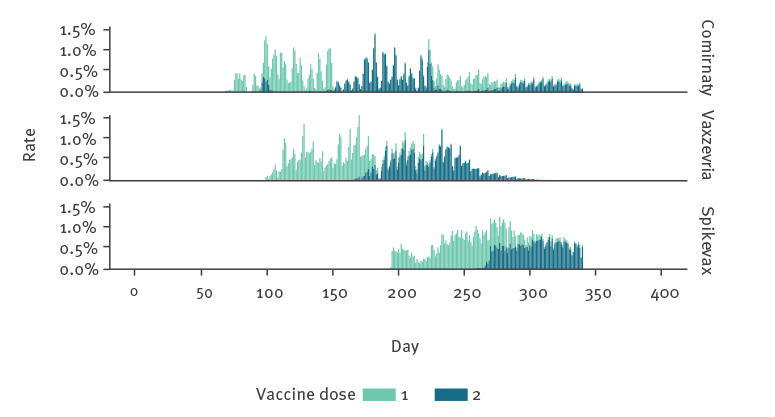
Time when individuals reporting adverse events of interest received their first and second doses of COVID-19 vaccine by brand, England, 1 October 2020–12 September 2021 (n = 1,287,771 doses)

The ORCHID cohort at the time of the study consisted of 7,952,861 individuals. Among them, 9.82% (n = 781,200) of these people reported a total of 1,482,273 medically attended AEIs during the study period, equating to 1.90 events per person who reported any AEI. Only 4.85% (n = 56,914) of these AEIs were reported within the first 7 days after vaccination. The mean age of this sample was 51.82 years (standard deviation (SD): 20.02 years), with a strong female preponderance (62.36% female) and a large majority were of white ethnicity (74.85%). Around three-quarters of the sample were double-vaccinated, with the percentage of individuals who received Comirnaty, Vaxzevria and Spikevax being 38.48%, 47.22% and 1.54%, respectively (**Table 2**). The time at which patients received their vaccinations during the study period is presented in **Figure 3**.

**Table 2:** Demographic characteristics of individuals reporting adverse events of interest following COVID-19 vaccination, England, 1 October 2020–12 September 2021 (n = 781,200)

Characteristics	Comirnaty n = 300,641		Vaxzevria n = 368,898		Spikevax n = 12,024		Unvaccinated n = 99,637	
	n	%	n	%	n	%	n	%
Age at study index date								
Mean in years (SD)	52.09	22.76	56.19	15.95	32.39	9.49	37.16	17.18
Sex								
Female	192,364	63.98	223,982	60.72	7,386	61.43	63,438	63.67
Male	108,277	36.02	144,916	39.28	4,638	38.57	36,199	36.33
Ethnicity								
White	226,147	75.22	287,569	77.95	8,384	69.73	62,629	62.86
Asian	19,939	6.63	22,354	6.06	792	6.59	7,722	7.75
Black	6,190	2.06	8,233	2.23	245	2.04	7,777	7.81
Mixed	3,399	1.13	3,400	0.92	220	1.83	2,703	2.71
Other	2,802	0.93	2,991	0.81	186	1.55	2,002	2.01
Missing	42,164	14.02	44,351	12.02	2,197	18.27	16,804	16.87
Index of Multiple Deprivation quintile								
1 – most deprived	50,606	16.83	64,157	17.39	1,931	16.06	30,872	30.98
2	55,542	18.47	66,510	18.03	2,391	19.89	23,302	23.39
3	60,069	19.98	73,129	19.82	2,196	18.26	17,284	17.35
4	64,612	21.49	79,883	21.65	2,496	20.76	15,004	15.06
5 – least deprived	69,784	23.21	85,188	23.09	3,009	25.02	13,150	13.20
Missing	28	0.01	31	0.01	1	0.01	25	0.03
Number of vaccine doses received								
0	0	0	0	0	0	0	99,637	100
1	41,826	13.91	30,103	8.16	3,426	28.49	0	0
2	258,815	86.09	338,795	91.84	8,598	71.51	0	0

COVID-19: coronavirus disease; SD: standard deviation.

This figure shows when individuals received their first and second vaccine dose. The time is days from the study start date of 1 October 2020.

### Frequencies of medically attended AEIs

The frequencies of medically attended AEIs reported within the study period are presented by condition and by vaccine brand in **Table 3**. There were consultations for almost all AEIs within the study period, with the highest frequencies observed for milder symptoms such as joint pain, abdominal pain, cough and headache. More severe conditions such as Guillain–Barré syndrome, myocarditis and pericarditis were relatively rare.

**Table 3:** Frequencies of medically attended adverse events of interest following COVID-19 vaccination reported at any point throughout the study period by condition and vaccine brand, England, 1 October 2020–12 September 2021 (n = 781,200 individuals)

Condition	Comirnaty (1 or 2 doses) n = 300,641		Vaxzevria (1 or 2 doses) n = 368,898		Spikevax (1 or 2 doses) n = 12,024		Unvaccinated n = 99,637
	n	Rate per 10,000 doses	n	Rate per 10,000 doses	n	Rate per 10,000 doses	n
General non-specific							
Asthenia	1,064	19.02	1,494	21.11	11	5.33	505
Fatigue	34,930	624.36	40,928	23.47	1,525	739.50	11,282
Fever	8,940	159.80	12,120	171.26	270	130.93	3,578
Fever with chills	28	0.50	29	0.41	0	0	5
Malaise	8,797	157.25	10,935	154.52	173	83.89	2,252
Oedema of face	60	1.07	101	1.43	4	1.94	19
Injection site							
Bruising	0	0	0	0	0	0	0
Burning	0	0	0	0	0	0	0
Erythema	9	0.16	25	0.35	1	0.48	5^a^
Induration	0	0	3	0.04	0	0	0
Inflammation	1	0.02	0	0	0	0	0
Irritation	0	0	0	0	0	0	0
Pain	16	0.29	57	0.81	2	0.97	6^a^
Pruritus	0	0	0	0	1	0.48	0
Rash	2	0.04	5	0.07	1	0.48	1^a^
Swelling	16	0.29	27	0.38	4	1.94	2^a^
Urticaria	0	0	3	0.04	0	0	0
Ear							
Tinnitus	7,972	142.50	11,901	168.17	368	178.45	2,081
Gastrointestinal							
Abdominal pain	76,997	1,376.28	92,299	1,304.22	3,587	1,739.40	31,389
Diarrhoea	26,181	467.97	32,112	453.76	744	360.78	6,124
Nausea	9,541	170.54	10,620	150.07	288	139.66	4,060
Vomiting	10,588	189.26	12,798	180.84	378	183.30	6,096
Immune							
Anaphylaxis	419	7.49	861	12.17	21	10.18	235
Hypersensitivity reactions	12,475	222.98	16,099	227.49	606	293.86	4,860
Lymphatic							
Lymphadenopathy	4,290	76.68	3,986	56.32	286	138.69	1,508
Metabolic/nutrition							
Decrease in appetite	4,746	84.83	5,899	83.36	90	43.64	1,872
Musculoskeletal							
Joint pain	89,366	1,597.37	124,710	1,762.20	2,470	1,197.75	19,813
Myalgia	15,357	274.50	17,003	240.26	109	52.86	1,358
Weakness	1,064	19.02	1,494	21.11	11	5.33	505
Neurological							
Bell’s palsy	1,199	21.43	1,661	23.47	50	24.25	425
Dizziness	27,802	496.95	30,702	433.83	659	24.25	6,026
Drowsiness	916	16.37	1,175	16.60	30	14.55	303
Guillain–Barré syndrome	91	1.63	221	3.12	0	0	62
Headache	60,901	1,088.58	74,663	1,055.02	3,365	1,631.75	22,855
Lethargy	1,786	31.92	2,166	30.61	53	25.70	496
Paraesthesia	6,533	116.77	9,394	132.74	223	108.14	1,865
Peripheral tremor	3,633	64.94	4,752	67.15	50	24.25	597
Psychiatric							
Insomnia	11,723	209.54	14,714	207.92	434	210.45	4,583
Respiratory							
Cough	67,336	1,203.60	94,675	1,337.80	1,716	832.12	17,926
Influenza-like illness	3,349	59.86	4,990	70.51	122	59.16	1,561
Sneezing	255	4.56	233	3.29	6	2.91	110
Sore throat	19,406	346.87	19,338	273.25	1,056	512.07	8,221
Skin							
Angioedema	647	11.56	1,003	14.17	25	12.12	214
Eruption of skin	49,270	880.68	57,713	815.51	2,075	1,006.21	14,383
Hyperhidrosis	828	14.80	623	8.80	34	16.49	420
Vascular							
Capillary leak syndrome	0	0	0	0	0	0	0
Myocarditis	220	3.93	258	3.65	8	3.88	46
Pericarditis	429	7.67	597	8.44	21	10.18	177

COVID-19: coronavirus disease.

^a^ As the self-controlled case series design compares the occurrence of adverse events of interest in the risk period to the control period to determine whether the vaccine may be associated with a higher risk of certain adverse events, no a priori assumptions are made on whether these events are causally related, nor were there any selection process for events to include or exclude. For this reason, such events can include those that happened following administration of other vaccines, and a small number of injection site reactions were observed in the unvaccinated group.

### Incidence of medically attended AEIs in pre-exposure and risk periods

In Model 1, we observed a slightly lower incidence of medically attended AEIs in the pre-exposure and risk periods for both Comirnaty and Vaxzevria compared with background levels, but a higher incidence of medically attended AEIs in the risk period following the second dose of Spikevax (**Table 4**).

**Table 4:** Relative incidence of medically attended adverse events of interest in the pre-exposure and risk periods following COVID-19 vaccination for both doses by vaccine brand, England, 1 October 2020–12 September 2021 (n = 1,304,447 events including control period)

Model	Comirnaty		Vaxzevria		Spikevax	
	RI	95% CI	RI	95% CI	RI	95% CI
**Model 1 **						
D1: −7 to −1	0.96***	0.94–0.98	0.96***	0.94–0.98	1.02	0.96–1.09
D1: 0 to 7	0.92***	0.90–0.94	0.96***	0.94–0.97	1.05	1.00–1.12
D2: −7 to −1	0.91***	0.89–0.93	0.96***	0.94–0.98	1.00	0.94–1.07
D2: 0 to 7	0.94***	0.92–0.96	0.95***	0.94–0.97	1.07*	1.01–1.14
**Model 2 **						
D1: −7 to −1	0.96***	0.94–0.98	0.97***	0.95–0.98	1.16	0.95–1.41
D1: 0 to 7	0.93***	0.91–0.94	0.97***	0.95–0.98	1.20*	1.00–1.44
D2: −7 to −1	0.92***	0.90–0.94	0.96***	0.95–0.98	1.07	0.95–1.35
D2: 0 to 7	0.96***	0.94–0.98	0.97***	0.95–0.98	1.13	0.91–1.39
D1: −7 to −1 x age	0.9978***	0.9970–0.9987	0.9988*	0.9978–0.9999	1.0094	0.9990–1.0199
D1: 0 to 7 x age	0.9973***	0.9965–0.9982	0.9982***	0.9973–0.9992	1.0035	0.9944–1.0128
D2: −7 to −1 x age	0.9981***	0.9972–0.9991	0.9993	0.9982–1.0004	1.0062	0.9932–1.0193
D2: 0 to 7 x age	0.9965***	0.9956–0.9973	0.9979***	0.9968–0.9989	0.9988	0.9874–1.0103

COVID-19: coronavirus disease; D1: vaccine dose 1; D2: vaccine dose 2.

Days –7 to –1 refers to the pre-exposure period and days 0 to 7 refers to the risk period.

Significance is denoted as follows: * p < 0.05, ** p < 0.01, *** p < 0.001.

Age centred at 50 years in Model 2.

After accounting for age with an age interaction effect (Model 2), the RI remained lower than background levels in the pre-exposure and risk periods for both Comirnaty and Vaxzevria, but there was a marginally higher RI following one dose of Spikevax. The significant age interaction effects indicated fewer medically attended AEIs were reported as age increased for individuals who received Comirnaty or Vaxzevria. We ran the models with age centred at 30 and 70 years to illustrate the differences in main effects for the different age groups. The RIs and 95% CIs are reported in Supplementary Tables S2–S3.

### Incidence of medically attended adverse events of interest by category

As the frequencies of medically attended AEIs among individuals who received the Spikevax vaccine were too low for many of the categories, we performed the secondary analysis only for Comirnaty and Vaxzevria.

Following the first dose of Vaxzevria, there was an increased presentation with general non-specific, injection site and skin conditions. Following both doses of Comirnaty, but only the first dose of Vaxzevria, there was an increased incidence in immune and lymphatic conditions (**Table 5**).

**Table 5:** Relative incidence of medically attended adverse events of interest in the pre-exposure and risk periods for both COVID-19 vaccine doses by condition and vaccine brand, England, 1 October 2020–12 September 2021 (n = 1,283,570 events including control period)

Condition	Comirnaty		Vaxzevria	
	RI	95% CI	RI	95% CI
General / injection site / skin				
D1: −7 to −1	0.97	0.92–1.01	0.96	0.92–1.00
D1: 0 to 7	0.98	0.94–1.02	1.07***	1.03–1.11
D2: −7 to −1	0.95*	0.90–0.99	0.98	0.94–1.03
D2: 0 to 7	0.98	0.94–1.03	0.96	0.93–1.01
Gastrointestinal / metabolic / nutrition				
D1: −7 to −1	0.89***	0.86–0.93	0.95**	0.91–0.99
D1: 0 to 7	0.87***	0.84–0.91	0.93***	0.90–0.97
D2: −7 to −1	0.88***	0.84–0.92	0.96*	0.92–1.00
D2: 0 to 7	0.87***	0.83–0.90	0.93***	0.89–0.97
Immune / lymphatic				
D1: −7 to −1	1.16**	1.04–1.28	1.20***	1.09–1.32
D1: 0 to 7	1.32***	1.20–1.45	1.55***	1.43–1.68
D2: −7 to −1	0.89	0.78–1.01	1.00	0.89–1.11
D2: 0 to 7	1.41***	1.28–1.56	1.07	0.97–1.78
Musculoskeletal				
D1: −7 to −1	1.01	0.96–1.06	1.01	0.97–1.05
D1: 0 to 7	0.92***	0.88–0.97	0.87***	0.84–0.91
D2: −7 to −1	0.92**	0.87–0.97	1.06**	102–1.11
D2: 0 to 7	0.97	0.92–1.01	1.00	0.96–1.04
Neurological / psychiatric				
D1: −7 to −1	0.99	0.95–1.03	0.94**	0.91–0.98
D1: 0 to 7	0.92***	0.88–0.95	1.00	0.97–1.04
D2: −7 to −1	0.91***	0.87–0.95	0.91***	0.87–0.94
D2: 0 to 7	0.94**	0.90–0.98	1.03	0.99–1.07
Respiratory / ear				
D1: −7 to −1	0.91***	0.86–0.95	0.87***	0.83–0.91
D1: 0 to 7	0.87***	0.83–0.91	0.82***	0.79–0.86
D2: −7 to −1	0.89***	0.84–0.93	0.90***	0.86–0.95
D2: 0 to 7	0.93**	0.88–0.97	0.84***	0.80–0.88
Vascular				
D1: −7 to −1	0.61	0.27–1.34	0.66	0.38–1.16
D1: 0 to 7	0.88	0.51–1.51	0.77	0.47–1.26
D2: −7 to −1	0.97	0.51–1.82	0.60	0.31–1.17
D2: 0 to 7	1.24	0.72–2.12	0.46*	0.23–0.94

COVID-19: coronavirus disease; D1: vaccine dose 1; D2: vaccine dose 2.

Days –7 to –1 refers to the pre-exposure period and days 0 to 7 refers to the risk period.

Significance is denoted as follows: * p < 0.05, ** p < 0.01, *** p < 0.001. Age centred at 50 years.

### Sensitivity analysis

Our sensitivity analysis showed that in the third observation period, i.e., days 15 to 21 after both doses of Comirnaty and Spikevax and after the first dose of Vaxzevria, the RI of AEIs were not significantly different from background levels. In days 8 to 14 and days 15 to 21 after the second dose of Vaxzevria, the RI of AEIs were slightly elevated as shown in Supplementary Table S4.

## Discussion

Our analysis compares the incidence of medically attended AEIs following COVID-19 vaccination to background levels in 781,200 individuals. Overall, there was a small decrease in medically attended AEIs post-vaccination, reported by just under 10% of the registered population. Among those who sought medical attention for any of the prespecified AEIs, each person on average presented two conditions which were coded into their clinical records. Most of these AEIs were not temporally associated with vaccination, and even those that occurred within 7 days of vaccination may not necessarily be causally related to vaccination. 

The incidence of medically attended AEIs was lower compared with background levels of presentation in the first 7 days post-vaccination after both first and second doses for Comirnaty and Vaxzevria in our model that included age effects. We found a 3–7% decrease in incidence of medically attended AEIs in the 7 days post-vaccination for Comirnaty and Vaxzevria, but a 20% increase following the first dose of Spikevax. Fewer medically attended AEIs were reported as age increased for both Comirnaty and Vaxzevria vaccines.

The safety profile varied slightly between different vaccine brands. The only notable differences were in the increased incidence of general non-specific, injection site and skin conditions following the first dose of Vaxzevria, as well as the increase incidence of immune and lymphatic conditions following the second dose of Comirnaty, which was not observed with Vaxzevria.

A strength of this study is the well-established ORCHID practice network [27], which has high data quality since GP’s receive feedback on the data they submit through practice visits (primarily virtual during the study period) and dashboards [28]. The design of NIMS has ensured that COVID-19 vaccination records are reliably captured and sent back into primary care CMRs. In addition, this system has ensured that only a very small proportion of people did not have their vaccine brand recorded (0.5% for the first dose and 0.6% for the second dose) compared with influenza vaccination previously reported using data from the same surveillance network (1.4%) [29].

Our study also had some limitations. Firstly, there are always uncertainties about data quality and whether all relevant events have been captured, which can result in an underestimation of the incidence of medically attended AEIs. In this study, only events requiring medical attention and involving a GP consultation have been captured. We also accept that we only captured medically attended AEIs, so our process would be classified by the EMA as passive surveillance. Our findings, as in many observational studies, likely represent only the tip of the ‘epidemiological iceberg’ [30], particularly for minor conditions. Where we have conducted enhanced passive surveillance, i.e. including a customised reporting card to capture adverse events, more events are recorded [14]. While a unique national health number (NHS number) exists for every citizen and a national demographic service facilitates identification of individuals and the matching with their health records, it is still possible for GP lists to be inflated, where a patient leaves but does not get deregistered. This could lead to inflation of our non-vaccinated group. It is also likely that the 7-day risk period selected did not capture all AEIs that may be associated with the vaccine. We used this window because it is the window selected by the EMA for surveillance of AEIs post-influenza vaccination [11]. We do not have access to data about whether those vaccinated were healthcare workers, of which most were vaccinated with the Comirnaty vaccine, and may have had greater exposure to the severe acute respiratory syndrome coronavirus 2 (SARS-CoV-2) virus, or reported more adverse events [31]. We have not taken into account SARS-CoV-2 infections before vaccination or during the study period, which can be associated with some of these AEIs, and it remains unknown whether prior infection is associated with a higher or lower incidence of AEIs. Additionally, we have not included hospital emergency attendances that were not subsequently recorded back in the primary care record.

Our sensitivity analysis suggested it was reasonable to focus on the first 7 days after vaccination, aligned with the EMA recommendations for enhanced surveillance post-influenza vaccination [10-12,14]. However, given the novelty of the COVID-19 vaccines and the suggestion that AEIs may have a higher incidence in the periods of 8 to 14 days and 15 to 21 days post-second vaccination for one of the vaccines, examination of risk periods beyond the initial 7 days should be considered in future analyses. In particular, a limitation of the 7-day risk period we used is that some of the more serious cardiovascular and neurological AEIs such as myocarditis and Guillain–Barré Syndrome are more likely to fall outside this window, with symptom onset often occurring more than 10 days and potentially up to several weeks post-vaccination [32-37].

We did not include several rare but serious adverse events associated with COVID-19 vaccines which have already been reported [15,38,39], and our method excluded conditions associated with mortality during the observation period to prevent violation of the event independence assumption of the SCCS design. Our overall conclusion about low RI of AEIs should not ignore these rare but important risks [15,39]. Few studies in the literature have examined subgroup or vaccine brand effects. Though others have reported more serious AEIs in males, studies present mixed findings over the effect of age [40,41]. Signals of lymphadenopathy and myocarditis have also been reported in a national study of the Comirnaty vaccine, but without a comparator [42]. Likewise, Bell’s palsy, paraesthesia, and Guillain–Barré syndrome have inconclusive associations with vaccination [43,44].

The reduction in incidence of AEIs within the first 7 days has been observed in other studies. It may represent a ‘healthy vaccinee effect’, a type of bias in which people who are unwell may avoid or delay vaccination [45]. General practice appointments dipped but then have recovered nearly back to normal following the COVID-19 pandemic, with a greater proportion of appointments taking place over the phone, which is a phenomenon that is observed both in the UK as well as globally [46,47]. We have seen no evidence to suggest that this would have differently affected the pre- and post-vaccination window.

Our results reporting the pattern of AEIs after COVID-19 vaccination could provide a benchmark for future years as COVID-19 becomes endemic and there will be a continued need for vaccination. It is possible that either enhanced passive surveillance, where questionnaires are additionally used [14] or adding text searches using natural language processing (NLP) might increase AEI capture [48]. One study increased AEI capture using NLP by around 15% [49]. Where we have conducted enhanced passive surveillance for influenza, we have detected more AEIs, particularly local reactions that may not have reached the threshold for medical attendance [14].

## Conclusion

While it is recognised that COVID-19 vaccines are associated with a small increase in incidence of rare but serious adverse events, there has been less reporting of other milder and more common AEIs. Against a list of prespecified medically attended AEIs, we found there was no increase in incidence following vaccination with either dose of Comirnaty or Vaxzevria, or the second dose of Spikevax. Standardised reporting of AEIs, possibly via sentinel systems, could provide safety data complementary to other mechanisms for monitoring vaccine safety.

## Supplementary Data

1559000 Supplement

## References

[1] Vasileiou E Simpson CR Shi T Kerr S Agrawal U Akbari A Interim findings from first-dose mass COVID-19 vaccination roll-out and COVID-19 hospital admissions in Scotland: a national prospective cohort study. Lancet. 2021;397(10285):1646-57. 10.1016/S0140-6736(21)00677-2 33901420 PMC8064669

[2] Agrawal U Katikireddi SV McCowan C Mulholland RH Azcoaga-Lorenzo A Amele S COVID-19 hospital admissions and deaths after BNT162b2 and ChAdOx1 nCoV-19 vaccinations in 2·57 million people in Scotland (EAVE II): a prospective cohort study. Lancet Respir Med. 2021;9(12):1439-49. 10.1016/S2213-2600(21)00380-5 34599903 PMC8480963

[3] Whitaker HJ Tsang RSM Byford R Andrews NJ Sherlock J Sebastian Pillai P Pfizer-BioNTech and Oxford AstraZeneca COVID-19 vaccine effectiveness and immune response amongst individuals in clinical risk groups. J Infect. 2022;84(5):675-83. 10.1016/j.jinf.2021.12.044 34990709 PMC8720678

[4] Kissling E Hooiveld M Martínez-Baz I Mazagatos C William N Vilcu AM Effectiveness of complete primary vaccination against COVID-19 at primary care and community level during predominant Delta circulation in Europe: multicentre analysis, I-MOVE-COVID-19 and ECDC networks, July to August 2021. Euro Surveill. 2022;27(21):2101104. 10.2807/1560-7917.ES.2022.27.21.2101104 35620997 PMC9137272

[5] European Medicines Agency (EMA). Comirnaty. Amsterdam: EMA; 2022. Available from: https://www.ema.europa.eu/en/medicines/human/EPAR/comirnaty

[6] European Medicines Agency (EMA). Vaxzevria (previously COVID-19 Vaccine AstraZeneca). Amsterdam: EMA; 2022. Available from: https://www.ema.europa.eu/en/medicines/human/EPAR/vaxzevria

[7] European Medicines Agency (EMA). Spikevax (previously COVID-19 Vaccine Moderna). Amsterdam: EMA; 2022. Available from: https://www.ema.europa.eu/en/medicines/human/EPAR/spikevax

[8] Department of Health and Social Care. Use of the AstraZeneca COVID-19 (AZD1222) vaccine: updated JCVI statement, 7 May 2021. London: gov.uk; 2021. Available from: https://www.gov.uk/government/publications/use-of-the-astrazeneca-covid-19-vaccine-jcvi-statement-7-may-2021/use-of-the-astrazeneca-covid-19-azd1222-vaccine-updated-jcvi-statement-7-may-2021

[9] Public Health England. COVID-19 vaccine surveillance strategy, March 2021. London: gov.uk; 2021. Available from: https://assets.publishing.service.gov.uk/government/uploads/system/uploads/attachment_data/file/974300/COVID-19_vaccine_surveillance_strategy_March21.pdf

[10] Li R Stewart B McNeil MM Duffy J Nelson J Kawai AT Post licensure surveillance of influenza vaccines in the Vaccine Safety Datalink in the 2013-2014 and 2014-2015 seasons. Pharmacoepidemiol Drug Saf. 2016;25(8):928-34. 10.1002/pds.3996 27037540 PMC10878475

[11] European Medicines Agency (EMA). Interim guidance on enhanced safety surveillance for seasonal influenza vaccines in the EU. Amsterdam: EMA; 2014. Available from: https://www.ema.europa.eu/en/interim-guidance-enhanced-safety-surveillance-seasonal-influenza-vaccines-eu

[12] Bollaerts K de Smedt T McGee C Emborg H-D Villa M Alexandridou M ADVANCE: Towards near real-time monitoring of vaccination coverage, benefits and risks using European electronic health record databases. Vaccine. 2020;38(Suppl 2):B76-83. 10.1016/j.vaccine.2019.08.012 31677951

[13] de Lusignan S Jones N Dorward J Byford R Liyanage H Briggs J The Oxford Royal College of General Practitioners Clinical Informatics Digital Hub: Protocol to Develop Extended COVID-19 Surveillance and Trial Platforms. JMIR Public Health Surveill. 2020;6(3):e19773. 10.2196/19773 32484782 PMC7333793

[14] de Lusignan S Damaso S Ferreira F Byford R McGee C Pathirannehelage S Brand-specific enhanced safety surveillance of GSK’s Fluarix Tetra seasonal influenza vaccine in England: 2017/2018 season. Hum Vaccin Immunother. 2020;16(8):1762-71. 10.1080/21645515.2019.1705112 32118513 PMC7482908

[15] Simpson CR Shi T Vasileiou E Katikireddi SV Kerr S Moore E First-dose ChAdOx1 and BNT162b2 COVID-19 vaccines and thrombocytopenic, thromboembolic and hemorrhagic events in Scotland. Nat Med. 2021;27(7):1290-7. 10.1038/s41591-021-01408-4 34108714 PMC8282499

[16] Kerr S Joy M Torabi F Bedston S Akbari A Agrawal U First dose ChAdOx1 and BNT162b2 COVID-19 vaccinations and cerebral venous sinus thrombosis: A pooled self-controlled case series study of 11.6 million individuals in England, Scotland, and Wales. PLoS Med. 2022;19(2):e1003927. 10.1371/journal.pmed.1003927 35192598 PMC8863261

[17] Tippu Z Correa A Liyanage H Burleigh D McGovern A Van Vlymen J Ethnicity recording in primary care computerised medical record systems: an ontological approach. J Innov Health Inform. 2017;23(4):920. 10.14236/jhi.v23i4.920 28346128

[18] Noble S, McLennan D, Noble M, Plunkett E, Gutacker N, Silk M, et al. The English Indices of Deprivation 2019: Research Report London: Ministry of Housing, Communities and Local Government; 2019. Available from: https://dera.ioe.ac.uk/34264/1/IoD2019_Research_Report.pdf

[19] Whitaker HJ Hocine MN Farrington CP . The methodology of self-controlled case series studies. Stat Methods Med Res. 2009;18(1):7-26. 10.1177/0962280208092342 18562396

[20] Petersen I Douglas I Whitaker H . Self controlled case series methods: an alternative to standard epidemiological study designs. BMJ. 2016;354:i4515. 10.1136/bmj.i4515 27618829

[21] R Core Team. R: A language and environment for statistical computing. Vienna: R Foundation for Statistical Computing; 2021. Available from: https://www.R-project.org

[22] Wickham H, François R, Henry L, Müller K. dplyr: A Grammar of Data Manipulation. 2021. Available from: https://CRAN.R-project.org/package=dplyr

[23] Grolemund G Wickham H . Dates and times made easy with lubridate. J Stat Softw. 2011;40(3):1-25. 10.18637/jss.v040.i03

[24] Weldeslassie YG, Whitaker H, Farrington P. SCCS: The self-controlled case series method. 2020. Available from: https://CRAN.R-project.org/package=SCCS

[25] Yoshida K, Bartel A. tableone: Create ‘Table 1’ to describe baseline characteristics with or without propensity score weights. R package version 0.12.0. 2020. Available from: https://CRAN.R-project.org/package=tableone

[26] Wickham H. ggplot2: Elegant Graphics for Data Analysis. New York: Springer-Verlag; 2016.

[27] de Lusignan S Lopez Bernal J Byford R Amirthalingam G Ferreira F Akinyemi O Influenza and respiratory virus surveillance, vaccine uptake, and effectiveness at a time of cocirculating COVID-19: Protocol for the English primary care sentinel system for 2020-2021. JMIR Public Health Surveill. 2021;7(2):e24341. 10.2196/24341 33605892 PMC7899204

[28] Pathirannehelage S Kumarapeli P Byford R Yonova I Ferreira F de Lusignan S . Uptake of a dashboard designed to give realtime feedback to a sentinel network about key data required for influenza vaccine effectiveness studies. Stud Health Technol Inform. 2018;247:161-5. 29677943

[29] de Lusignan S Tsang RSM Amirthalingam G Akinyemi O Sherlock J Tripathy M Adverse events of interest following influenza vaccination, a comparison of cell culture-based with egg-based alternatives: English sentinel network annual report paper 2019/20. Lancet Reg Health Eur. 2021;2:100029. 10.1016/j.lanepe.2021.100029 34557791 PMC8454842

[30] Last JM . Commentary: the iceberg revisited. Int J Epidemiol. 2013;42(6):1613-5. 10.1093/ije/dyt112 24415603

[31] Bedston S Akbari A Jarvis CI Lowthian E Torabi F North L COVID-19 vaccine uptake, effectiveness, and waning in 82,959 health care workers: A national prospective cohort study in Wales. Vaccine. 2022;40(8):1180-9. 10.1016/j.vaccine.2021.11.061 35042645 PMC8760602

[32] Husby A Hansen JV Fosbøl E Thiesson EM Madsen M Thomsen RW SARS-CoV-2 vaccination and myocarditis or myopericarditis: population based cohort study. BMJ. 2021;375:e068665. 10.1136/bmj-2021-068665 34916207 PMC8683843

[33] Karlstad Ø Hovi P Husby A Härkänen T Selmer RM Pihlström N SARS-CoV-2 vaccination and myocarditis in a Nordic cohort study of 23 million residents. JAMA Cardiol. 2022;7(6):600-12. 10.1001/jamacardio.2022.0583 35442390 PMC9021987

[34] Wong H-L Hu M Zhou CK Lloyd PC Amend KL Beachler DC Risk of myocarditis and pericarditis after the COVID-19 mRNA vaccination in the USA: a cohort study in claims databases. Lancet. 2022;399(10342):2191-9. 10.1016/S0140-6736(22)00791-7 35691322 PMC9183215

[35] Patone M Handunnetthi L Saatci D Pan J Katikireddi SV Razvi S Neurological complications after first dose of COVID-19 vaccines and SARS-CoV-2 infection. Nat Med. 2021;27(12):2144-53. 10.1038/s41591-021-01556-7 34697502 PMC8629105

[36] Frontera JA Tamborska AA Doheim MF Garcia-Azorin D Gezegen H Guekht A Neurological events reported after COVID-19 vaccines: an analysis of vaccine adverse event reporting system. Ann Neurol. 2022;91(6):756-71. 10.1002/ana.26339 35233819 PMC9082459

[37] Allahyari F Molaee H Hosseini Nejad J . Covid-19 vaccines and neurological complications: a systematic review. Z Naturforsch C J Biosci. 2023;78(1-2):1-8. Epub ahead of print. 10.1515/znc-2022-0092 36087300

[38] Andrews NJ Stowe J Ramsay MEB Miller E . Risk of venous thrombotic events and thrombocytopenia in sequential time periods after ChAdOx1 and BNT162b2 COVID-19 vaccines: A national cohort study in England. Lancet Reg Health Eur. 2022;13:100260. 10.1016/j.lanepe.2021.100260 34927118 PMC8668159

[39] Hippisley-Cox J Patone M Mei XW Saatci D Dixon S Khunti K Risk of thrombocytopenia and thromboembolism after covid-19 vaccination and SARS-CoV-2 positive testing: self-controlled case series study. BMJ. 2021;374:n1931. 10.1136/bmj.n1931 34446426 PMC8388189

[40] Xiong X Yuan J Li M Jiang B Lu ZK . Age and gender disparities in adverse events following COVID-19 vaccination: real-world evidence based on big data for risk management. Front Med (Lausanne). 2021;8:700014. 10.3389/fmed.2021.700014 34350199 PMC8326508

[41] Dagan N Barda N Balicer RD . Adverse effects after BNT162b2 vaccine and SARS-CoV-2 infection, according to age and sex. N Engl J Med. 2021;385(24):2299. 10.1056/NEJMc2115045 34706169 PMC8609601

[42] Barda N Dagan N Ben-Shlomo Y Kepten E Waxman J Ohana R Safety of the BNT162b2 mRNA Covid-19 vaccine in a nationwide setting. N Engl J Med. 2021;385(12):1078-90. 10.1056/NEJMoa2110475 34432976 PMC8427535

[43] Sodhi M Samii A Etminan M . A comparative safety study of reported neurological adverse events with three COVID-19 vaccines. J Neurol. 2022;269(5):2301-3. 10.1007/s00415-021-10919-6 34999959 PMC8742704

[44] Miller E . Rapid evaluation of the safety of COVID-19 vaccines: how well have we done? Clin Microbiol Infect. 2022;28(4):477-8. 10.1016/j.cmi.2021.12.018 34999173 PMC8733286

[45] Remschmidt C Wichmann O Harder T . Frequency and impact of confounding by indication and healthy vaccinee bias in observational studies assessing influenza vaccine effectiveness: a systematic review. BMC Infect Dis. 2015;15(1):429. 10.1186/s12879-015-1154-y 26474974 PMC4609091

[46] Joy M McGagh D Jones N Liyanage H Sherlock J Parimalanathan V Reorganisation of primary care for older adults during COVID-19: a cross-sectional database study in the UK. Br J Gen Pract. 2020;70(697):e540-7. 10.3399/bjgp20X710933 32661009 PMC7363277

[47] Tu K Sarkadi Kristiansson R Gronsbell J de Lusignan S Flottorp S Goh LH Changes in primary care visits arising from the COVID-19 pandemic: an international comparative study by the International Consortium of Primary Care Big Data Researchers (INTRePID). BMJ Open. 2022;12(5):e059130. 10.1136/bmjopen-2021-059130 35534063 PMC9086267

[48] Ford E Carroll JA Smith HE Scott D Cassell JA . Extracting information from the text of electronic medical records to improve case detection: a systematic review. J Am Med Inform Assoc. 2016;23(5):1007-15. 10.1093/jamia/ocv180 26911811 PMC4997034

[49] Deady M Ezzeldin H Cook K Billings D Pizarro J Plotogea AA The Food and Drug Administration biologics effectiveness and safety initiative facilitates detection of vaccine administrations from unstructured data in medical records through natural language processing. Front Digit Health. 2021;3:777905. 10.3389/fdgth.2021.777905 35005697 PMC8727347

[50] Tran Kiem C Andronico A Bosetti P Paireau J Alter L Boëlle P-Y Benefits and risks associated with different uses of the COVID-19 vaccine Vaxzevria: a modelling study, France, May to September 2021. Euro Surveill. 2021;26(26):2100533. 10.2807/1560-7917.ES.2021.26.26.2100533 34212840 PMC8326661

[51] Centers for Disease Control and Prevention (CDC). Advisory Committee on Immunization Practices (ACIP): Coronovirus Disease 2019 (COVID-19) Vaccines. ACIP Presentation Slides: June 23-25, 2021 Meeting. Atlanta: CDC; 2021. Available from: https://www.cdc.gov/vaccines/acip/meetings/slides-2021-06.html

