# Supplementary material for: Adverse events following first and second dose COVID-19 vaccination in England, October 2020 to September 2021: a national vaccine surveillance platform self-controlled case series study
Source: Euro Surveill. 2023 Jan 19;28(3):2200195. doi: 10.2807/1560-7917.ES.2023.28.3.2200195 (PMC9853944; doi:10.2807/1560-7917.ES.2023.28.3.2200195)
Supplement: Supplement [file 22-00195_LUSIGNAN_SUPPLEMENT.pdf]

Supplementary material

Disclaimer: This supplementary material is hosted by *Eurosurveillance* as supporting information alongside the article "*Adverse events following first and second dose COVID-19 vaccination in England 2020-2021: national vaccine surveillance platform self-controlled case series*", on behalf of the authors, who remain responsible for the accuracy and appropriateness of the content. The same standards for ethics, copyright, attributions and permissions as for the article apply. Supplements are not edited by *Eurosurveillance* and the journal is not responsible for the maintenance of any links or email addresses provided therein.

Table S1: SNOMED CT concept IDs used within the study.
Table S2: Model 2 results with age centred at 30.
Table S3: Model 2 results with age centred at 70 years.
Table S4: Model 2 results with the addition of risk periods of 8-14 days and 15-21 days after vaccination, relative incidence (95% confidence interval).

Supplementary Table S1. SNOMED CT concept IDs used within the study.

Clinical variable	Concept ID
Anaphylaxis	14654002
Anaphylaxis	39579001
Anaphylaxis	79337003
Anaphylaxis	81710009
Anaphylaxis	91941002
Anaphylaxis	111737003
Anaphylaxis	213320003
Anaphylaxis	241930003
Anaphylaxis	241931004
Anaphylaxis	241932006
Anaphylaxis	241933001
Anaphylaxis	241934007
Anaphylaxis	241935008
Anaphylaxis	241936009
Anaphylaxis	241937000
Anaphylaxis	241938005
Anaphylaxis	241939002
Anaphylaxis	241940000
Anaphylaxis	241941001
Anaphylaxis	241942008
Anaphylaxis	241952007
Anaphylaxis	241954008
Anaphylaxis	315334000
Anaphylaxis	373674001
Anaphylaxis	402390008
Anaphylaxis	402391007
Anaphylaxis	417516000
Anaphylaxis	419042001
Anaphylaxis	427833000
Anaphylaxis	427903006
Anaphylaxis	428795003
Anaphylaxis	429751004
Anaphylaxis	430980000
Anaphylaxis	441492003
Anaphylaxis	441495001
Anaphylaxis	441593005
Anaphylaxis	442052005
Anaphylaxis	715887004
Anaphylaxis	716374005
Anaphylaxis	719040004
Anaphylaxis	735173007
Anaphylaxis	735447008
Anaphylaxis	735448003

Anaphylaxis	735975001
Anaphylaxis	762455001
Anaphylaxis	871926004
Anaphylaxis	871930001
Anaphylaxis	1003758002
Anaphylaxis	139851000119105
Anaphylaxis	215661000000107
Anaphylaxis	215671000000100
Anaphylaxis	413881000000102
Anaphylaxis	470141000000104
Anaphylaxis	963681000000101
Anaphylaxis	963721000000108
Anaphylaxis	963731000000105
Angioedema	41291007
Angioedema	82966003
Angioedema	234619000
Angioedema	234620006
Angioedema	238694002
Angioedema	241955009
Angioedema	241956005
Angioedema	241957001
Angioedema	241958006
Angioedema	241959003
Angioedema	241960008
Angioedema	241962000
Angioedema	241963005
Angioedema	241964004
Angioedema	241965003
Angioedema	277785006
Angioedema	371627004
Angioedema	402382008
Angioedema	402387002
Angioedema	402388007
Angioedema	402389004
Angioedema	402401003
Angioedema	402404006
Angioedema	402405007
Angioedema	402406008
Angioedema	402407004
Angioedema	402411005
Angioedema	402602000
Angioedema	403398009
Angioedema	403607004
Angioedema	427167008

Angioedema	703795004
Angioedema	703801008
Angioedema	703802001
Angioedema	724831003
Angioedema	737557007
Angioedema	844575001
Angioedema	846577007
Angioedema	846579005
Angioedema	846758008
Angioedema	1003756003
Angioedema	757291000000109
Angioedema	757301000000108
Asthenia	13791008
Asthenia	18726006
Asthenia	161873000
Asthenia	161874006
Asthenia	206768006
Asthenia	207529006
Asthenia	207530001
Asthenia	248278004
Asthenia	250002000
Asthenia	271556007
Asthenia	271875007
Asthenia	272036004
Asthenia	274236006
Asthenia	373931001
Asthenia	439201000000104
Asthenia	501681000000107
Asthenia	501691000000109
Asthenia	502161000000101
Asthenia	502201000000109
Asthenia	15634971000119107
BellsPalsy	193093009
BellsPalsy	783257005
CapillaryLeakSyndrome	87730004
EruptionOfSkin	3358001
EruptionOfSkin	3533007
EruptionOfSkin	3755001
EruptionOfSkin	4244005
EruptionOfSkin	4776004
EruptionOfSkin	4859009
EruptionOfSkin	6048002
EruptionOfSkin	6111009
EruptionOfSkin	6618004

EruptionOfSkin	8007005
EruptionOfSkin	8197001
EruptionOfSkin	10401002
EruptionOfSkin	12019006
EruptionOfSkin	15576007
EruptionOfSkin	16341002
EruptionOfSkin	16429005
EruptionOfSkin	19362000
EruptionOfSkin	19431000
EruptionOfSkin	19514005
EruptionOfSkin	20343006
EruptionOfSkin	22583005
EruptionOfSkin	22818000
EruptionOfSkin	22972008
EruptionOfSkin	23067006
EruptionOfSkin	23346002
EruptionOfSkin	23854007
EruptionOfSkin	25847004
EruptionOfSkin	25858008
EruptionOfSkin	27520001
EruptionOfSkin	27620007
EruptionOfSkin	28414003
EruptionOfSkin	28840001
EruptionOfSkin	28926001
EruptionOfSkin	30242009
EruptionOfSkin	30704002
EruptionOfSkin	31922003
EruptionOfSkin	32698008
EruptionOfSkin	33090006
EruptionOfSkin	33339001
EruptionOfSkin	34630004
EruptionOfSkin	34730008
EruptionOfSkin	37042000
EruptionOfSkin	37218007
EruptionOfSkin	37702000
EruptionOfSkin	38360005
EruptionOfSkin	38921001
EruptionOfSkin	40175007
EruptionOfSkin	41156006
EruptionOfSkin	41890004
EruptionOfSkin	42442001
EruptionOfSkin	43118004
EruptionOfSkin	43591003
EruptionOfSkin	44509000

EruptionOfSkin	46629009
EruptionOfSkin	47277009
EruptionOfSkin	49882001
EruptionOfSkin	51048002
EruptionOfSkin	51057008
EruptionOfSkin	51696001
EruptionOfSkin	52230004
EruptionOfSkin	54116000
EruptionOfSkin	54385001
EruptionOfSkin	54792008
EruptionOfSkin	55608001
EruptionOfSkin	55846006
EruptionOfSkin	56253000
EruptionOfSkin	56454009
EruptionOfSkin	56940005
EruptionOfSkin	58143000
EruptionOfSkin	58767000
EruptionOfSkin	58872001
EruptionOfSkin	59032001
EruptionOfSkin	59172008
EruptionOfSkin	59721007
EruptionOfSkin	61099007
EruptionOfSkin	62227009
EruptionOfSkin	64144002
EruptionOfSkin	64540004
EruptionOfSkin	65038009
EruptionOfSkin	67081008
EruptionOfSkin	67896006
EruptionOfSkin	68166002
EruptionOfSkin	68266006
EruptionOfSkin	69047008
EruptionOfSkin	73442001
EruptionOfSkin	73532000
EruptionOfSkin	73658009
EruptionOfSkin	73692007
EruptionOfSkin	75867005
EruptionOfSkin	76097009
EruptionOfSkin	76401005
EruptionOfSkin	77252004
EruptionOfSkin	77300003
EruptionOfSkin	77470003
EruptionOfSkin	78631004
EruptionOfSkin	79372000
EruptionOfSkin	79893008

EruptionOfSkin	80346008
EruptionOfSkin	81271001
EruptionOfSkin	81856009
EruptionOfSkin	82721003
EruptionOfSkin	83627000
EruptionOfSkin	83839005
EruptionOfSkin	84036008
EruptionOfSkin	84939004
EruptionOfSkin	85207007
EruptionOfSkin	86425006
EruptionOfSkin	86487001
EruptionOfSkin	86563000
EruptionOfSkin	86735004
EruptionOfSkin	87144001
EruptionOfSkin	88233000
EruptionOfSkin	88697005
EruptionOfSkin	89414003
EruptionOfSkin	90433002
EruptionOfSkin	90911007
EruptionOfSkin	91487003
EruptionOfSkin	95328003
EruptionOfSkin	95331002
EruptionOfSkin	95332009
EruptionOfSkin	95371007
EruptionOfSkin	102606000
EruptionOfSkin	109251008
EruptionOfSkin	109254000
EruptionOfSkin	110989007
EruptionOfSkin	111188005
EruptionOfSkin	111873003
EruptionOfSkin	111931007
EruptionOfSkin	123702007
EruptionOfSkin	123705009
EruptionOfSkin	123795004
EruptionOfSkin	128191000
EruptionOfSkin	139914006
EruptionOfSkin	141617003
EruptionOfSkin	155667007
EruptionOfSkin	162415008
EruptionOfSkin	164430003
EruptionOfSkin	164623002
EruptionOfSkin	186357007
EruptionOfSkin	186504007
EruptionOfSkin	186535001

EruptionOfSkin	186562009
EruptionOfSkin	186567003
EruptionOfSkin	186580000
EruptionOfSkin	186581001
EruptionOfSkin	186668002
EruptionOfSkin	187425001
EruptionOfSkin	187428004
EruptionOfSkin	195900001
EruptionOfSkin	199191003
EruptionOfSkin	199192005
EruptionOfSkin	199193000
EruptionOfSkin	199194006
EruptionOfSkin	199195007
EruptionOfSkin	199196008
EruptionOfSkin	200766001
EruptionOfSkin	200767005
EruptionOfSkin	200832000
EruptionOfSkin	200892002
EruptionOfSkin	200893007
EruptionOfSkin	200945002
EruptionOfSkin	200948000
EruptionOfSkin	200950008
EruptionOfSkin	200954004
EruptionOfSkin	200962007
EruptionOfSkin	200963002
EruptionOfSkin	200964008
EruptionOfSkin	200965009
EruptionOfSkin	200966005
EruptionOfSkin	200967001
EruptionOfSkin	200968006
EruptionOfSkin	200969003
EruptionOfSkin	200972005
EruptionOfSkin	200973000
EruptionOfSkin	200974006
EruptionOfSkin	200976008
EruptionOfSkin	200981004
EruptionOfSkin	200982006
EruptionOfSkin	200984007
EruptionOfSkin	200985008
EruptionOfSkin	200993008
EruptionOfSkin	200994002
EruptionOfSkin	200999007
EruptionOfSkin	201000006
EruptionOfSkin	201001005

EruptionOfSkin	201002003
EruptionOfSkin	201015007
EruptionOfSkin	201016008
EruptionOfSkin	201017004
EruptionOfSkin	201382007
EruptionOfSkin	201383002
EruptionOfSkin	201384008
EruptionOfSkin	204919003
EruptionOfSkin	206861007
EruptionOfSkin	206863005
EruptionOfSkin	213323001
EruptionOfSkin	232242004
EruptionOfSkin	233625007
EruptionOfSkin	235049008
EruptionOfSkin	237112004
EruptionOfSkin	238370005
EruptionOfSkin	238373007
EruptionOfSkin	238392009
EruptionOfSkin	238425003
EruptionOfSkin	238453003
EruptionOfSkin	238485000
EruptionOfSkin	238525001
EruptionOfSkin	238526000
EruptionOfSkin	238527009
EruptionOfSkin	238546005
EruptionOfSkin	238550003
EruptionOfSkin	238586002
EruptionOfSkin	238587006
EruptionOfSkin	238588001
EruptionOfSkin	238589009
EruptionOfSkin	238600001
EruptionOfSkin	238601002
EruptionOfSkin	238602009
EruptionOfSkin	238603004
EruptionOfSkin	238609000
EruptionOfSkin	238611009
EruptionOfSkin	238612002
EruptionOfSkin	238613007
EruptionOfSkin	238614001
EruptionOfSkin	238615000
EruptionOfSkin	238616004
EruptionOfSkin	238617008
EruptionOfSkin	238618003
EruptionOfSkin	238619006

EruptionOfSkin	238620000
EruptionOfSkin	238621001
EruptionOfSkin	238622008
EruptionOfSkin	238628007
EruptionOfSkin	238629004
EruptionOfSkin	238640007
EruptionOfSkin	238644003
EruptionOfSkin	238645002
EruptionOfSkin	238646001
EruptionOfSkin	238647005
EruptionOfSkin	238648000
EruptionOfSkin	238649008
EruptionOfSkin	238651007
EruptionOfSkin	238652000
EruptionOfSkin	238653005
EruptionOfSkin	238654004
EruptionOfSkin	238655003
EruptionOfSkin	238656002
EruptionOfSkin	238657006
EruptionOfSkin	238658001
EruptionOfSkin	238659009
EruptionOfSkin	238660004
EruptionOfSkin	238661000
EruptionOfSkin	238662007
EruptionOfSkin	238663002
EruptionOfSkin	238664008
EruptionOfSkin	238665009
EruptionOfSkin	238666005
EruptionOfSkin	238667001
EruptionOfSkin	238668006
EruptionOfSkin	238669003
EruptionOfSkin	238670002
EruptionOfSkin	238671003
EruptionOfSkin	238682006
EruptionOfSkin	238711000
EruptionOfSkin	238754005
EruptionOfSkin	238783008
EruptionOfSkin	238812004
EruptionOfSkin	238813009
EruptionOfSkin	238814003
EruptionOfSkin	238815002
EruptionOfSkin	238816001
EruptionOfSkin	238820002
EruptionOfSkin	238924007

EruptionOfSkin	238988008
EruptionOfSkin	238989000
EruptionOfSkin	238991008
EruptionOfSkin	238992001
EruptionOfSkin	238994000
EruptionOfSkin	238995004
EruptionOfSkin	238997007
EruptionOfSkin	239098009
EruptionOfSkin	239099001
EruptionOfSkin	239100009
EruptionOfSkin	239101008
EruptionOfSkin	239103006
EruptionOfSkin	239104000
EruptionOfSkin	239802003
EruptionOfSkin	239803008
EruptionOfSkin	239804002
EruptionOfSkin	239812005
EruptionOfSkin	239813000
EruptionOfSkin	240302002
EruptionOfSkin	240467006
EruptionOfSkin	240469009
EruptionOfSkin	240482001
EruptionOfSkin	240483006
EruptionOfSkin	240484000
EruptionOfSkin	240485004
EruptionOfSkin	240546009
EruptionOfSkin	240558009
EruptionOfSkin	240559001
EruptionOfSkin	240560006
EruptionOfSkin	240561005
EruptionOfSkin	240562003
EruptionOfSkin	240656001
EruptionOfSkin	240673004
EruptionOfSkin	240677003
EruptionOfSkin	240711004
EruptionOfSkin	240854000
EruptionOfSkin	240855004
EruptionOfSkin	240856003
EruptionOfSkin	240857007
EruptionOfSkin	240860000
EruptionOfSkin	247447004
EruptionOfSkin	247466004
EruptionOfSkin	247470007
EruptionOfSkin	247471006

EruptionOfSkin	248413004
EruptionOfSkin	254175006
EruptionOfSkin	262538008
EruptionOfSkin	266128007
EruptionOfSkin	266496009
EruptionOfSkin	267801007
EruptionOfSkin	268911002
EruptionOfSkin	270497005
EruptionOfSkin	271756005
EruptionOfSkin	271757001
EruptionOfSkin	271807003
EruptionOfSkin	274119009
EruptionOfSkin	274244006
EruptionOfSkin	275447008
EruptionOfSkin	275955005
EruptionOfSkin	276719000
EruptionOfSkin	277171005
EruptionOfSkin	277805004
EruptionOfSkin	278041003
EruptionOfSkin	284078000
EruptionOfSkin	289482006
EruptionOfSkin	289857000
EruptionOfSkin	289858005
EruptionOfSkin	289859002
EruptionOfSkin	289860007
EruptionOfSkin	289861006
EruptionOfSkin	297941009
EruptionOfSkin	297958004
EruptionOfSkin	301447009
EruptionOfSkin	304386008
EruptionOfSkin	309473001
EruptionOfSkin	312106007
EruptionOfSkin	312360009
EruptionOfSkin	314901000
EruptionOfSkin	367484009
EruptionOfSkin	367537006
EruptionOfSkin	396349005
EruptionOfSkin	396350005
EruptionOfSkin	397515005
EruptionOfSkin	397741002
EruptionOfSkin	397845003
EruptionOfSkin	398591002
EruptionOfSkin	398600002
EruptionOfSkin	399992009

EruptionOfSkin	400018004
EruptionOfSkin	400085009
EruptionOfSkin	400108007
EruptionOfSkin	400126005
EruptionOfSkin	400201008
EruptionOfSkin	400990009
EruptionOfSkin	400991008
EruptionOfSkin	402177006
EruptionOfSkin	402205005
EruptionOfSkin	402212001
EruptionOfSkin	402238001
EruptionOfSkin	402239009
EruptionOfSkin	402260003
EruptionOfSkin	402292002
EruptionOfSkin	402296004
EruptionOfSkin	402297008
EruptionOfSkin	402298003
EruptionOfSkin	402300003
EruptionOfSkin	402307000
EruptionOfSkin	402308005
EruptionOfSkin	402309002
EruptionOfSkin	402310007
EruptionOfSkin	402311006
EruptionOfSkin	402312004
EruptionOfSkin	402313009
EruptionOfSkin	402326008
EruptionOfSkin	402327004
EruptionOfSkin	402328009
EruptionOfSkin	402340009
EruptionOfSkin	402348002
EruptionOfSkin	402349005
EruptionOfSkin	402359006
EruptionOfSkin	402643000
EruptionOfSkin	402644006
EruptionOfSkin	402663007
EruptionOfSkin	402744003
EruptionOfSkin	402745002
EruptionOfSkin	402747005
EruptionOfSkin	402748000
EruptionOfSkin	402836009
EruptionOfSkin	402860008
EruptionOfSkin	402902002
EruptionOfSkin	402903007
EruptionOfSkin	402915006

EruptionOfSkin	402958005
EruptionOfSkin	402999004
EruptionOfSkin	403194002
EruptionOfSkin	403209006
EruptionOfSkin	403210001
EruptionOfSkin	403214005
EruptionOfSkin	403215006
EruptionOfSkin	403325003
EruptionOfSkin	403372003
EruptionOfSkin	403373008
EruptionOfSkin	403426003
EruptionOfSkin	403606008
EruptionOfSkin	403609001
EruptionOfSkin	403610006
EruptionOfSkin	403612003
EruptionOfSkin	403613008
EruptionOfSkin	403617009
EruptionOfSkin	403620001
EruptionOfSkin	403622009
EruptionOfSkin	403623004
EruptionOfSkin	403624005
EruptionOfSkin	403626007
EruptionOfSkin	403638003
EruptionOfSkin	403640008
EruptionOfSkin	403868009
EruptionOfSkin	404102002
EruptionOfSkin	409642009
EruptionOfSkin	410482007
EruptionOfSkin	420281004
EruptionOfSkin	421512008
EruptionOfSkin	423333008
EruptionOfSkin	424306000
EruptionOfSkin	428633000
EruptionOfSkin	442081006
EruptionOfSkin	442279002
EruptionOfSkin	444100007
EruptionOfSkin	700127007
EruptionOfSkin	700298002
EruptionOfSkin	700346002
EruptionOfSkin	702617007
EruptionOfSkin	707247006
EruptionOfSkin	707248001
EruptionOfSkin	707249009
EruptionOfSkin	707250009

EruptionOfSkin	711435001
EruptionOfSkin	713224001
EruptionOfSkin	713316008
EruptionOfSkin	713528004
EruptionOfSkin	717055000
EruptionOfSkin	717061002
EruptionOfSkin	718068005
EruptionOfSkin	718215008
EruptionOfSkin	720493003
EruptionOfSkin	721171007
EruptionOfSkin	721180007
EruptionOfSkin	721212001
EruptionOfSkin	721542002
EruptionOfSkin	721543007
EruptionOfSkin	722391005
EruptionOfSkin	723003004
EruptionOfSkin	723010005
EruptionOfSkin	723011009
EruptionOfSkin	723012002
EruptionOfSkin	723013007
EruptionOfSkin	723014001
EruptionOfSkin	723015000
EruptionOfSkin	723166008
EruptionOfSkin	724465009
EruptionOfSkin	724510007
EruptionOfSkin	724511006
EruptionOfSkin	724833000
EruptionOfSkin	724854007
EruptionOfSkin	724855008
EruptionOfSkin	724877007
EruptionOfSkin	725119006
EruptionOfSkin	725148000
EruptionOfSkin	726476005
EruptionOfSkin	733110004
EruptionOfSkin	733206005
EruptionOfSkin	735958007
EruptionOfSkin	737346001
EruptionOfSkin	762543009
EruptionOfSkin	763767006
EruptionOfSkin	765782003
EruptionOfSkin	765783008
EruptionOfSkin	768946000
EruptionOfSkin	768961004
EruptionOfSkin	768962006

EruptionOfSkin	774211005
EruptionOfSkin	778075008
EruptionOfSkin	784327005
EruptionOfSkin	784328000
EruptionOfSkin	784339002
EruptionOfSkin	827044000
EruptionOfSkin	827051009
EruptionOfSkin	827052002
EruptionOfSkin	827160004
EruptionOfSkin	846742005
EruptionOfSkin	863909002
EruptionOfSkin	870328002
EruptionOfSkin	870728004
EruptionOfSkin	870729007
EruptionOfSkin	870765002
EruptionOfSkin	870766001
EruptionOfSkin	897046009
EruptionOfSkin	1002229008
EruptionOfSkin	46801000000100
EruptionOfSkin	75221000000100
EruptionOfSkin	79491000000104
EruptionOfSkin	83751000000105
EruptionOfSkin	124911000119100
EruptionOfSkin	208931000000104
EruptionOfSkin	239961000000106
EruptionOfSkin	239971000000104
EruptionOfSkin	239981000000102
EruptionOfSkin	307891000000103
EruptionOfSkin	330821000119100
EruptionOfSkin	330841000119106
EruptionOfSkin	330851000119108
EruptionOfSkin	330871000119104
EruptionOfSkin	390961000000102
EruptionOfSkin	409881000000101
EruptionOfSkin	419081000000105
EruptionOfSkin	419171000000107
EruptionOfSkin	431621000000107
EruptionOfSkin	444331000000104
EruptionOfSkin	450401000000107
EruptionOfSkin	451321000000101
EruptionOfSkin	462821000000106
EruptionOfSkin	463161000000104
EruptionOfSkin	478841000000108
EruptionOfSkin	496451000000100

EruptionOfSkin	496461000000102
EruptionOfSkin	502281000000104
EruptionOfSkin	502681000000101
EruptionOfSkin	502851000000106
EruptionOfSkin	509441000000101
EruptionOfSkin	538331000000101
EruptionOfSkin	538361000000106
EruptionOfSkin	538371000000104
EruptionOfSkin	546381000000102
EruptionOfSkin	547651000000101
EruptionOfSkin	547661000000103
EruptionOfSkin	547671000000105
EruptionOfSkin	570841000000103
EruptionOfSkin	584601000000104
EruptionOfSkin	590801000000107
EruptionOfSkin	644681000000105
EruptionOfSkin	644691000000107
EruptionOfSkin	674651000000100
EruptionOfSkin	674701000000108
EruptionOfSkin	684871000000103
EruptionOfSkin	692621000000106
EruptionOfSkin	701371000000103
EruptionOfSkin	704411000000102
EruptionOfSkin	803991000000104
EruptionOfSkin	855741000000103
EruptionOfSkin	884341000000109
EruptionOfSkin	891001000000109
EruptionOfSkin	891091000000102
EruptionOfSkin	891191000000103
EruptionOfSkin	1583831000006118
EruptionOfSkin	1863241000006113
EruptionOfSkin	1863251000006110
EruptionOfSkin	1876471000006117
EruptionOfSkin	10629311000119107
EruptionOfSkin	10759761000119100
Fever	7520000
Fever	9619006
Fever	10301003
Fever	14683004
Fever	27578005
Fever	30571006
Fever	31120005
Fever	37141005
Fever	39002001

Fever	42136008
Fever	43626008
Fever	46107006
Fever	49513005
Fever	50177009
Fever	52635002
Fever	52715007
Fever	54962007
Fever	58827009
Fever	60025004
Fever	61525000
Fever	63993003
Fever	64882008
Fever	70612009
Fever	74873003
Fever	77957000
Fever	78168002
Fever	88808006
Fever	95627000
Fever	95908009
Fever	102496004
Fever	103001002
Fever	111950007
Fever	135883003
Fever	139106004
Fever	139108003
Fever	161850008
Fever	161852000
Fever	164285001
Fever	164288004
Fever	164289007
Fever	164298005
Fever	164299002
Fever	164301009
Fever	164304001
Fever	164305000
Fever	164307008
Fever	164308003
Fever	164309006
Fever	164311002
Fever	164312009
Fever	164314005
Fever	164315006
Fever	164316007

Fever	164317003
Fever	164318008
Fever	186694006
Fever	187374004
Fever	206758001
Fever	206760004
Fever	206761000
Fever	206762007
Fever	206763002
Fever	206764008
Fever	213026003
Fever	240822009
Fever	240827003
Fever	248425001
Fever	248426000
Fever	248427009
Fever	248432005
Fever	248433000
Fever	248434006
Fever	248435007
Fever	248436008
Fever	248437004
Fever	248438009
Fever	248439001
Fever	248440004
Fever	248441000
Fever	248442007
Fever	248443002
Fever	248444008
Fever	248445009
Fever	248446005
Fever	248447001
Fever	248449003
Fever	248450003
Fever	248451004
Fever	248454007
Fever	267340006
Fever	268893004
Fever	271749004
Fever	271750004
Fever	271751000
Fever	271752007
Fever	271753002
Fever	271754008

Fever	271755009
Fever	271897009
Fever	274234009
Fever	274235005
Fever	274308003
Fever	274640006
Fever	304213008
Fever	308893005
Fever	365977007
Fever	365989009
Fever	367493005
Fever	373904004
Fever	373906002
Fever	386661006
Fever	405501007
Fever	405543000
Fever	409702008
Fever	420079008
Fever	421154002
Fever	426000000
Fever	430691009
Fever	449129007
Fever	698578005
Fever	698579002
Fever	698580004
Fever	698581000
Fever	704425001
Fever	713014002
Fever	713731001
Fever	719398004
Fever	722892007
Fever	10151000132103
Fever	119751000146104
Fever	123471000119103
Fever	130091000119103
Fever	391061000000106
Fever	396991000000105
Fever	441761000000105
Fever	450361000000103
Fever	465301000000100
Fever	468191000000103
Fever	495721000000108
Fever	495731000000105
Fever	495741000000101

Fever	502181000000105
Fever	502191000000107
Fever	540581000000105
Fever	548321000000102
Fever	571041000000100
Fever	571051000000102
Fever	582151000000103
Fever	598631000000100
Fever	611081000000107
Fever	832461000000104
Fever	832471000000106
FindingOfAbdominalPain	5268006
FindingOfAbdominalPain	6548007
FindingOfAbdominalPain	6744007
FindingOfAbdominalPain	7093002
FindingOfAbdominalPain	9991008
FindingOfAbdominalPain	15803009
FindingOfAbdominalPain	16844001
FindingOfAbdominalPain	17329003
FindingOfAbdominalPain	21005005
FindingOfAbdominalPain	21522001
FindingOfAbdominalPain	32096006
FindingOfAbdominalPain	35363006
FindingOfAbdominalPain	36729000
FindingOfAbdominalPain	37389005
FindingOfAbdominalPain	38654001
FindingOfAbdominalPain	39402007
FindingOfAbdominalPain	43548008
FindingOfAbdominalPain	45979003
FindingOfAbdominalPain	60043000
FindingOfAbdominalPain	62647006
FindingOfAbdominalPain	65754002
FindingOfAbdominalPain	71850005
FindingOfAbdominalPain	74704000
FindingOfAbdominalPain	75879005
FindingOfAbdominalPain	77880009
FindingOfAbdominalPain	79922009
FindingOfAbdominalPain	83132003
FindingOfAbdominalPain	86208007
FindingOfAbdominalPain	88522004
FindingOfAbdominalPain	102613000
FindingOfAbdominalPain	102614006
FindingOfAbdominalPain	102615007
FindingOfAbdominalPain	102626001

FindingOfAbdominalPain	102628000
FindingOfAbdominalPain	102631004
FindingOfAbdominalPain	102831002
FindingOfAbdominalPain	111985007
FindingOfAbdominalPain	116290004
FindingOfAbdominalPain	162038003
FindingOfAbdominalPain	162040008
FindingOfAbdominalPain	162042000
FindingOfAbdominalPain	162046002
FindingOfAbdominalPain	162047006
FindingOfAbdominalPain	162048001
FindingOfAbdominalPain	162049009
FindingOfAbdominalPain	162050009
FindingOfAbdominalPain	163223001
FindingOfAbdominalPain	196791007
FindingOfAbdominalPain	207124007
FindingOfAbdominalPain	207127000
FindingOfAbdominalPain	207131006
FindingOfAbdominalPain	207155001
FindingOfAbdominalPain	207156000
FindingOfAbdominalPain	207157009
FindingOfAbdominalPain	207158004
FindingOfAbdominalPain	207161003
FindingOfAbdominalPain	207200008
FindingOfAbdominalPain	207201007
FindingOfAbdominalPain	207205003
FindingOfAbdominalPain	207207006
FindingOfAbdominalPain	207209009
FindingOfAbdominalPain	207211000
FindingOfAbdominalPain	207212007
FindingOfAbdominalPain	207213002
FindingOfAbdominalPain	207218006
FindingOfAbdominalPain	207219003
FindingOfAbdominalPain	207220009
FindingOfAbdominalPain	207230000
FindingOfAbdominalPain	230648001
FindingOfAbdominalPain	235841007
FindingOfAbdominalPain	236554001
FindingOfAbdominalPain	236555000
FindingOfAbdominalPain	238970000
FindingOfAbdominalPain	247352008
FindingOfAbdominalPain	247353003
FindingOfAbdominalPain	247355005
FindingOfAbdominalPain	247361008

FindingOfAbdominalPain	247362001
FindingOfAbdominalPain	266599000
FindingOfAbdominalPain	271681002
FindingOfAbdominalPain	271832001
FindingOfAbdominalPain	271853005
FindingOfAbdominalPain	271857006
FindingOfAbdominalPain	271858001
FindingOfAbdominalPain	274256007
FindingOfAbdominalPain	274279008
FindingOfAbdominalPain	274280006
FindingOfAbdominalPain	274291004
FindingOfAbdominalPain	275406005
FindingOfAbdominalPain	279028009
FindingOfAbdominalPain	285387005
FindingOfAbdominalPain	285388000
FindingOfAbdominalPain	286993001
FindingOfAbdominalPain	289669005
FindingOfAbdominalPain	289670006
FindingOfAbdominalPain	289732000
FindingOfAbdominalPain	289817003
FindingOfAbdominalPain	289818008
FindingOfAbdominalPain	289844008
FindingOfAbdominalPain	289845009
FindingOfAbdominalPain	301367001
FindingOfAbdominalPain	301368006
FindingOfAbdominalPain	301715003
FindingOfAbdominalPain	301717006
FindingOfAbdominalPain	304542004
FindingOfAbdominalPain	307722004
FindingOfAbdominalPain	307724003
FindingOfAbdominalPain	307725002
FindingOfAbdominalPain	309475008
FindingOfAbdominalPain	309737007
FindingOfAbdominalPain	311813008
FindingOfAbdominalPain	314041007
FindingOfAbdominalPain	314212008
FindingOfAbdominalPain	371102005
FindingOfAbdominalPain	414991007
FindingOfAbdominalPain	426135001
FindingOfAbdominalPain	438506002
FindingOfAbdominalPain	439469002
FindingOfAbdominalPain	443503005
FindingOfAbdominalPain	444746004
FindingOfAbdominalPain	449890002

FindingOfAbdominalPain	722876002
FindingOfAbdominalPain	784285002
FindingOfAbdominalPain	830292003
FindingOfAbdominalPain	830293008
FindingOfAbdominalPain	838410008
FindingOfAbdominalPain	838411007
FindingOfAbdominalPain	1119217009
FindingOfAbdominalPain	1119218004
FindingOfAbdominalPain	125661000119107
FindingOfAbdominalPain	136051000119105
FindingOfAbdominalPain	136571000119109
FindingOfAbdominalPain	137891000119105
FindingOfAbdominalPain	164451000000109
FindingOfAbdominalPain	171731000000100
FindingOfAbdominalPain	390251000000109
FindingOfAbdominalPain	440501000000108
FindingOfAbdominalPain	444651000000101
FindingOfAbdominalPain	462691000000108
FindingOfAbdominalPain	469531000000101
FindingOfAbdominalPain	498491000000107
FindingOfAbdominalPain	498521000000105
FindingOfAbdominalPain	498731000000103
FindingOfAbdominalPain	498741000000107
FindingOfAbdominalPain	498751000000105
FindingOfAbdominalPain	499071000000107
FindingOfAbdominalPain	499081000000109
FindingOfAbdominalPain	499111000000101
FindingOfAbdominalPain	499131000000109
FindingOfAbdominalPain	499141000000100
FindingOfAbdominalPain	499161000000104
FindingOfAbdominalPain	499171000000106
FindingOfAbdominalPain	499181000000108
FindingOfAbdominalPain	499231000000101
FindingOfAbdominalPain	499241000000105
FindingOfAbdominalPain	499251000000108
FindingOfAbdominalPain	499281000000102
FindingOfAbdominalPain	502571000000104
FindingOfAbdominalPain	502581000000102
FindingOfAbdominalPain	502591000000100
FindingOfAbdominalPain	502841000000108
FindingOfAbdominalPain	540501000000100
FindingOfAbdominalPain	669941000000102
FindingOfAbdominalPain	791011000000108
FindingOfAbdominalPain	808731000000106

FindingOfAbdominalPain	958281000006114
FindingOfAbdominalPain	958291000006112
FindingOfAbdominalPain	958301000006113
FindingOfAbdominalPain	10760581000119104
FindingOfAbdominalPain	16580691000119107
FindingOfCough	7142008
FindingOfCough	11833005
FindingOfCough	17986004
FindingOfCough	19282004
FindingOfCough	20670007
FindingOfCough	28743005
FindingOfCough	43025008
FindingOfCough	46789001
FindingOfCough	46802002
FindingOfCough	49727002
FindingOfCough	52673003
FindingOfCough	59994004
FindingOfCough	62427007
FindingOfCough	62548007
FindingOfCough	62618004
FindingOfCough	62731002
FindingOfCough	63000007
FindingOfCough	68154008
FindingOfCough	82670009
FindingOfCough	102580004
FindingOfCough	111962006
FindingOfCough	123819004
FindingOfCough	135883003
FindingOfCough	154921000
FindingOfCough	161923004
FindingOfCough	161924005
FindingOfCough	161925006
FindingOfCough	161927003
FindingOfCough	161929000
FindingOfCough	161932002
FindingOfCough	161933007
FindingOfCough	161935000
FindingOfCough	161947006
FindingOfCough	191954008
FindingOfCough	192434000
FindingOfCough	207066005
FindingOfCough	225575005
FindingOfCough	225576006
FindingOfCough	247410004

FindingOfCough	248589007
FindingOfCough	248590003
FindingOfCough	248593001
FindingOfCough	248594007
FindingOfCough	268771003
FindingOfCough	271567008
FindingOfCough	272039006
FindingOfCough	276314008
FindingOfCough	277357006
FindingOfCough	284523002
FindingOfCough	289113002
FindingOfCough	289114008
FindingOfCough	289115009
FindingOfCough	289116005
FindingOfCough	289965001
FindingOfCough	300959008
FindingOfCough	301236000
FindingOfCough	301245004
FindingOfCough	301246003
FindingOfCough	301247007
FindingOfCough	306780002
FindingOfCough	306781003
FindingOfCough	306782005
FindingOfCough	315246003
FindingOfCough	366124008
FindingOfCough	366125009
FindingOfCough	366126005
FindingOfCough	409596002
FindingOfCough	417850002
FindingOfCough	445241004
FindingOfCough	769211009
FindingOfCough	200151000000106
FindingOfCough	216701000000107
FindingOfCough	216711000000109
FindingOfCough	287121000000103
FindingOfCough	287131000000101
FindingOfCough	498061000000106
FindingOfCough	536861000000102
FindingOfCough	677011000000103
FindingOfCough	709481000000105
FindingOfCough	810511000000109
FindingOfCough	831721000000106
FindingOfCough	1709331000006110
FindingOfCough	1763631000006115

FindingOfCough	1780331000006117
FindingOfDecreaseInAppetite	64379006
FindingOfDecreaseInAppetite	79890006
FindingOfDecreaseInAppetite	206915006
FindingOfDecreaseInAppetite	206916007
FindingOfDecreaseInAppetite	206917003
FindingOfDecreaseInAppetite	249468005
FindingOfDecreaseInAppetite	249469002
FindingOfDecreaseInAppetite	249470001
FindingOfDecreaseInAppetite	249471002
FindingOfDecreaseInAppetite	269813009
FindingOfDecreaseInAppetite	39161000000101
FindingOfDecreaseInAppetite	41421000000102
FindingOfDecreaseInAppetite	46641000000101
FindingOfDecreaseInAppetite	78901000000106
FindingOfDecreaseInAppetite	432541000000106
FindingOfDecreaseInAppetite	451151000000109
FindingOfDecreaseInAppetite	496871000000107
FindingOfDecreaseInAppetite	496881000000109
FindingOfDiarrhoea	2919008
FindingOfDiarrhoea	2946003
FindingOfDiarrhoea	11003002
FindingOfDiarrhoea	14384003
FindingOfDiarrhoea	15699003
FindingOfDiarrhoea	18168004
FindingOfDiarrhoea	18425006
FindingOfDiarrhoea	18805001
FindingOfDiarrhoea	24412005
FindingOfDiarrhoea	25898005
FindingOfDiarrhoea	31499008
FindingOfDiarrhoea	34167008
FindingOfDiarrhoea	38205001
FindingOfDiarrhoea	43240000
FindingOfDiarrhoea	49237006
FindingOfDiarrhoea	53156005
FindingOfDiarrhoea	62315008
FindingOfDiarrhoea	64679001
FindingOfDiarrhoea	69980003
FindingOfDiarrhoea	70506007
FindingOfDiarrhoea	79800001
FindingOfDiarrhoea	82047000
FindingOfDiarrhoea	83134002
FindingOfDiarrhoea	95544006
FindingOfDiarrhoea	95545007

FindingOfDiarrhoea	95879001
FindingOfDiarrhoea	128333008
FindingOfDiarrhoea	144842009
FindingOfDiarrhoea	162103003
FindingOfDiarrhoea	167616008
FindingOfDiarrhoea	187284001
FindingOfDiarrhoea	197125005
FindingOfDiarrhoea	197132001
FindingOfDiarrhoea	207145000
FindingOfDiarrhoea	235839006
FindingOfDiarrhoea	236074001
FindingOfDiarrhoea	236075000
FindingOfDiarrhoea	236077008
FindingOfDiarrhoea	236078003
FindingOfDiarrhoea	249517009
FindingOfDiarrhoea	249519007
FindingOfDiarrhoea	249619001
FindingOfDiarrhoea	267060006
FindingOfDiarrhoea	268651002
FindingOfDiarrhoea	268850004
FindingOfDiarrhoea	275297005
FindingOfDiarrhoea	276524004
FindingOfDiarrhoea	288199005
FindingOfDiarrhoea	301469002
FindingOfDiarrhoea	359658002
FindingOfDiarrhoea	398032003
FindingOfDiarrhoea	407373009
FindingOfDiarrhoea	409587002
FindingOfDiarrhoea	425739008
FindingOfDiarrhoea	428867008
FindingOfDiarrhoea	703406006
FindingOfDiarrhoea	721615009
FindingOfDiarrhoea	734884008
FindingOfDiarrhoea	468471000000105
FindingOfDiarrhoea	498651000000101
FindingOfDiarrhoea	685501000000109
FindingOfDiarrhoea	821491000000101
FindingOfDiarrhoea	1851601000006119
FindingOfDiarrhoea	11718971000119100
FindingOfDizziness	69096003
FindingOfDizziness	103017008
FindingOfDizziness	103018003
FindingOfDizziness	103293001
FindingOfDizziness	139526005

FindingOfDizziness	139529003
FindingOfDizziness	158146007
FindingOfDizziness	162257004
FindingOfDizziness	162260006
FindingOfDizziness	206739005
FindingOfDizziness	206740007
FindingOfDizziness	206742004
FindingOfDizziness	206745002
FindingOfDizziness	249986000
FindingOfDizziness	249987009
FindingOfDizziness	249988004
FindingOfDizziness	271789005
FindingOfDizziness	271790001
FindingOfDizziness	271791002
FindingOfDizziness	315018008
FindingOfDizziness	386705008
FindingOfDizziness	404640003
FindingOfDizziness	407645004
FindingOfDizziness	429530004
FindingOfDizziness	473188002
FindingOfDizziness	722950001
FindingOfDizziness	735548000
FindingOfDizziness	762244004
FindingOfDizziness	243241000000100
FindingOfDizziness	389501000000101
FindingOfDizziness	454071000000102
FindingOfDizziness	495591000000100
FindingOfDizziness	495601000000106
FindingOfDizziness	495621000000102
FindingOfDizziness	794101000000105
FindingOfDizziness	816651000000109
FindingOfDizziness	919621000000103
FindingOfDizziness	919631000000101
FindingOfDrowsiness	36124002
FindingOfDrowsiness	44455001
FindingOfDrowsiness	60380001
FindingOfDrowsiness	77692006
FindingOfDrowsiness	79280005
FindingOfDrowsiness	89415002
FindingOfDrowsiness	95631006
FindingOfDrowsiness	191999000
FindingOfDrowsiness	193042000
FindingOfDrowsiness	194439006
FindingOfDrowsiness	206711009

FindingOfDrowsiness	206712002
FindingOfDrowsiness	206751007
FindingOfDrowsiness	230488004
FindingOfDrowsiness	230489007
FindingOfDrowsiness	230490003
FindingOfDrowsiness	230491004
FindingOfDrowsiness	230492006
FindingOfDrowsiness	248261008
FindingOfDrowsiness	267088000
FindingOfDrowsiness	268653004
FindingOfDrowsiness	271102006
FindingOfDrowsiness	271782001
FindingOfDrowsiness	284091004
FindingOfDrowsiness	309732001
FindingOfDrowsiness	313432002
FindingOfDrowsiness	315243006
FindingOfDrowsiness	370971007
FindingOfDrowsiness	372947007
FindingOfDrowsiness	418424006
FindingOfDrowsiness	426451004
FindingOfDrowsiness	426943005
FindingOfDrowsiness	442292004
FindingOfDrowsiness	442416002
FindingOfDrowsiness	722293005
FindingOfDrowsiness	724749007
FindingOfDrowsiness	724750007
FindingOfDrowsiness	735676003
FindingOfDrowsiness	141000119100
FindingOfDrowsiness	3731000119107
FindingOfDrowsiness	31771000119102
FindingOfDrowsiness	91471000119102
FindingOfDrowsiness	91521000119104
FindingOfDrowsiness	135321000119107
FindingOfDrowsiness	424801000000100
FindingOfDrowsiness	495371000000106
FindingOfDrowsiness	495381000000108
FindingOfDrowsiness	502091000000108
FindingOfDrowsiness	502761000000100
FindingOfDrowsiness	654361000000105
FindingOfDrowsiness	671501000000102
FindingOfDrowsiness	837041000000105
FindingOfDrowsiness	857601000000103
FindingOfFatigue	51771007
FindingOfFatigue	52702003

FindingOfFatigue	73266005
FindingOfFatigue	84229001
FindingOfFatigue	88895004
FindingOfFatigue	158167007
FindingOfFatigue	158172003
FindingOfFatigue	158173008
FindingOfFatigue	161871003
FindingOfFatigue	199121007
FindingOfFatigue	199122000
FindingOfFatigue	199123005
FindingOfFatigue	199124004
FindingOfFatigue	206765009
FindingOfFatigue	206767001
FindingOfFatigue	206770002
FindingOfFatigue	206771003
FindingOfFatigue	206773000
FindingOfFatigue	224960004
FindingOfFatigue	248269005
FindingOfFatigue	267031002
FindingOfFatigue	267032009
FindingOfFatigue	271795006
FindingOfFatigue	272060000
FindingOfFatigue	272062008
FindingOfFatigue	314109004
FindingOfFatigue	420900006
FindingOfFatigue	442099003
FindingOfFatigue	444042007
FindingOfFatigue	704369007
FindingOfFatigue	713568000
FindingOfFatigue	716749005
FindingOfFatigue	784317004
FindingOfFatigue	784318009
FindingOfFatigue	97201000119101
FindingOfFatigue	377161000000108
FindingOfFatigue	377171000000101
FindingOfFatigue	377181000000104
FindingOfFatigue	414631000000100
FindingOfFatigue	463761000000103
FindingOfFatigue	480621000000108
FindingOfFatigue	491281000000101
FindingOfFatigue	491291000000104
FindingOfFatigue	495751000000103
FindingOfFatigue	495771000000107
FindingOfFatigue	495791000000106

FindingOfFatigue	495801000000105
FindingOfFatigue	508581000000104
FindingOfFatigue	580521000000101
FindingOfFatigue	698941000000107
FindingOfFeverWithChills	75125004
FindingOfFeverWithChills	158163006
FindingOfFeverWithChills	206760004
FindingOfFeverWithChills	274640006
FindingOfFeverWithChills	495731000000105
FindingOfHeadache	330007
FindingOfHeadache	4448006
FindingOfHeadache	4473006
FindingOfHeadache	4969004
FindingOfHeadache	13322008
FindingOfHeadache	23186000
FindingOfHeadache	25064002
FindingOfHeadache	26150009
FindingOfHeadache	37796009
FindingOfHeadache	38823002
FindingOfHeadache	41413006
FindingOfHeadache	43242008
FindingOfHeadache	44538002
FindingOfHeadache	49605003
FindingOfHeadache	54012000
FindingOfHeadache	56097005
FindingOfHeadache	59292006
FindingOfHeadache	66551002
FindingOfHeadache	79267007
FindingOfHeadache	83351003
FindingOfHeadache	86925001
FindingOfHeadache	95653008
FindingOfHeadache	95655001
FindingOfHeadache	95656000
FindingOfHeadache	95657009
FindingOfHeadache	95658004
FindingOfHeadache	95660002
FindingOfHeadache	103006007
FindingOfHeadache	103007003
FindingOfHeadache	103008008
FindingOfHeadache	103009000
FindingOfHeadache	103010005
FindingOfHeadache	103011009
FindingOfHeadache	103012002
FindingOfHeadache	112101004

FindingOfHeadache	128187005
FindingOfHeadache	129610005
FindingOfHeadache	139568005
FindingOfHeadache	139577003
FindingOfHeadache	154931007
FindingOfHeadache	162211001
FindingOfHeadache	162297001
FindingOfHeadache	162299003
FindingOfHeadache	162300006
FindingOfHeadache	162301005
FindingOfHeadache	162304002
FindingOfHeadache	162305001
FindingOfHeadache	162307009
FindingOfHeadache	162308004
FindingOfHeadache	162309007
FindingOfHeadache	162310002
FindingOfHeadache	162311003
FindingOfHeadache	169464001
FindingOfHeadache	192028000
FindingOfHeadache	193028008
FindingOfHeadache	193030005
FindingOfHeadache	193031009
FindingOfHeadache	193035000
FindingOfHeadache	193039006
FindingOfHeadache	194500004
FindingOfHeadache	194501000
FindingOfHeadache	198438009
FindingOfHeadache	206946005
FindingOfHeadache	230462002
FindingOfHeadache	230463007
FindingOfHeadache	230464001
FindingOfHeadache	230467008
FindingOfHeadache	230468003
FindingOfHeadache	230469006
FindingOfHeadache	230470007
FindingOfHeadache	230472004
FindingOfHeadache	230473009
FindingOfHeadache	230474003
FindingOfHeadache	230475002
FindingOfHeadache	230476001
FindingOfHeadache	230477005
FindingOfHeadache	230483008
FindingOfHeadache	267096005
FindingOfHeadache	271329006

FindingOfHeadache	279016001
FindingOfHeadache	279060001
FindingOfHeadache	301356002
FindingOfHeadache	301357006
FindingOfHeadache	364760007
FindingOfHeadache	395688005
FindingOfHeadache	398057008
FindingOfHeadache	398126006
FindingOfHeadache	423279000
FindingOfHeadache	423683008
FindingOfHeadache	423894005
FindingOfHeadache	424699007
FindingOfHeadache	425007008
FindingOfHeadache	425365009
FindingOfHeadache	425936006
FindingOfHeadache	427419006
FindingOfHeadache	443095000
FindingOfHeadache	445322004
FindingOfHeadache	445511002
FindingOfHeadache	698803006
FindingOfHeadache	699314009
FindingOfHeadache	699694000
FindingOfHeadache	703182002
FindingOfHeadache	711545001
FindingOfHeadache	712826000
FindingOfHeadache	712831003
FindingOfHeadache	722983008
FindingOfHeadache	724429004
FindingOfHeadache	725058003
FindingOfHeadache	735938006
FindingOfHeadache	762353009
FindingOfHeadache	762354003
FindingOfHeadache	1119294003
FindingOfHeadache	1119295002
FindingOfHeadache	121021000119105
FindingOfHeadache	122731000119104
FindingOfHeadache	122751000119105
FindingOfHeadache	124001000119104
FindingOfHeadache	124081000119107
FindingOfHeadache	124171000119105
FindingOfHeadache	145611000119107
FindingOfHeadache	203861000000109
FindingOfHeadache	211591000000104
FindingOfHeadache	248661000000105

FindingOfHeadache	294081000119102
FindingOfHeadache	294091000119104
FindingOfHeadache	338091000000104
FindingOfHeadache	400601000000103
FindingOfHeadache	418301000000106
FindingOfHeadache	419971000000109
FindingOfHeadache	429051000000106
FindingOfHeadache	443341000000106
FindingOfHeadache	444251000000103
FindingOfHeadache	466441000000100
FindingOfHeadache	467691000000105
FindingOfHeadache	497101000000107
FindingOfHeadache	509691000000108
FindingOfHeadache	585561000000103
FindingOfHeadache	603711000000104
FindingOfHeadache	603721000000105
FindingOfHeadache	781381000000100
FindingOfHeadache	807761000000102
FindingOfHeadache	808341000000101
FindingOfHeadache	808361000000100
FindingOfHeadache	808371000000107
FindingOfHeadache	808511000000105
FindingOfHeadache	1573091000006118
FindingOfHeadache	1708841000006115
FindingOfHeadache	1743431000006111
FindingOfJointPain	30989003
FindingOfJointPain	35678005
FindingOfJointPain	46960006
FindingOfJointPain	49218002
FindingOfJointPain	57676002
FindingOfJointPain	91943004
FindingOfJointPain	202472008
FindingOfJointPain	202478007
FindingOfJointPain	202479004
FindingOfJointPain	202480001
FindingOfJointPain	202481002
FindingOfJointPain	202482009
FindingOfJointPain	202483004
FindingOfJointPain	202484005
FindingOfJointPain	202485006
FindingOfJointPain	202487003
FindingOfJointPain	202489000
FindingOfJointPain	202490009
FindingOfJointPain	202491008

FindingOfJointPain	202493006
FindingOfJointPain	202495004
FindingOfJointPain	202496003
FindingOfJointPain	202497007
FindingOfJointPain	240271006
FindingOfJointPain	247369005
FindingOfJointPain	267949000
FindingOfJointPain	267950000
FindingOfJointPain	267951001
FindingOfJointPain	267952008
FindingOfJointPain	267953003
FindingOfJointPain	267954009
FindingOfJointPain	277138006
FindingOfJointPain	279063004
FindingOfJointPain	279066007
FindingOfJointPain	279067003
FindingOfJointPain	298253002
FindingOfJointPain	298254008
FindingOfJointPain	298255009
FindingOfJointPain	298376001
FindingOfJointPain	298478001
FindingOfJointPain	298579007
FindingOfJointPain	298674008
FindingOfJointPain	298857005
FindingOfJointPain	298858000
FindingOfJointPain	298929004
FindingOfJointPain	299018007
FindingOfJointPain	299112005
FindingOfJointPain	299199000
FindingOfJointPain	299308007
FindingOfJointPain	299377003
FindingOfJointPain	299447008
FindingOfJointPain	299513007
FindingOfJointPain	299554004
FindingOfJointPain	299633000
FindingOfJointPain	309765009
FindingOfJointPain	309767001
FindingOfJointPain	309768006
FindingOfJointPain	386207004
FindingOfJointPain	387638003
FindingOfJointPain	429531000
FindingOfJointPain	433017009
FindingOfJointPain	445422000
FindingOfJointPain	472957007

FindingOfJointPain	703619001
FindingOfJointPain	713413001
FindingOfJointPain	736428003
FindingOfJointPain	736447000
FindingOfJointPain	772826002
FindingOfJointPain	774136009
FindingOfJointPain	774137000
FindingOfJointPain	782661001
FindingOfJointPain	1003721002
FindingOfJointPain	87381000119101
FindingOfJointPain	112581000119104
FindingOfJointPain	298351000000107
FindingOfJointPain	629821000000108
FindingOfJointPain	629831000000105
FindingOfJointPain	687001000000108
FindingOfJointPain	781761000000108
FindingOfJointPain	876351000000106
FindingOfJointPain	921261000000101
FindingOfJointPain	921271000000108
FindingOfJointPain	937211000000107
FindingOfJointPain	1075631000119107
FindingOfJointPain	1075651000119101
FindingOfJointPain	1076721000119108
FindingOfJointPain	1076731000119106
FindingOfJointPain	1078021000000101
FindingOfJointPain	1089931000000101
FindingOfJointPain	1094851000000101
FindingOfJointPain	1892771000006118
FindingOfJointPain	12247571000119109
FindingOfJointPain	12400141000119103
FindingOfJointPain	12400181000119108
FindingOfJointPain	12400221000119100
FindingOfJointPain	12400261000119105
FindingOfJointPain	12400301000119102
FindingOfJointPain	12400341000119100
FindingOfJointPain	15632451000119102
FindingOfJointPain	15632491000119107
FindingOfJointPain	15632531000119107
FindingOfJointPain	15639561000119107
FindingOfJointPain	15639721000119105
FindingOfJointPain	16899701000119102
FindingOfMalaise	89565008
FindingOfMalaise	206765009
FindingOfMalaise	206766005

FindingOfMalaise	206773000
FindingOfMalaise	271795006
FindingOfMalaise	272036004
FindingOfMalaise	367391008
FindingOfMalaise	420801006
FindingOfMalaise	414631000000100
FindingOfMalaise	463761000000103
FindingOfMalaise	495751000000103
FindingOfMalaise	495761000000100
FindingOfMyalgia	49605003
FindingOfMyalgia	54981004
FindingOfMyalgia	65323003
FindingOfMyalgia	68962001
FindingOfMyalgia	76821002
FindingOfMyalgia	83264000
FindingOfMyalgia	95415006
FindingOfMyalgia	95416007
FindingOfMyalgia	95421005
FindingOfMyalgia	239938009
FindingOfMyalgia	240107001
FindingOfMyalgia	279030006
FindingOfMyalgia	279036000
FindingOfMyalgia	279041008
FindingOfMyalgia	279070004
FindingOfMyalgia	288225004
FindingOfMyalgia	288226003
FindingOfMyalgia	288227007
FindingOfMyalgia	288228002
FindingOfMyalgia	288229005
FindingOfMyalgia	288230000
FindingOfMyalgia	288231001
FindingOfMyalgia	288232008
FindingOfMyalgia	298292009
FindingOfMyalgia	403735006
FindingOfMyalgia	699682009
FindingOfMyalgia	702549002
FindingOfMyalgia	712752004
FindingOfMyalgia	726531007
FindingOfMyalgia	28221000119103
FindingOfMyalgia	41321000119101
FindingOfMyalgia	113611000119100
FindingOfMyalgia	186231000000109
FindingOfMyalgia	189671000000104
FindingOfMyalgia	675541000000101

FindingOfMyalgia	16462851000119106
FindingOfNausea	1488000
FindingOfNausea	2919008
FindingOfNausea	16932000
FindingOfNausea	18846006
FindingOfNausea	34591007
FindingOfNausea	64581007
FindingOfNausea	73335002
FindingOfNausea	73879007
FindingOfNausea	91173007
FindingOfNausea	139330007
FindingOfNausea	139332004
FindingOfNausea	139334003
FindingOfNausea	158421000
FindingOfNausea	162055004
FindingOfNausea	162057007
FindingOfNausea	162060000
FindingOfNausea	207110008
FindingOfNausea	207111007
FindingOfNausea	207114004
FindingOfNausea	207116002
FindingOfNausea	236084000
FindingOfNausea	272043005
FindingOfNausea	300576004
FindingOfNausea	419219000
FindingOfNausea	422587007
FindingOfNausea	698861005
FindingOfNausea	762279002
FindingOfNausea	400471000000109
FindingOfNausea	425721000000101
FindingOfNausea	498391000000103
FindingOfNausea	498401000000100
FindingOfNausea	498431000000106
FindingOfNausea	582741000000100
FindingOfNausea	812681000000102
FindingOfOedemaOfFace	19452008
FindingOfOedemaOfFace	49563000
FindingOfOedemaOfFace	89091004
FindingOfOedemaOfFace	238807000
FindingOfOedemaOfFace	402405007
FindingOfOedemaOfFace	402406008
FindingOfOedemaOfFace	403381009
FindingOfOedemaOfFace	403382002
FindingOfOedemaOfFace	403383007

FindingOfOedemaOfFace	445088006
FindingOfOedemaOfFace	700263000
FindingOfOedemaOfFace	700327003
FindingOfOedemaOfFace	700337008
FindingOfOedemaOfFace	700339006
FindingOfOedemaOfFace	789007001
FindingOfOedemaOfFace	846577007
FindingOfOedemaOfFace	846579005
FindingOfOedemaOfFace	342461000119105
FindingOfOedemaOfFace	342471000119104
FindingOfOedemaOfFace	342481000119101
FindingOfOedemaOfFace	342491000119103
FindingOfOedemaOfFace	342501000119105
FindingOfOedemaOfFace	342511000119108
FindingOfOedemaOfFace	348101000119102
FindingOfOedemaOfFace	348551000119107
FindingOfOedemaOfFace	349131000119103
FindingOfOedemaOfFace	15732321000119108
FindingOfOedemaOfFace	15930101000119109
FindingOfOedemaOfFace	15930141000119106
FindingOfParaesthesia	1141007
FindingOfParaesthesia	57105000
FindingOfParaesthesia	62507009
FindingOfParaesthesia	79256006
FindingOfParaesthesia	85007004
FindingOfParaesthesia	91019004
FindingOfParaesthesia	95358009
FindingOfParaesthesia	95524000
FindingOfParaesthesia	95527007
FindingOfParaesthesia	95665007
FindingOfParaesthesia	95673003
FindingOfParaesthesia	95676006
FindingOfParaesthesia	102602003
FindingOfParaesthesia	135873002
FindingOfParaesthesia	155076004
FindingOfParaesthesia	206857001
FindingOfParaesthesia	309086004
FindingOfParaesthesia	309087008
FindingOfParaesthesia	310481001
FindingOfParaesthesia	403600002
FindingOfParaesthesia	429783005
FindingOfParaesthesia	762374005
FindingOfParaesthesia	786837007
FindingOfParaesthesia	840319006

FindingOfParaesthesia	101000119102
FindingOfParaesthesia	496421000000105
FindingOfParaesthesia	600691000000108
FindingOfParaesthesia	15634791000119106
FindingOfParaesthesia	15634841000119109
FindingOfParaesthesia	15973661000119106
FindingOfParaesthesia	15973701000119104
FindingOfPeripheralTremor	18908003
FindingOfPeripheralTremor	25082004
FindingOfPeripheralTremor	26079004
FindingOfPeripheralTremor	27369003
FindingOfPeripheralTremor	30721006
FindingOfPeripheralTremor	32838008
FindingOfPeripheralTremor	36637003
FindingOfPeripheralTremor	36730005
FindingOfPeripheralTremor	40290000
FindingOfPeripheralTremor	41713005
FindingOfPeripheralTremor	41846009
FindingOfPeripheralTremor	42800007
FindingOfPeripheralTremor	45233003
FindingOfPeripheralTremor	49718003
FindingOfPeripheralTremor	52887000
FindingOfPeripheralTremor	56610005
FindingOfPeripheralTremor	66880003
FindingOfPeripheralTremor	70765006
FindingOfPeripheralTremor	74178009
FindingOfPeripheralTremor	78261002
FindingOfPeripheralTremor	112109002
FindingOfPeripheralTremor	206806000
FindingOfPeripheralTremor	230339003
FindingOfPeripheralTremor	230340001
FindingOfPeripheralTremor	230341002
FindingOfPeripheralTremor	267079009
FindingOfPeripheralTremor	274239004
FindingOfPeripheralTremor	277358001
FindingOfPeripheralTremor	302004004
FindingOfPeripheralTremor	313241005
FindingOfPeripheralTremor	422383007
FindingOfPeripheralTremor	425763000
FindingOfPeripheralTremor	430690005
FindingOfPeripheralTremor	443853005
FindingOfPeripheralTremor	445407005
FindingOfPeripheralTremor	715902009
FindingOfPeripheralTremor	722969006

FindingOfPeripheralTremor	722970007
FindingOfPeripheralTremor	722971006
FindingOfPeripheralTremor	724771002
FindingOfPeripheralTremor	788912006
FindingOfPeripheralTremor	829991004
FindingOfPeripheralTremor	835278006
FindingOfPeripheralTremor	87881000119106
FindingOfPeripheralTremor	350561000119109
FindingOfPeripheralTremor	427441000000108
FindingOfPeripheralTremor	472121000000107
FindingOfPeripheralTremor	502231000000103
FindingOfPeripheralTremor	502891000000103
FindingOfPeripheralTremor	1916861000006114
FindingOfSoreThroat	41582007
FindingOfSoreThroat	43878008
FindingOfSoreThroat	76651006
FindingOfSoreThroat	111816002
FindingOfSoreThroat	139658009
FindingOfSoreThroat	154301006
FindingOfSoreThroat	154304003
FindingOfSoreThroat	162388002
FindingOfSoreThroat	162397003
FindingOfSoreThroat	186357007
FindingOfSoreThroat	186362008
FindingOfSoreThroat	195659006
FindingOfSoreThroat	195672007
FindingOfSoreThroat	206949003
FindingOfSoreThroat	267102003
FindingOfSoreThroat	275488008
FindingOfSoreThroat	497121000000103
FindingOfSoreThroat	538321000000103
FindingOfSoreThroat	538331000000101
FindingOfSoreThroat	542861000000102
FindingOfSoreThroat	10629231000119109
FindingOfTinnitus	28715001
FindingOfTinnitus	60862001
FindingOfTinnitus	62452009
FindingOfTinnitus	95822008
FindingOfTinnitus	95823003
FindingOfTinnitus	95824009
FindingOfTinnitus	95825005
FindingOfTinnitus	95826006
FindingOfTinnitus	232319009
FindingOfTinnitus	232320003

FindingOfTinnitus	232321004
FindingOfTinnitus	232322006
FindingOfTinnitus	232323001
FindingOfTinnitus	866003005
FindingOfTinnitus	4831000119102
FindingOfTinnitus	117411000119106
FindingOfTinnitus	117421000119104
FindingOfTinnitus	643771000000102
FindingOfTinnitus	1084191000119105
FindingOfTinnitus	1084201000119108
FindingOfTinnitus	1084621000119102
FindingOfTinnitus	1084631000119104
FindingOfTinnitus	1089261000119105
FindingOfTinnitus	1089271000119104
FindingOfTinnitus	1089621000119103
FindingOfTinnitus	1089631000119100
FindingOfTinnitus	1091871000119103
FindingOfTinnitus	1091881000119100
FindingOfTinnitus	1092231000119108
FindingOfTinnitus	1092241000119104
FindingOfVomiting	1488000
FindingOfVomiting	2919008
FindingOfVomiting	3094009
FindingOfVomiting	8579004
FindingOfVomiting	9814003
FindingOfVomiting	11767005
FindingOfVomiting	15387003
FindingOfVomiting	16932000
FindingOfVomiting	18773000
FindingOfVomiting	18846006
FindingOfVomiting	23971007
FindingOfVomiting	34591007
FindingOfVomiting	34923007
FindingOfVomiting	37224001
FindingOfVomiting	38685005
FindingOfVomiting	45941008
FindingOfVomiting	45994004
FindingOfVomiting	48000002
FindingOfVomiting	49206006
FindingOfVomiting	71419002
FindingOfVomiting	72245005
FindingOfVomiting	73335002
FindingOfVomiting	84480002
FindingOfVomiting	91173007

FindingOfVomiting	162065005
FindingOfVomiting	191970005
FindingOfVomiting	192450008
FindingOfVomiting	196746003
FindingOfVomiting	197130009
FindingOfVomiting	207110008
FindingOfVomiting	207112000
FindingOfVomiting	207113005
FindingOfVomiting	207114004
FindingOfVomiting	207116002
FindingOfVomiting	225586007
FindingOfVomiting	236062000
FindingOfVomiting	236083006
FindingOfVomiting	236084000
FindingOfVomiting	249497008
FindingOfVomiting	249500002
FindingOfVomiting	249519007
FindingOfVomiting	272044004
FindingOfVomiting	275297005
FindingOfVomiting	288199005
FindingOfVomiting	300359004
FindingOfVomiting	301790009
FindingOfVomiting	308151006
FindingOfVomiting	332982000
FindingOfVomiting	370402009
FindingOfVomiting	416604007
FindingOfVomiting	419219000
FindingOfVomiting	422400008
FindingOfVomiting	424580008
FindingOfVomiting	444673007
FindingOfVomiting	698861005
FindingOfVomiting	722933003
FindingOfVomiting	723975009
FindingOfVomiting	765480005
FindingOfVomiting	134021000119105
FindingOfVomiting	146291000119108
FindingOfVomiting	294001000119105
FindingOfVomiting	400471000000109
FindingOfVomiting	425721000000101
FindingOfVomiting	498391000000103
FindingOfVomiting	498411000000103
FindingOfVomiting	498421000000109
FindingOfVomiting	498431000000106
FindingOfVomiting	502651000000107

FindingOfVomiting	582751000000102
FindingOfVomiting	607791000000102
FindingOfVomiting	607801000000103
FindingOfVomiting	665931000000108
FindingOfVomiting	1851581000006112
FindingOfVomiting	1851591000006110
FindingOfVomiting	1959511000006111
FindingOfWeakness	13791008
FindingOfWeakness	18726006
FindingOfWeakness	41786007
FindingOfWeakness	126013009
FindingOfWeakness	161873000
FindingOfWeakness	161874006
FindingOfWeakness	206768006
FindingOfWeakness	207529006
FindingOfWeakness	207530001
FindingOfWeakness	248278004
FindingOfWeakness	250002000
FindingOfWeakness	271556007
FindingOfWeakness	271875007
FindingOfWeakness	272036004
FindingOfWeakness	274236006
FindingOfWeakness	373931001
FindingOfWeakness	439201000000104
FindingOfWeakness	501681000000107
FindingOfWeakness	501691000000109
FindingOfWeakness	502161000000101
FindingOfWeakness	502201000000109
FindingOfWeakness	15634971000119107
GuillainBarrieSyndrome	1767005
GuillainBarrieSyndrome	40956001
GuillainBarrieSyndrome	193175006
GuillainBarrieSyndrome	230548007
GuillainBarrieSyndrome	715770009
GuillainBarrieSyndrome	716722005
GuillainBarrieSyndrome	716723000
GuillainBarrieSyndrome	766049000
GuillainBarrieSyndrome	766722008
GuillainBarrieSyndrome	783010003
GuillainBarrieSyndrome	783244002
Hyperhidrosis	1170003
Hyperhidrosis	45294007
Hyperhidrosis	206774006
Hyperhidrosis	206777004

Hyperhidrosis	206778009
Hyperhidrosis	206779001
Hyperhidrosis	230660000
Hyperhidrosis	230668007
Hyperhidrosis	238758008
Hyperhidrosis	274673004
Hyperhidrosis	274674005
Hyperhidrosis	303089000
Hyperhidrosis	303090009
Hyperhidrosis	312230002
Hyperhidrosis	403374002
Hyperhidrosis	403375001
Hyperhidrosis	403376000
Hyperhidrosis	403377009
Hyperhidrosis	403378004
Hyperhidrosis	403379007
Hyperhidrosis	403380005
Hyperhidrosis	422194002
Hyperhidrosis	427794001
Hyperhidrosis	723000001
Hyperhidrosis	782457003
Hyperhidrosis	782458008
Hyperhidrosis	782459000
Hyperhidrosis	782461009
Hyperhidrosis	782462002
Hyperhidrosis	782463007
Hyperhidrosis	783550006
Hyperhidrosis	860880003
Hyperhidrosis	92661000119104
Hyperhidrosis	397571000000102
Hyperhidrosis	400741000000108
Hyperhidrosis	495811000000107
Hyperhidrosis	495841000000108
Hyperhidrosis	495851000000106
Hyperhidrosis	778351000000107
Hyperhidrosis	10677551000119108
Hyperhidrosis	10677591000119103
Hyperhidrosis	10677631000119103
Hyperhidrosis	10677671000119100
Hyperhidrosis	14070001000004105
Hyperhidrosis	16026591000119103
Hyperhidrosis	16026631000119103
HypersensitivityReaction	228007
HypersensitivityReaction	490008

HypersensitivityReaction	1945002
HypersensitivityReaction	7114006
HypersensitivityReaction	8641003
HypersensitivityReaction	9768003
HypersensitivityReaction	10785007
HypersensitivityReaction	10803007
HypersensitivityReaction	11354005
HypersensitivityReaction	11944003
HypersensitivityReaction	12088005
HypersensitivityReaction	12263007
HypersensitivityReaction	13394002
HypersensitivityReaction	14589007
HypersensitivityReaction	14654002
HypersensitivityReaction	15911003
HypersensitivityReaction	16341002
HypersensitivityReaction	16623004
HypersensitivityReaction	17365008
HypersensitivityReaction	18690003
HypersensitivityReaction	19076009
HypersensitivityReaction	19274004
HypersensitivityReaction	21626009
HypersensitivityReaction	21957007
HypersensitivityReaction	23315001
HypersensitivityReaction	25868003
HypersensitivityReaction	25897000
HypersensitivityReaction	26006005
HypersensitivityReaction	28031001
HypersensitivityReaction	29268000
HypersensitivityReaction	30352005
HypersensitivityReaction	35001004
HypersensitivityReaction	37471005
HypersensitivityReaction	37981002
HypersensitivityReaction	38729007
HypersensitivityReaction	39579001
HypersensitivityReaction	41156006
HypersensitivityReaction	41270004
HypersensitivityReaction	48347002
HypersensitivityReaction	51338006
HypersensitivityReaction	52333004
HypersensitivityReaction	53954009
HypersensitivityReaction	55985003
HypersensitivityReaction	56968009
HypersensitivityReaction	59940009
HypersensitivityReaction	63088003

HypersensitivityReaction	63924002
HypersensitivityReaction	67242002
HypersensitivityReaction	67375008
HypersensitivityReaction	68270003
HypersensitivityReaction	68333005
HypersensitivityReaction	69339004
HypersensitivityReaction	73448002
HypersensitivityReaction	74069000
HypersensitivityReaction	74455006
HypersensitivityReaction	79337003
HypersensitivityReaction	79958002
HypersensitivityReaction	81710009
HypersensitivityReaction	83699005
HypersensitivityReaction	84234002
HypersensitivityReaction	85407005
HypersensitivityReaction	86638007
HypersensitivityReaction	88899005
HypersensitivityReaction	89030000
HypersensitivityReaction	89099002
HypersensitivityReaction	90092004
HypersensitivityReaction	91232002
HypersensitivityReaction	91340006
HypersensitivityReaction	91930004
HypersensitivityReaction	91931000
HypersensitivityReaction	91932007
HypersensitivityReaction	91933002
HypersensitivityReaction	91934008
HypersensitivityReaction	91935009
HypersensitivityReaction	91936005
HypersensitivityReaction	91937001
HypersensitivityReaction	91938006
HypersensitivityReaction	91939003
HypersensitivityReaction	91940001
HypersensitivityReaction	91941002
HypersensitivityReaction	105978008
HypersensitivityReaction	106190000
HypersensitivityReaction	109258002
HypersensitivityReaction	109259005
HypersensitivityReaction	109260000
HypersensitivityReaction	109261001
HypersensitivityReaction	109775009
HypersensitivityReaction	111737003
HypersensitivityReaction	115666004
HypersensitivityReaction	123583002

HypersensitivityReaction	131147004
HypersensitivityReaction	135887002
HypersensitivityReaction	142165001
HypersensitivityReaction	161590003
HypersensitivityReaction	161591004
HypersensitivityReaction	161593001
HypersensitivityReaction	161594007
HypersensitivityReaction	161595008
HypersensitivityReaction	161596009
HypersensitivityReaction	161597000
HypersensitivityReaction	161598005
HypersensitivityReaction	161599002
HypersensitivityReaction	161600004
HypersensitivityReaction	161601000
HypersensitivityReaction	161602007
HypersensitivityReaction	161603002
HypersensitivityReaction	161604008
HypersensitivityReaction	161605009
HypersensitivityReaction	161606005
HypersensitivityReaction	161607001
HypersensitivityReaction	161608006
HypersensitivityReaction	161610008
HypersensitivityReaction	161612000
HypersensitivityReaction	161613005
HypersensitivityReaction	164993005
HypersensitivityReaction	164998001
HypersensitivityReaction	164999009
HypersensitivityReaction	165000009
HypersensitivityReaction	165001008
HypersensitivityReaction	188336009
HypersensitivityReaction	195663004
HypersensitivityReaction	195989002
HypersensitivityReaction	195990006
HypersensitivityReaction	212999007
HypersensitivityReaction	213020009
HypersensitivityReaction	213024000
HypersensitivityReaction	213320003
HypersensitivityReaction	232346004
HypersensitivityReaction	232347008
HypersensitivityReaction	232348003
HypersensitivityReaction	232349006
HypersensitivityReaction	232350006
HypersensitivityReaction	232430006
HypersensitivityReaction	233681001

HypersensitivityReaction	233682008
HypersensitivityReaction	233686006
HypersensitivityReaction	233687002
HypersensitivityReaction	233694004
HypersensitivityReaction	233695003
HypersensitivityReaction	233696002
HypersensitivityReaction	233697006
HypersensitivityReaction	233698001
HypersensitivityReaction	233699009
HypersensitivityReaction	233700005
HypersensitivityReaction	233701009
HypersensitivityReaction	233702002
HypersensitivityReaction	233774000
HypersensitivityReaction	236061007
HypersensitivityReaction	236540009
HypersensitivityReaction	238428001
HypersensitivityReaction	238429009
HypersensitivityReaction	238505000
HypersensitivityReaction	240407009
HypersensitivityReaction	240408004
HypersensitivityReaction	240409007
HypersensitivityReaction	240410002
HypersensitivityReaction	240411003
HypersensitivityReaction	240412005
HypersensitivityReaction	241929008
HypersensitivityReaction	241930003
HypersensitivityReaction	241931004
HypersensitivityReaction	241932006
HypersensitivityReaction	241933001
HypersensitivityReaction	241934007
HypersensitivityReaction	241935008
HypersensitivityReaction	241936009
HypersensitivityReaction	241937000
HypersensitivityReaction	241938005
HypersensitivityReaction	241939002
HypersensitivityReaction	241940000
HypersensitivityReaction	241941001
HypersensitivityReaction	241942008
HypersensitivityReaction	241943003
HypersensitivityReaction	241944009
HypersensitivityReaction	241945005
HypersensitivityReaction	241947002
HypersensitivityReaction	241948007
HypersensitivityReaction	241949004

HypersensitivityReaction	241951000
HypersensitivityReaction	241952007
HypersensitivityReaction	241953002
HypersensitivityReaction	241954008
HypersensitivityReaction	252097006
HypersensitivityReaction	266451002
HypersensitivityReaction	266931007
HypersensitivityReaction	266932000
HypersensitivityReaction	269284003
HypersensitivityReaction	274211000
HypersensitivityReaction	282092005
HypersensitivityReaction	282093000
HypersensitivityReaction	282094006
HypersensitivityReaction	282095007
HypersensitivityReaction	292068000
HypersensitivityReaction	293580007
HypersensitivityReaction	293582004
HypersensitivityReaction	293583009
HypersensitivityReaction	293584003
HypersensitivityReaction	293585002
HypersensitivityReaction	293586001
HypersensitivityReaction	293587005
HypersensitivityReaction	293588000
HypersensitivityReaction	293589008
HypersensitivityReaction	293590004
HypersensitivityReaction	293591000
HypersensitivityReaction	293592007
HypersensitivityReaction	293593002
HypersensitivityReaction	293594008
HypersensitivityReaction	293595009
HypersensitivityReaction	293596005
HypersensitivityReaction	293597001
HypersensitivityReaction	293598006
HypersensitivityReaction	293599003
HypersensitivityReaction	293600000
HypersensitivityReaction	293601001
HypersensitivityReaction	293602008
HypersensitivityReaction	293603003
HypersensitivityReaction	293604009
HypersensitivityReaction	293605005
HypersensitivityReaction	293606006
HypersensitivityReaction	293607002
HypersensitivityReaction	293608007
HypersensitivityReaction	293609004

HypersensitivityReaction	293610009
HypersensitivityReaction	293611008
HypersensitivityReaction	293612001
HypersensitivityReaction	293613006
HypersensitivityReaction	293614000
HypersensitivityReaction	293615004
HypersensitivityReaction	293616003
HypersensitivityReaction	293617007
HypersensitivityReaction	293618002
HypersensitivityReaction	293619005
HypersensitivityReaction	293620004
HypersensitivityReaction	293621000
HypersensitivityReaction	293622007
HypersensitivityReaction	293623002
HypersensitivityReaction	293624008
HypersensitivityReaction	293625009
HypersensitivityReaction	293626005
HypersensitivityReaction	293627001
HypersensitivityReaction	293628006
HypersensitivityReaction	293629003
HypersensitivityReaction	293630008
HypersensitivityReaction	293631007
HypersensitivityReaction	293632000
HypersensitivityReaction	293633005
HypersensitivityReaction	293634004
HypersensitivityReaction	293635003
HypersensitivityReaction	293636002
HypersensitivityReaction	293637006
HypersensitivityReaction	293638001
HypersensitivityReaction	293639009
HypersensitivityReaction	293640006
HypersensitivityReaction	293641005
HypersensitivityReaction	293643008
HypersensitivityReaction	293645001
HypersensitivityReaction	293646000
HypersensitivityReaction	293647009
HypersensitivityReaction	293648004
HypersensitivityReaction	293649007
HypersensitivityReaction	293650007
HypersensitivityReaction	293651006
HypersensitivityReaction	293652004
HypersensitivityReaction	293653009
HypersensitivityReaction	293654003
HypersensitivityReaction	293655002

HypersensitivityReaction	293656001
HypersensitivityReaction	293657005
HypersensitivityReaction	293658000
HypersensitivityReaction	293659008
HypersensitivityReaction	293660003
HypersensitivityReaction	293661004
HypersensitivityReaction	293662006
HypersensitivityReaction	293663001
HypersensitivityReaction	293664007
HypersensitivityReaction	293665008
HypersensitivityReaction	293666009
HypersensitivityReaction	293668005
HypersensitivityReaction	293669002
HypersensitivityReaction	293670001
HypersensitivityReaction	293671002
HypersensitivityReaction	293672009
HypersensitivityReaction	293673004
HypersensitivityReaction	293674005
HypersensitivityReaction	293675006
HypersensitivityReaction	293676007
HypersensitivityReaction	293678008
HypersensitivityReaction	293679000
HypersensitivityReaction	293680002
HypersensitivityReaction	293681003
HypersensitivityReaction	293682005
HypersensitivityReaction	293684006
HypersensitivityReaction	293685007
HypersensitivityReaction	293686008
HypersensitivityReaction	293687004
HypersensitivityReaction	293688009
HypersensitivityReaction	293689001
HypersensitivityReaction	293690005
HypersensitivityReaction	293691009
HypersensitivityReaction	293692002
HypersensitivityReaction	293693007
HypersensitivityReaction	293694001
HypersensitivityReaction	293695000
HypersensitivityReaction	293696004
HypersensitivityReaction	293697008
HypersensitivityReaction	293698003
HypersensitivityReaction	293699006
HypersensitivityReaction	293700007
HypersensitivityReaction	293701006
HypersensitivityReaction	293702004

HypersensitivityReaction	293703009
HypersensitivityReaction	293704003
HypersensitivityReaction	293705002
HypersensitivityReaction	293706001
HypersensitivityReaction	293707005
HypersensitivityReaction	293708000
HypersensitivityReaction	293709008
HypersensitivityReaction	293710003
HypersensitivityReaction	293711004
HypersensitivityReaction	293712006
HypersensitivityReaction	293713001
HypersensitivityReaction	293714007
HypersensitivityReaction	293715008
HypersensitivityReaction	293716009
HypersensitivityReaction	293717000
HypersensitivityReaction	293718005
HypersensitivityReaction	293719002
HypersensitivityReaction	293720008
HypersensitivityReaction	293721007
HypersensitivityReaction	293722000
HypersensitivityReaction	293723005
HypersensitivityReaction	293724004
HypersensitivityReaction	293725003
HypersensitivityReaction	293726002
HypersensitivityReaction	293727006
HypersensitivityReaction	293728001
HypersensitivityReaction	293732007
HypersensitivityReaction	293733002
HypersensitivityReaction	293735009
HypersensitivityReaction	293736005
HypersensitivityReaction	293737001
HypersensitivityReaction	293738006
HypersensitivityReaction	293739003
HypersensitivityReaction	293740001
HypersensitivityReaction	293741002
HypersensitivityReaction	293742009
HypersensitivityReaction	293743004
HypersensitivityReaction	293745006
HypersensitivityReaction	293746007
HypersensitivityReaction	293747003
HypersensitivityReaction	293748008
HypersensitivityReaction	293749000
HypersensitivityReaction	293750000
HypersensitivityReaction	293751001

HypersensitivityReaction	293752008
HypersensitivityReaction	293753003
HypersensitivityReaction	293754009
HypersensitivityReaction	293755005
HypersensitivityReaction	293756006
HypersensitivityReaction	293757002
HypersensitivityReaction	293758007
HypersensitivityReaction	293759004
HypersensitivityReaction	293760009
HypersensitivityReaction	293761008
HypersensitivityReaction	293762001
HypersensitivityReaction	293763006
HypersensitivityReaction	293764000
HypersensitivityReaction	293765004
HypersensitivityReaction	293766003
HypersensitivityReaction	293767007
HypersensitivityReaction	293768002
HypersensitivityReaction	293769005
HypersensitivityReaction	293770006
HypersensitivityReaction	293771005
HypersensitivityReaction	293772003
HypersensitivityReaction	293773008
HypersensitivityReaction	293774002
HypersensitivityReaction	293775001
HypersensitivityReaction	293776000
HypersensitivityReaction	293777009
HypersensitivityReaction	293778004
HypersensitivityReaction	293779007
HypersensitivityReaction	293780005
HypersensitivityReaction	293781009
HypersensitivityReaction	293782002
HypersensitivityReaction	293783007
HypersensitivityReaction	293784001
HypersensitivityReaction	293785000
HypersensitivityReaction	293786004
HypersensitivityReaction	293787008
HypersensitivityReaction	293788003
HypersensitivityReaction	293789006
HypersensitivityReaction	293790002
HypersensitivityReaction	293791003
HypersensitivityReaction	293792005
HypersensitivityReaction	293793000
HypersensitivityReaction	293794006
HypersensitivityReaction	293795007

HypersensitivityReaction	293796008
HypersensitivityReaction	293798009
HypersensitivityReaction	293799001
HypersensitivityReaction	293801003
HypersensitivityReaction	293802005
HypersensitivityReaction	293803000
HypersensitivityReaction	293804006
HypersensitivityReaction	293805007
HypersensitivityReaction	293806008
HypersensitivityReaction	293808009
HypersensitivityReaction	293809001
HypersensitivityReaction	293810006
HypersensitivityReaction	293811005
HypersensitivityReaction	293812003
HypersensitivityReaction	293813008
HypersensitivityReaction	293814002
HypersensitivityReaction	293815001
HypersensitivityReaction	293817009
HypersensitivityReaction	293818004
HypersensitivityReaction	293819007
HypersensitivityReaction	293822009
HypersensitivityReaction	293823004
HypersensitivityReaction	293824005
HypersensitivityReaction	293825006
HypersensitivityReaction	293826007
HypersensitivityReaction	293827003
HypersensitivityReaction	293828008
HypersensitivityReaction	293829000
HypersensitivityReaction	293830005
HypersensitivityReaction	293831009
HypersensitivityReaction	293832002
HypersensitivityReaction	293833007
HypersensitivityReaction	293834001
HypersensitivityReaction	293835000
HypersensitivityReaction	293836004
HypersensitivityReaction	293837008
HypersensitivityReaction	293838003
HypersensitivityReaction	293839006
HypersensitivityReaction	293840008
HypersensitivityReaction	293842000
HypersensitivityReaction	293843005
HypersensitivityReaction	293844004
HypersensitivityReaction	293845003
HypersensitivityReaction	293847006

HypersensitivityReaction	293848001
HypersensitivityReaction	293849009
HypersensitivityReaction	293850009
HypersensitivityReaction	293851008
HypersensitivityReaction	293853006
HypersensitivityReaction	293854000
HypersensitivityReaction	293855004
HypersensitivityReaction	293856003
HypersensitivityReaction	293857007
HypersensitivityReaction	293858002
HypersensitivityReaction	293859005
HypersensitivityReaction	293860000
HypersensitivityReaction	293861001
HypersensitivityReaction	293862008
HypersensitivityReaction	293863003
HypersensitivityReaction	293864009
HypersensitivityReaction	293865005
HypersensitivityReaction	293866006
HypersensitivityReaction	293867002
HypersensitivityReaction	293868007
HypersensitivityReaction	293869004
HypersensitivityReaction	293870003
HypersensitivityReaction	293871004
HypersensitivityReaction	293874007
HypersensitivityReaction	293875008
HypersensitivityReaction	293876009
HypersensitivityReaction	293877000
HypersensitivityReaction	293878005
HypersensitivityReaction	293879002
HypersensitivityReaction	293880004
HypersensitivityReaction	293881000
HypersensitivityReaction	293882007
HypersensitivityReaction	293883002
HypersensitivityReaction	293884008
HypersensitivityReaction	293885009
HypersensitivityReaction	293886005
HypersensitivityReaction	293887001
HypersensitivityReaction	293888006
HypersensitivityReaction	293889003
HypersensitivityReaction	293890007
HypersensitivityReaction	293891006
HypersensitivityReaction	293892004
HypersensitivityReaction	293893009
HypersensitivityReaction	293894003

HypersensitivityReaction	293895002
HypersensitivityReaction	293896001
HypersensitivityReaction	293897005
HypersensitivityReaction	293898000
HypersensitivityReaction	293899008
HypersensitivityReaction	293900003
HypersensitivityReaction	293901004
HypersensitivityReaction	293902006
HypersensitivityReaction	293903001
HypersensitivityReaction	293904007
HypersensitivityReaction	293905008
HypersensitivityReaction	293906009
HypersensitivityReaction	293907000
HypersensitivityReaction	293908005
HypersensitivityReaction	293909002
HypersensitivityReaction	293910007
HypersensitivityReaction	293911006
HypersensitivityReaction	293912004
HypersensitivityReaction	293913009
HypersensitivityReaction	293914003
HypersensitivityReaction	293915002
HypersensitivityReaction	293916001
HypersensitivityReaction	293917005
HypersensitivityReaction	293918000
HypersensitivityReaction	293919008
HypersensitivityReaction	293920002
HypersensitivityReaction	293921003
HypersensitivityReaction	293922005
HypersensitivityReaction	293923000
HypersensitivityReaction	293924006
HypersensitivityReaction	293925007
HypersensitivityReaction	293926008
HypersensitivityReaction	293927004
HypersensitivityReaction	293928009
HypersensitivityReaction	293929001
HypersensitivityReaction	293930006
HypersensitivityReaction	293931005
HypersensitivityReaction	293932003
HypersensitivityReaction	293933008
HypersensitivityReaction	293934002
HypersensitivityReaction	293935001
HypersensitivityReaction	293936000
HypersensitivityReaction	293937009
HypersensitivityReaction	293938004

HypersensitivityReaction	293939007
HypersensitivityReaction	293940009
HypersensitivityReaction	293941008
HypersensitivityReaction	293942001
HypersensitivityReaction	293943006
HypersensitivityReaction	293944000
HypersensitivityReaction	293945004
HypersensitivityReaction	293946003
HypersensitivityReaction	293948002
HypersensitivityReaction	293949005
HypersensitivityReaction	293950005
HypersensitivityReaction	293952002
HypersensitivityReaction	293953007
HypersensitivityReaction	293954001
HypersensitivityReaction	293955000
HypersensitivityReaction	293956004
HypersensitivityReaction	293957008
HypersensitivityReaction	293958003
HypersensitivityReaction	293959006
HypersensitivityReaction	293960001
HypersensitivityReaction	293962009
HypersensitivityReaction	293963004
HypersensitivityReaction	293964005
HypersensitivityReaction	293965006
HypersensitivityReaction	293966007
HypersensitivityReaction	293967003
HypersensitivityReaction	293968008
HypersensitivityReaction	293969000
HypersensitivityReaction	293970004
HypersensitivityReaction	293971000
HypersensitivityReaction	293972007
HypersensitivityReaction	293973002
HypersensitivityReaction	293974008
HypersensitivityReaction	293975009
HypersensitivityReaction	293976005
HypersensitivityReaction	293977001
HypersensitivityReaction	293978006
HypersensitivityReaction	293979003
HypersensitivityReaction	293980000
HypersensitivityReaction	293981001
HypersensitivityReaction	293982008
HypersensitivityReaction	293983003
HypersensitivityReaction	293984009
HypersensitivityReaction	293985005

HypersensitivityReaction	293986006
HypersensitivityReaction	293987002
HypersensitivityReaction	293988007
HypersensitivityReaction	293989004
HypersensitivityReaction	293990008
HypersensitivityReaction	293991007
HypersensitivityReaction	293992000
HypersensitivityReaction	293993005
HypersensitivityReaction	293994004
HypersensitivityReaction	293995003
HypersensitivityReaction	293996002
HypersensitivityReaction	293997006
HypersensitivityReaction	293998001
HypersensitivityReaction	293999009
HypersensitivityReaction	294000006
HypersensitivityReaction	294001005
HypersensitivityReaction	294002003
HypersensitivityReaction	294003008
HypersensitivityReaction	294004002
HypersensitivityReaction	294005001
HypersensitivityReaction	294006000
HypersensitivityReaction	294007009
HypersensitivityReaction	294008004
HypersensitivityReaction	294009007
HypersensitivityReaction	294010002
HypersensitivityReaction	294011003
HypersensitivityReaction	294012005
HypersensitivityReaction	294013000
HypersensitivityReaction	294014006
HypersensitivityReaction	294015007
HypersensitivityReaction	294016008
HypersensitivityReaction	294017004
HypersensitivityReaction	294018009
HypersensitivityReaction	294019001
HypersensitivityReaction	294021006
HypersensitivityReaction	294022004
HypersensitivityReaction	294023009
HypersensitivityReaction	294024003
HypersensitivityReaction	294025002
HypersensitivityReaction	294026001
HypersensitivityReaction	294027005
HypersensitivityReaction	294028000
HypersensitivityReaction	294029008
HypersensitivityReaction	294030003

HypersensitivityReaction	294031004
HypersensitivityReaction	294033001
HypersensitivityReaction	294035008
HypersensitivityReaction	294036009
HypersensitivityReaction	294037000
HypersensitivityReaction	294038005
HypersensitivityReaction	294039002
HypersensitivityReaction	294040000
HypersensitivityReaction	294041001
HypersensitivityReaction	294042008
HypersensitivityReaction	294043003
HypersensitivityReaction	294044009
HypersensitivityReaction	294045005
HypersensitivityReaction	294047002
HypersensitivityReaction	294048007
HypersensitivityReaction	294050004
HypersensitivityReaction	294051000
HypersensitivityReaction	294052007
HypersensitivityReaction	294053002
HypersensitivityReaction	294054008
HypersensitivityReaction	294055009
HypersensitivityReaction	294056005
HypersensitivityReaction	294057001
HypersensitivityReaction	294058006
HypersensitivityReaction	294059003
HypersensitivityReaction	294060008
HypersensitivityReaction	294061007
HypersensitivityReaction	294062000
HypersensitivityReaction	294063005
HypersensitivityReaction	294064004
HypersensitivityReaction	294067006
HypersensitivityReaction	294068001
HypersensitivityReaction	294069009
HypersensitivityReaction	294070005
HypersensitivityReaction	294071009
HypersensitivityReaction	294072002
HypersensitivityReaction	294073007
HypersensitivityReaction	294074001
HypersensitivityReaction	294075000
HypersensitivityReaction	294076004
HypersensitivityReaction	294077008
HypersensitivityReaction	294078003
HypersensitivityReaction	294079006
HypersensitivityReaction	294080009

HypersensitivityReaction	294081008
HypersensitivityReaction	294082001
HypersensitivityReaction	294083006
HypersensitivityReaction	294084000
HypersensitivityReaction	294085004
HypersensitivityReaction	294086003
HypersensitivityReaction	294087007
HypersensitivityReaction	294088002
HypersensitivityReaction	294089005
HypersensitivityReaction	294091002
HypersensitivityReaction	294092009
HypersensitivityReaction	294093004
HypersensitivityReaction	294094005
HypersensitivityReaction	294095006
HypersensitivityReaction	294096007
HypersensitivityReaction	294097003
HypersensitivityReaction	294098008
HypersensitivityReaction	294099000
HypersensitivityReaction	294100008
HypersensitivityReaction	294101007
HypersensitivityReaction	294102000
HypersensitivityReaction	294103005
HypersensitivityReaction	294104004
HypersensitivityReaction	294105003
HypersensitivityReaction	294106002
HypersensitivityReaction	294109009
HypersensitivityReaction	294110004
HypersensitivityReaction	294111000
HypersensitivityReaction	294112007
HypersensitivityReaction	294113002
HypersensitivityReaction	294114008
HypersensitivityReaction	294115009
HypersensitivityReaction	294116005
HypersensitivityReaction	294118006
HypersensitivityReaction	294119003
HypersensitivityReaction	294120009
HypersensitivityReaction	294121008
HypersensitivityReaction	294122001
HypersensitivityReaction	294123006
HypersensitivityReaction	294124000
HypersensitivityReaction	294125004
HypersensitivityReaction	294126003
HypersensitivityReaction	294127007
HypersensitivityReaction	294128002

HypersensitivityReaction	294129005
HypersensitivityReaction	294130000
HypersensitivityReaction	294131001
HypersensitivityReaction	294132008
HypersensitivityReaction	294133003
HypersensitivityReaction	294134009
HypersensitivityReaction	294135005
HypersensitivityReaction	294136006
HypersensitivityReaction	294137002
HypersensitivityReaction	294138007
HypersensitivityReaction	294139004
HypersensitivityReaction	294140002
HypersensitivityReaction	294141003
HypersensitivityReaction	294142005
HypersensitivityReaction	294143000
HypersensitivityReaction	294144006
HypersensitivityReaction	294145007
HypersensitivityReaction	294148009
HypersensitivityReaction	294149001
HypersensitivityReaction	294151002
HypersensitivityReaction	294152009
HypersensitivityReaction	294153004
HypersensitivityReaction	294156007
HypersensitivityReaction	294157003
HypersensitivityReaction	294158008
HypersensitivityReaction	294159000
HypersensitivityReaction	294160005
HypersensitivityReaction	294162002
HypersensitivityReaction	294165000
HypersensitivityReaction	294166004
HypersensitivityReaction	294168003
HypersensitivityReaction	294169006
HypersensitivityReaction	294170007
HypersensitivityReaction	294171006
HypersensitivityReaction	294172004
HypersensitivityReaction	294173009
HypersensitivityReaction	294174003
HypersensitivityReaction	294175002
HypersensitivityReaction	294176001
HypersensitivityReaction	294177005
HypersensitivityReaction	294178000
HypersensitivityReaction	294179008
HypersensitivityReaction	294180006
HypersensitivityReaction	294181005

HypersensitivityReaction	294182003
HypersensitivityReaction	294183008
HypersensitivityReaction	294184002
HypersensitivityReaction	294186000
HypersensitivityReaction	294189007
HypersensitivityReaction	294190003
HypersensitivityReaction	294191004
HypersensitivityReaction	294192006
HypersensitivityReaction	294193001
HypersensitivityReaction	294194007
HypersensitivityReaction	294197000
HypersensitivityReaction	294198005
HypersensitivityReaction	294199002
HypersensitivityReaction	294200004
HypersensitivityReaction	294201000
HypersensitivityReaction	294202007
HypersensitivityReaction	294203002
HypersensitivityReaction	294204008
HypersensitivityReaction	294206005
HypersensitivityReaction	294207001
HypersensitivityReaction	294208006
HypersensitivityReaction	294209003
HypersensitivityReaction	294210008
HypersensitivityReaction	294211007
HypersensitivityReaction	294214004
HypersensitivityReaction	294215003
HypersensitivityReaction	294217006
HypersensitivityReaction	294218001
HypersensitivityReaction	294219009
HypersensitivityReaction	294220003
HypersensitivityReaction	294221004
HypersensitivityReaction	294222006
HypersensitivityReaction	294223001
HypersensitivityReaction	294224007
HypersensitivityReaction	294225008
HypersensitivityReaction	294226009
HypersensitivityReaction	294227000
HypersensitivityReaction	294228005
HypersensitivityReaction	294229002
HypersensitivityReaction	294230007
HypersensitivityReaction	294231006
HypersensitivityReaction	294232004
HypersensitivityReaction	294233009
HypersensitivityReaction	294234003

HypersensitivityReaction	294235002
HypersensitivityReaction	294236001
HypersensitivityReaction	294237005
HypersensitivityReaction	294238000
HypersensitivityReaction	294239008
HypersensitivityReaction	294240005
HypersensitivityReaction	294242002
HypersensitivityReaction	294243007
HypersensitivityReaction	294245000
HypersensitivityReaction	294246004
HypersensitivityReaction	294247008
HypersensitivityReaction	294248003
HypersensitivityReaction	294249006
HypersensitivityReaction	294250006
HypersensitivityReaction	294252003
HypersensitivityReaction	294253008
HypersensitivityReaction	294254002
HypersensitivityReaction	294255001
HypersensitivityReaction	294256000
HypersensitivityReaction	294257009
HypersensitivityReaction	294258004
HypersensitivityReaction	294259007
HypersensitivityReaction	294260002
HypersensitivityReaction	294261003
HypersensitivityReaction	294262005
HypersensitivityReaction	294263000
HypersensitivityReaction	294264006
HypersensitivityReaction	294265007
HypersensitivityReaction	294266008
HypersensitivityReaction	294267004
HypersensitivityReaction	294268009
HypersensitivityReaction	294269001
HypersensitivityReaction	294270000
HypersensitivityReaction	294271001
HypersensitivityReaction	294272008
HypersensitivityReaction	294273003
HypersensitivityReaction	294274009
HypersensitivityReaction	294275005
HypersensitivityReaction	294276006
HypersensitivityReaction	294277002
HypersensitivityReaction	294278007
HypersensitivityReaction	294279004
HypersensitivityReaction	294280001
HypersensitivityReaction	294281002

HypersensitivityReaction	294282009
HypersensitivityReaction	294283004
HypersensitivityReaction	294284005
HypersensitivityReaction	294285006
HypersensitivityReaction	294286007
HypersensitivityReaction	294287003
HypersensitivityReaction	294288008
HypersensitivityReaction	294289000
HypersensitivityReaction	294290009
HypersensitivityReaction	294291008
HypersensitivityReaction	294297007
HypersensitivityReaction	294298002
HypersensitivityReaction	294299005
HypersensitivityReaction	294302005
HypersensitivityReaction	294304006
HypersensitivityReaction	294305007
HypersensitivityReaction	294306008
HypersensitivityReaction	294307004
HypersensitivityReaction	294310006
HypersensitivityReaction	294311005
HypersensitivityReaction	294313008
HypersensitivityReaction	294315001
HypersensitivityReaction	294316000
HypersensitivityReaction	294317009
HypersensitivityReaction	294318004
HypersensitivityReaction	294319007
HypersensitivityReaction	294320001
HypersensitivityReaction	294324005
HypersensitivityReaction	294325006
HypersensitivityReaction	294326007
HypersensitivityReaction	294327003
HypersensitivityReaction	294328008
HypersensitivityReaction	294329000
HypersensitivityReaction	294330005
HypersensitivityReaction	294332002
HypersensitivityReaction	294333007
HypersensitivityReaction	294334001
HypersensitivityReaction	294335000
HypersensitivityReaction	294336004
HypersensitivityReaction	294337008
HypersensitivityReaction	294338003
HypersensitivityReaction	294339006
HypersensitivityReaction	294340008
HypersensitivityReaction	294341007

HypersensitivityReaction	294342000
HypersensitivityReaction	294343005
HypersensitivityReaction	294344004
HypersensitivityReaction	294346002
HypersensitivityReaction	294348001
HypersensitivityReaction	294349009
HypersensitivityReaction	294350009
HypersensitivityReaction	294351008
HypersensitivityReaction	294352001
HypersensitivityReaction	294354000
HypersensitivityReaction	294355004
HypersensitivityReaction	294356003
HypersensitivityReaction	294357007
HypersensitivityReaction	294358002
HypersensitivityReaction	294359005
HypersensitivityReaction	294360000
HypersensitivityReaction	294361001
HypersensitivityReaction	294362008
HypersensitivityReaction	294363003
HypersensitivityReaction	294364009
HypersensitivityReaction	294365005
HypersensitivityReaction	294366006
HypersensitivityReaction	294367002
HypersensitivityReaction	294368007
HypersensitivityReaction	294369004
HypersensitivityReaction	294370003
HypersensitivityReaction	294371004
HypersensitivityReaction	294372006
HypersensitivityReaction	294373001
HypersensitivityReaction	294374007
HypersensitivityReaction	294375008
HypersensitivityReaction	294376009
HypersensitivityReaction	294377000
HypersensitivityReaction	294378005
HypersensitivityReaction	294379002
HypersensitivityReaction	294380004
HypersensitivityReaction	294381000
HypersensitivityReaction	294382007
HypersensitivityReaction	294383002
HypersensitivityReaction	294384008
HypersensitivityReaction	294385009
HypersensitivityReaction	294386005
HypersensitivityReaction	294387001
HypersensitivityReaction	294388006

HypersensitivityReaction	294389003
HypersensitivityReaction	294390007
HypersensitivityReaction	294391006
HypersensitivityReaction	294392004
HypersensitivityReaction	294393009
HypersensitivityReaction	294394003
HypersensitivityReaction	294395002
HypersensitivityReaction	294396001
HypersensitivityReaction	294397005
HypersensitivityReaction	294398000
HypersensitivityReaction	294399008
HypersensitivityReaction	294400001
HypersensitivityReaction	294401002
HypersensitivityReaction	294404005
HypersensitivityReaction	294405006
HypersensitivityReaction	294406007
HypersensitivityReaction	294407003
HypersensitivityReaction	294408008
HypersensitivityReaction	294409000
HypersensitivityReaction	294410005
HypersensitivityReaction	294411009
HypersensitivityReaction	294412002
HypersensitivityReaction	294413007
HypersensitivityReaction	294414001
HypersensitivityReaction	294415000
HypersensitivityReaction	294416004
HypersensitivityReaction	294417008
HypersensitivityReaction	294418003
HypersensitivityReaction	294421001
HypersensitivityReaction	294422008
HypersensitivityReaction	294423003
HypersensitivityReaction	294424009
HypersensitivityReaction	294425005
HypersensitivityReaction	294426006
HypersensitivityReaction	294427002
HypersensitivityReaction	294428007
HypersensitivityReaction	294429004
HypersensitivityReaction	294430009
HypersensitivityReaction	294431008
HypersensitivityReaction	294432001
HypersensitivityReaction	294433006
HypersensitivityReaction	294434000
HypersensitivityReaction	294436003
HypersensitivityReaction	294437007

HypersensitivityReaction	294438002
HypersensitivityReaction	294439005
HypersensitivityReaction	294440007
HypersensitivityReaction	294441006
HypersensitivityReaction	294442004
HypersensitivityReaction	294443009
HypersensitivityReaction	294445002
HypersensitivityReaction	294446001
HypersensitivityReaction	294447005
HypersensitivityReaction	294448000
HypersensitivityReaction	294449008
HypersensitivityReaction	294450008
HypersensitivityReaction	294451007
HypersensitivityReaction	294452000
HypersensitivityReaction	294453005
HypersensitivityReaction	294455003
HypersensitivityReaction	294456002
HypersensitivityReaction	294457006
HypersensitivityReaction	294458001
HypersensitivityReaction	294459009
HypersensitivityReaction	294460004
HypersensitivityReaction	294461000
HypersensitivityReaction	294462007
HypersensitivityReaction	294463002
HypersensitivityReaction	294464008
HypersensitivityReaction	294465009
HypersensitivityReaction	294466005
HypersensitivityReaction	294467001
HypersensitivityReaction	294468006
HypersensitivityReaction	294469003
HypersensitivityReaction	294470002
HypersensitivityReaction	294471003
HypersensitivityReaction	294472005
HypersensitivityReaction	294473000
HypersensitivityReaction	294474006
HypersensitivityReaction	294475007
HypersensitivityReaction	294476008
HypersensitivityReaction	294477004
HypersensitivityReaction	294478009
HypersensitivityReaction	294479001
HypersensitivityReaction	294480003
HypersensitivityReaction	294481004
HypersensitivityReaction	294482006
HypersensitivityReaction	294483001

HypersensitivityReaction	294484007
HypersensitivityReaction	294485008
HypersensitivityReaction	294486009
HypersensitivityReaction	294487000
HypersensitivityReaction	294488005
HypersensitivityReaction	294489002
HypersensitivityReaction	294490006
HypersensitivityReaction	294491005
HypersensitivityReaction	294492003
HypersensitivityReaction	294494002
HypersensitivityReaction	294495001
HypersensitivityReaction	294496000
HypersensitivityReaction	294497009
HypersensitivityReaction	294498004
HypersensitivityReaction	294499007
HypersensitivityReaction	294500003
HypersensitivityReaction	294501004
HypersensitivityReaction	294502006
HypersensitivityReaction	294503001
HypersensitivityReaction	294504007
HypersensitivityReaction	294505008
HypersensitivityReaction	294506009
HypersensitivityReaction	294507000
HypersensitivityReaction	294508005
HypersensitivityReaction	294509002
HypersensitivityReaction	294510007
HypersensitivityReaction	294511006
HypersensitivityReaction	294512004
HypersensitivityReaction	294513009
HypersensitivityReaction	294514003
HypersensitivityReaction	294515002
HypersensitivityReaction	294516001
HypersensitivityReaction	294517005
HypersensitivityReaction	294518000
HypersensitivityReaction	294519008
HypersensitivityReaction	294520002
HypersensitivityReaction	294521003
HypersensitivityReaction	294522005
HypersensitivityReaction	294523000
HypersensitivityReaction	294524006
HypersensitivityReaction	294525007
HypersensitivityReaction	294526008
HypersensitivityReaction	294527004
HypersensitivityReaction	294528009

HypersensitivityReaction	294529001
HypersensitivityReaction	294530006
HypersensitivityReaction	294531005
HypersensitivityReaction	294532003
HypersensitivityReaction	294533008
HypersensitivityReaction	294534002
HypersensitivityReaction	294535001
HypersensitivityReaction	294536000
HypersensitivityReaction	294537009
HypersensitivityReaction	294538004
HypersensitivityReaction	294539007
HypersensitivityReaction	294540009
HypersensitivityReaction	294541008
HypersensitivityReaction	294542001
HypersensitivityReaction	294543006
HypersensitivityReaction	294544000
HypersensitivityReaction	294545004
HypersensitivityReaction	294546003
HypersensitivityReaction	294547007
HypersensitivityReaction	294548002
HypersensitivityReaction	294549005
HypersensitivityReaction	294550005
HypersensitivityReaction	294551009
HypersensitivityReaction	294552002
HypersensitivityReaction	294554001
HypersensitivityReaction	294555000
HypersensitivityReaction	294556004
HypersensitivityReaction	294557008
HypersensitivityReaction	294558003
HypersensitivityReaction	294559006
HypersensitivityReaction	294560001
HypersensitivityReaction	294561002
HypersensitivityReaction	294562009
HypersensitivityReaction	294563004
HypersensitivityReaction	294564005
HypersensitivityReaction	294565006
HypersensitivityReaction	294566007
HypersensitivityReaction	294567003
HypersensitivityReaction	294568008
HypersensitivityReaction	294569000
HypersensitivityReaction	294570004
HypersensitivityReaction	294571000
HypersensitivityReaction	294572007
HypersensitivityReaction	294573002

HypersensitivityReaction	294574008
HypersensitivityReaction	294575009
HypersensitivityReaction	294576005
HypersensitivityReaction	294577001
HypersensitivityReaction	294578006
HypersensitivityReaction	294579003
HypersensitivityReaction	294580000
HypersensitivityReaction	294581001
HypersensitivityReaction	294582008
HypersensitivityReaction	294583003
HypersensitivityReaction	294584009
HypersensitivityReaction	294585005
HypersensitivityReaction	294586006
HypersensitivityReaction	294587002
HypersensitivityReaction	294588007
HypersensitivityReaction	294589004
HypersensitivityReaction	294590008
HypersensitivityReaction	294591007
HypersensitivityReaction	294592000
HypersensitivityReaction	294593005
HypersensitivityReaction	294594004
HypersensitivityReaction	294595003
HypersensitivityReaction	294596002
HypersensitivityReaction	294597006
HypersensitivityReaction	294598001
HypersensitivityReaction	294599009
HypersensitivityReaction	294600007
HypersensitivityReaction	294601006
HypersensitivityReaction	294602004
HypersensitivityReaction	294603009
HypersensitivityReaction	294604003
HypersensitivityReaction	294605002
HypersensitivityReaction	294606001
HypersensitivityReaction	294607005
HypersensitivityReaction	294608000
HypersensitivityReaction	294609008
HypersensitivityReaction	294610003
HypersensitivityReaction	294611004
HypersensitivityReaction	294612006
HypersensitivityReaction	294613001
HypersensitivityReaction	294614007
HypersensitivityReaction	294615008
HypersensitivityReaction	294616009
HypersensitivityReaction	294617000

HypersensitivityReaction	294618005
HypersensitivityReaction	294619002
HypersensitivityReaction	294620008
HypersensitivityReaction	294621007
HypersensitivityReaction	294622000
HypersensitivityReaction	294623005
HypersensitivityReaction	294624004
HypersensitivityReaction	294625003
HypersensitivityReaction	294626002
HypersensitivityReaction	294627006
HypersensitivityReaction	294628001
HypersensitivityReaction	294629009
HypersensitivityReaction	294630004
HypersensitivityReaction	294631000
HypersensitivityReaction	294632007
HypersensitivityReaction	294633002
HypersensitivityReaction	294634008
HypersensitivityReaction	294635009
HypersensitivityReaction	294636005
HypersensitivityReaction	294637001
HypersensitivityReaction	294638006
HypersensitivityReaction	294639003
HypersensitivityReaction	294640001
HypersensitivityReaction	294641002
HypersensitivityReaction	294642009
HypersensitivityReaction	294643004
HypersensitivityReaction	294644005
HypersensitivityReaction	294645006
HypersensitivityReaction	294646007
HypersensitivityReaction	294647003
HypersensitivityReaction	294648008
HypersensitivityReaction	294649000
HypersensitivityReaction	294650000
HypersensitivityReaction	294651001
HypersensitivityReaction	294652008
HypersensitivityReaction	294654009
HypersensitivityReaction	294655005
HypersensitivityReaction	294656006
HypersensitivityReaction	294657002
HypersensitivityReaction	294658007
HypersensitivityReaction	294659004
HypersensitivityReaction	294660009
HypersensitivityReaction	294661008
HypersensitivityReaction	294662001

HypersensitivityReaction	294663006
HypersensitivityReaction	294664000
HypersensitivityReaction	294665004
HypersensitivityReaction	294666003
HypersensitivityReaction	294667007
HypersensitivityReaction	294668002
HypersensitivityReaction	294669005
HypersensitivityReaction	294670006
HypersensitivityReaction	294671005
HypersensitivityReaction	294672003
HypersensitivityReaction	294673008
HypersensitivityReaction	294674002
HypersensitivityReaction	294675001
HypersensitivityReaction	294676000
HypersensitivityReaction	294677009
HypersensitivityReaction	294678004
HypersensitivityReaction	294679007
HypersensitivityReaction	294680005
HypersensitivityReaction	294681009
HypersensitivityReaction	294682002
HypersensitivityReaction	294683007
HypersensitivityReaction	294684001
HypersensitivityReaction	294685000
HypersensitivityReaction	294686004
HypersensitivityReaction	294687008
HypersensitivityReaction	294688003
HypersensitivityReaction	294689006
HypersensitivityReaction	294690002
HypersensitivityReaction	294691003
HypersensitivityReaction	294692005
HypersensitivityReaction	294693000
HypersensitivityReaction	294694006
HypersensitivityReaction	294695007
HypersensitivityReaction	294696008
HypersensitivityReaction	294697004
HypersensitivityReaction	294698009
HypersensitivityReaction	294699001
HypersensitivityReaction	294700000
HypersensitivityReaction	294701001
HypersensitivityReaction	294702008
HypersensitivityReaction	294703003
HypersensitivityReaction	294704009
HypersensitivityReaction	294706006
HypersensitivityReaction	294707002

HypersensitivityReaction	294708007
HypersensitivityReaction	294709004
HypersensitivityReaction	294710009
HypersensitivityReaction	294711008
HypersensitivityReaction	294712001
HypersensitivityReaction	294713006
HypersensitivityReaction	294714000
HypersensitivityReaction	294715004
HypersensitivityReaction	294716003
HypersensitivityReaction	294717007
HypersensitivityReaction	294718002
HypersensitivityReaction	294719005
HypersensitivityReaction	294720004
HypersensitivityReaction	294721000
HypersensitivityReaction	294722007
HypersensitivityReaction	294723002
HypersensitivityReaction	294724008
HypersensitivityReaction	294725009
HypersensitivityReaction	294726005
HypersensitivityReaction	294727001
HypersensitivityReaction	294728006
HypersensitivityReaction	294729003
HypersensitivityReaction	294730008
HypersensitivityReaction	294731007
HypersensitivityReaction	294732000
HypersensitivityReaction	294733005
HypersensitivityReaction	294734004
HypersensitivityReaction	294735003
HypersensitivityReaction	294736002
HypersensitivityReaction	294737006
HypersensitivityReaction	294738001
HypersensitivityReaction	294739009
HypersensitivityReaction	294740006
HypersensitivityReaction	294741005
HypersensitivityReaction	294742003
HypersensitivityReaction	294744002
HypersensitivityReaction	294745001
HypersensitivityReaction	294746000
HypersensitivityReaction	294747009
HypersensitivityReaction	294748004
HypersensitivityReaction	294749007
HypersensitivityReaction	294750007
HypersensitivityReaction	294751006
HypersensitivityReaction	294752004

HypersensitivityReaction	294753009
HypersensitivityReaction	294754003
HypersensitivityReaction	294755002
HypersensitivityReaction	294757005
HypersensitivityReaction	294758000
HypersensitivityReaction	294759008
HypersensitivityReaction	294760003
HypersensitivityReaction	294761004
HypersensitivityReaction	294762006
HypersensitivityReaction	294763001
HypersensitivityReaction	294764007
HypersensitivityReaction	294765008
HypersensitivityReaction	294766009
HypersensitivityReaction	294767000
HypersensitivityReaction	294768005
HypersensitivityReaction	294769002
HypersensitivityReaction	294770001
HypersensitivityReaction	294771002
HypersensitivityReaction	294773004
HypersensitivityReaction	294774005
HypersensitivityReaction	294775006
HypersensitivityReaction	294776007
HypersensitivityReaction	294777003
HypersensitivityReaction	294778008
HypersensitivityReaction	294779000
HypersensitivityReaction	294780002
HypersensitivityReaction	294781003
HypersensitivityReaction	294782005
HypersensitivityReaction	294783000
HypersensitivityReaction	294784006
HypersensitivityReaction	294785007
HypersensitivityReaction	294786008
HypersensitivityReaction	294787004
HypersensitivityReaction	294788009
HypersensitivityReaction	294789001
HypersensitivityReaction	294790005
HypersensitivityReaction	294791009
HypersensitivityReaction	294792002
HypersensitivityReaction	294793007
HypersensitivityReaction	294794001
HypersensitivityReaction	294795000
HypersensitivityReaction	294796004
HypersensitivityReaction	294797008
HypersensitivityReaction	294798003

HypersensitivityReaction	294799006
HypersensitivityReaction	294800005
HypersensitivityReaction	294801009
HypersensitivityReaction	294802002
HypersensitivityReaction	294803007
HypersensitivityReaction	294804001
HypersensitivityReaction	294805000
HypersensitivityReaction	294806004
HypersensitivityReaction	294807008
HypersensitivityReaction	294808003
HypersensitivityReaction	294809006
HypersensitivityReaction	294810001
HypersensitivityReaction	294811002
HypersensitivityReaction	294812009
HypersensitivityReaction	294813004
HypersensitivityReaction	294814005
HypersensitivityReaction	294815006
HypersensitivityReaction	294816007
HypersensitivityReaction	294817003
HypersensitivityReaction	294818008
HypersensitivityReaction	294819000
HypersensitivityReaction	294820006
HypersensitivityReaction	294821005
HypersensitivityReaction	294822003
HypersensitivityReaction	294823008
HypersensitivityReaction	294824002
HypersensitivityReaction	294825001
HypersensitivityReaction	294826000
HypersensitivityReaction	294827009
HypersensitivityReaction	294828004
HypersensitivityReaction	294829007
HypersensitivityReaction	294830002
HypersensitivityReaction	294833000
HypersensitivityReaction	294834006
HypersensitivityReaction	294835007
HypersensitivityReaction	294836008
HypersensitivityReaction	294837004
HypersensitivityReaction	294838009
HypersensitivityReaction	294839001
HypersensitivityReaction	294840004
HypersensitivityReaction	294841000
HypersensitivityReaction	294843002
HypersensitivityReaction	294844008
HypersensitivityReaction	294845009

HypersensitivityReaction	294846005
HypersensitivityReaction	294847001
HypersensitivityReaction	294848006
HypersensitivityReaction	294849003
HypersensitivityReaction	294850003
HypersensitivityReaction	294851004
HypersensitivityReaction	294852006
HypersensitivityReaction	294853001
HypersensitivityReaction	294854007
HypersensitivityReaction	294855008
HypersensitivityReaction	294856009
HypersensitivityReaction	294857000
HypersensitivityReaction	294859002
HypersensitivityReaction	294860007
HypersensitivityReaction	294861006
HypersensitivityReaction	294862004
HypersensitivityReaction	294863009
HypersensitivityReaction	294864003
HypersensitivityReaction	294865002
HypersensitivityReaction	294866001
HypersensitivityReaction	294867005
HypersensitivityReaction	294868000
HypersensitivityReaction	294869008
HypersensitivityReaction	294870009
HypersensitivityReaction	294871008
HypersensitivityReaction	294872001
HypersensitivityReaction	294873006
HypersensitivityReaction	294874000
HypersensitivityReaction	294875004
HypersensitivityReaction	294876003
HypersensitivityReaction	294877007
HypersensitivityReaction	294878002
HypersensitivityReaction	294879005
HypersensitivityReaction	294880008
HypersensitivityReaction	294881007
HypersensitivityReaction	294882000
HypersensitivityReaction	294883005
HypersensitivityReaction	294884004
HypersensitivityReaction	294885003
HypersensitivityReaction	294886002
HypersensitivityReaction	294887006
HypersensitivityReaction	294888001
HypersensitivityReaction	294889009
HypersensitivityReaction	294891001

HypersensitivityReaction	294892008
HypersensitivityReaction	294893003
HypersensitivityReaction	294894009
HypersensitivityReaction	294895005
HypersensitivityReaction	294896006
HypersensitivityReaction	294897002
HypersensitivityReaction	294898007
HypersensitivityReaction	294899004
HypersensitivityReaction	294900009
HypersensitivityReaction	294901008
HypersensitivityReaction	294902001
HypersensitivityReaction	294903006
HypersensitivityReaction	294904000
HypersensitivityReaction	294905004
HypersensitivityReaction	294906003
HypersensitivityReaction	294907007
HypersensitivityReaction	294908002
HypersensitivityReaction	294909005
HypersensitivityReaction	294910000
HypersensitivityReaction	294911001
HypersensitivityReaction	294912008
HypersensitivityReaction	294913003
HypersensitivityReaction	294914009
HypersensitivityReaction	294915005
HypersensitivityReaction	294916006
HypersensitivityReaction	294917002
HypersensitivityReaction	294920005
HypersensitivityReaction	294921009
HypersensitivityReaction	294922002
HypersensitivityReaction	294923007
HypersensitivityReaction	294924001
HypersensitivityReaction	294925000
HypersensitivityReaction	294926004
HypersensitivityReaction	294927008
HypersensitivityReaction	294928003
HypersensitivityReaction	294929006
HypersensitivityReaction	294930001
HypersensitivityReaction	294931002
HypersensitivityReaction	294932009
HypersensitivityReaction	294933004
HypersensitivityReaction	294934005
HypersensitivityReaction	294935006
HypersensitivityReaction	294936007
HypersensitivityReaction	294937003

HypersensitivityReaction	294938008
HypersensitivityReaction	294939000
HypersensitivityReaction	294940003
HypersensitivityReaction	294941004
HypersensitivityReaction	294942006
HypersensitivityReaction	294943001
HypersensitivityReaction	294944007
HypersensitivityReaction	294945008
HypersensitivityReaction	294950002
HypersensitivityReaction	294951003
HypersensitivityReaction	294952005
HypersensitivityReaction	294955007
HypersensitivityReaction	294956008
HypersensitivityReaction	294957004
HypersensitivityReaction	294958009
HypersensitivityReaction	294960006
HypersensitivityReaction	294961005
HypersensitivityReaction	294962003
HypersensitivityReaction	294963008
HypersensitivityReaction	294964002
HypersensitivityReaction	294965001
HypersensitivityReaction	294966000
HypersensitivityReaction	294967009
HypersensitivityReaction	294968004
HypersensitivityReaction	294969007
HypersensitivityReaction	294970008
HypersensitivityReaction	294971007
HypersensitivityReaction	294972000
HypersensitivityReaction	294973005
HypersensitivityReaction	294975003
HypersensitivityReaction	294976002
HypersensitivityReaction	294977006
HypersensitivityReaction	294978001
HypersensitivityReaction	294979009
HypersensitivityReaction	294980007
HypersensitivityReaction	294981006
HypersensitivityReaction	294982004
HypersensitivityReaction	294983009
HypersensitivityReaction	294984003
HypersensitivityReaction	294985002
HypersensitivityReaction	294986001
HypersensitivityReaction	294987005
HypersensitivityReaction	294988000
HypersensitivityReaction	294989008

HypersensitivityReaction	294990004
HypersensitivityReaction	294991000
HypersensitivityReaction	294992007
HypersensitivityReaction	294993002
HypersensitivityReaction	294994008
HypersensitivityReaction	294995009
HypersensitivityReaction	294996005
HypersensitivityReaction	294997001
HypersensitivityReaction	294998006
HypersensitivityReaction	294999003
HypersensitivityReaction	295000003
HypersensitivityReaction	295001004
HypersensitivityReaction	295002006
HypersensitivityReaction	295003001
HypersensitivityReaction	295004007
HypersensitivityReaction	295005008
HypersensitivityReaction	295006009
HypersensitivityReaction	295007000
HypersensitivityReaction	295008005
HypersensitivityReaction	295009002
HypersensitivityReaction	295010007
HypersensitivityReaction	295011006
HypersensitivityReaction	295012004
HypersensitivityReaction	295013009
HypersensitivityReaction	295014003
HypersensitivityReaction	295015002
HypersensitivityReaction	295016001
HypersensitivityReaction	295017005
HypersensitivityReaction	295018000
HypersensitivityReaction	295019008
HypersensitivityReaction	295020002
HypersensitivityReaction	295021003
HypersensitivityReaction	295022005
HypersensitivityReaction	295023000
HypersensitivityReaction	295024006
HypersensitivityReaction	295025007
HypersensitivityReaction	295026008
HypersensitivityReaction	295027004
HypersensitivityReaction	295028009
HypersensitivityReaction	295029001
HypersensitivityReaction	295030006
HypersensitivityReaction	295031005
HypersensitivityReaction	295033008
HypersensitivityReaction	295034002

HypersensitivityReaction	295035001
HypersensitivityReaction	295036000
HypersensitivityReaction	295037009
HypersensitivityReaction	295038004
HypersensitivityReaction	295039007
HypersensitivityReaction	295040009
HypersensitivityReaction	295041008
HypersensitivityReaction	295042001
HypersensitivityReaction	295043006
HypersensitivityReaction	295044000
HypersensitivityReaction	295045004
HypersensitivityReaction	295048002
HypersensitivityReaction	295049005
HypersensitivityReaction	295050005
HypersensitivityReaction	295051009
HypersensitivityReaction	295053007
HypersensitivityReaction	295054001
HypersensitivityReaction	295055000
HypersensitivityReaction	295056004
HypersensitivityReaction	295057008
HypersensitivityReaction	295058003
HypersensitivityReaction	295059006
HypersensitivityReaction	295060001
HypersensitivityReaction	295062009
HypersensitivityReaction	295064005
HypersensitivityReaction	295065006
HypersensitivityReaction	295066007
HypersensitivityReaction	295067003
HypersensitivityReaction	295068008
HypersensitivityReaction	295069000
HypersensitivityReaction	295070004
HypersensitivityReaction	295071000
HypersensitivityReaction	295072007
HypersensitivityReaction	295073002
HypersensitivityReaction	295074008
HypersensitivityReaction	295075009
HypersensitivityReaction	295076005
HypersensitivityReaction	295077001
HypersensitivityReaction	295078006
HypersensitivityReaction	295079003
HypersensitivityReaction	295080000
HypersensitivityReaction	295081001
HypersensitivityReaction	295082008
HypersensitivityReaction	295083003

HypersensitivityReaction	295084009
HypersensitivityReaction	295085005
HypersensitivityReaction	295086006
HypersensitivityReaction	295087002
HypersensitivityReaction	295088007
HypersensitivityReaction	295089004
HypersensitivityReaction	295090008
HypersensitivityReaction	295091007
HypersensitivityReaction	295092000
HypersensitivityReaction	295093005
HypersensitivityReaction	295094004
HypersensitivityReaction	295095003
HypersensitivityReaction	295096002
HypersensitivityReaction	295097006
HypersensitivityReaction	295098001
HypersensitivityReaction	295099009
HypersensitivityReaction	295100001
HypersensitivityReaction	295101002
HypersensitivityReaction	295102009
HypersensitivityReaction	295103004
HypersensitivityReaction	295105006
HypersensitivityReaction	295106007
HypersensitivityReaction	295108008
HypersensitivityReaction	295109000
HypersensitivityReaction	295110005
HypersensitivityReaction	295111009
HypersensitivityReaction	295112002
HypersensitivityReaction	295113007
HypersensitivityReaction	295114001
HypersensitivityReaction	295115000
HypersensitivityReaction	295116004
HypersensitivityReaction	297938000
HypersensitivityReaction	300909004
HypersensitivityReaction	300910009
HypersensitivityReaction	300911008
HypersensitivityReaction	300912001
HypersensitivityReaction	300913006
HypersensitivityReaction	300914000
HypersensitivityReaction	300915004
HypersensitivityReaction	300916003
HypersensitivityReaction	312664009
HypersensitivityReaction	312665005
HypersensitivityReaction	315334000
HypersensitivityReaction	315631004

HypersensitivityReaction	315781002
HypersensitivityReaction	315782009
HypersensitivityReaction	315783004
HypersensitivityReaction	315784005
HypersensitivityReaction	315785006
HypersensitivityReaction	315786007
HypersensitivityReaction	315787003
HypersensitivityReaction	315788008
HypersensitivityReaction	315789000
HypersensitivityReaction	315792001
HypersensitivityReaction	315793006
HypersensitivityReaction	316850008
HypersensitivityReaction	316851007
HypersensitivityReaction	316852000
HypersensitivityReaction	320891004
HypersensitivityReaction	359804008
HypersensitivityReaction	361098001
HypersensitivityReaction	363008002
HypersensitivityReaction	387198004
HypersensitivityReaction	389145006
HypersensitivityReaction	389146007
HypersensitivityReaction	390952000
HypersensitivityReaction	395102008
HypersensitivityReaction	400195000
HypersensitivityReaction	402245001
HypersensitivityReaction	402251006
HypersensitivityReaction	402289001
HypersensitivityReaction	402306009
HypersensitivityReaction	402390008
HypersensitivityReaction	402391007
HypersensitivityReaction	402395003
HypersensitivityReaction	402396002
HypersensitivityReaction	402416000
HypersensitivityReaction	402591008
HypersensitivityReaction	402595004
HypersensitivityReaction	402597007
HypersensitivityReaction	402656007
HypersensitivityReaction	402855009
HypersensitivityReaction	403510002
HypersensitivityReaction	404807005
HypersensitivityReaction	405720007
HypersensitivityReaction	407577009
HypersensitivityReaction	407579007
HypersensitivityReaction	407580005

HypersensitivityReaction	407589006
HypersensitivityReaction	407591003
HypersensitivityReaction	407593000
HypersensitivityReaction	407594006
HypersensitivityReaction	407595007
HypersensitivityReaction	407674008
HypersensitivityReaction	409136006
HypersensitivityReaction	409638006
HypersensitivityReaction	409639003
HypersensitivityReaction	409640001
HypersensitivityReaction	413363003
HypersensitivityReaction	414285001
HypersensitivityReaction	414373006
HypersensitivityReaction	416093006
HypersensitivityReaction	416098002
HypersensitivityReaction	416373009
HypersensitivityReaction	416503009
HypersensitivityReaction	416858000
HypersensitivityReaction	416948000
HypersensitivityReaction	417335004
HypersensitivityReaction	417424008
HypersensitivityReaction	417516000
HypersensitivityReaction	417532002
HypersensitivityReaction	417556003
HypersensitivityReaction	417644009
HypersensitivityReaction	417671009
HypersensitivityReaction	417918006
HypersensitivityReaction	417930000
HypersensitivityReaction	417960005
HypersensitivityReaction	418032008
HypersensitivityReaction	418051002
HypersensitivityReaction	418085001
HypersensitivityReaction	418176003
HypersensitivityReaction	418184004
HypersensitivityReaction	418282006
HypersensitivityReaction	418314004
HypersensitivityReaction	418325008
HypersensitivityReaction	418344001
HypersensitivityReaction	418364006
HypersensitivityReaction	418367004
HypersensitivityReaction	418397007
HypersensitivityReaction	418434002
HypersensitivityReaction	418448002
HypersensitivityReaction	418484009

HypersensitivityReaction	418561004
HypersensitivityReaction	418579000
HypersensitivityReaction	418606003
HypersensitivityReaction	418626004
HypersensitivityReaction	418634005
HypersensitivityReaction	418689008
HypersensitivityReaction	418737008
HypersensitivityReaction	418779002
HypersensitivityReaction	418809008
HypersensitivityReaction	418815008
HypersensitivityReaction	418878004
HypersensitivityReaction	418925002
HypersensitivityReaction	418943003
HypersensitivityReaction	419042001
HypersensitivityReaction	419063004
HypersensitivityReaction	419076005
HypersensitivityReaction	419101002
HypersensitivityReaction	419180003
HypersensitivityReaction	419199007
HypersensitivityReaction	419210001
HypersensitivityReaction	419238009
HypersensitivityReaction	419263009
HypersensitivityReaction	419271008
HypersensitivityReaction	419298007
HypersensitivityReaction	419342009
HypersensitivityReaction	419375001
HypersensitivityReaction	419412007
HypersensitivityReaction	419421008
HypersensitivityReaction	419447004
HypersensitivityReaction	419452009
HypersensitivityReaction	419474003
HypersensitivityReaction	419515007
HypersensitivityReaction	419519001
HypersensitivityReaction	419573007
HypersensitivityReaction	419619007
HypersensitivityReaction	419666006
HypersensitivityReaction	419788000
HypersensitivityReaction	419814004
HypersensitivityReaction	419838000
HypersensitivityReaction	419884005
HypersensitivityReaction	419930008
HypersensitivityReaction	419967000
HypersensitivityReaction	419972009
HypersensitivityReaction	420080006

HypersensitivityReaction	420091004
HypersensitivityReaction	420140004
HypersensitivityReaction	420174000
HypersensitivityReaction	420198007
HypersensitivityReaction	420740008
HypersensitivityReaction	421492009
HypersensitivityReaction	421961002
HypersensitivityReaction	422921000
HypersensitivityReaction	423058007
HypersensitivityReaction	423889005
HypersensitivityReaction	424213003
HypersensitivityReaction	424643009
HypersensitivityReaction	425011002
HypersensitivityReaction	425525006
HypersensitivityReaction	425605001
HypersensitivityReaction	426232007
HypersensitivityReaction	427487000
HypersensitivityReaction	427833000
HypersensitivityReaction	427903006
HypersensitivityReaction	428392002
HypersensitivityReaction	428795003
HypersensitivityReaction	428980005
HypersensitivityReaction	429204002
HypersensitivityReaction	429239002
HypersensitivityReaction	429751004
HypersensitivityReaction	430980000
HypersensitivityReaction	432807008
HypersensitivityReaction	432989008
HypersensitivityReaction	439405005
HypersensitivityReaction	439406006
HypersensitivityReaction	439954005
HypersensitivityReaction	441492003
HypersensitivityReaction	441495001
HypersensitivityReaction	441593005
HypersensitivityReaction	441725009
HypersensitivityReaction	441814005
HypersensitivityReaction	441931002
HypersensitivityReaction	441954006
HypersensitivityReaction	441955007
HypersensitivityReaction	441992007
HypersensitivityReaction	442022002
HypersensitivityReaction	442052005
HypersensitivityReaction	442408006
HypersensitivityReaction	444026000

HypersensitivityReaction	444316004
HypersensitivityReaction	445395006
HypersensitivityReaction	445932004
HypersensitivityReaction	447961002
HypersensitivityReaction	448438007
HypersensitivityReaction	448690007
HypersensitivityReaction	449324007
HypersensitivityReaction	449414003
HypersensitivityReaction	450767000
HypersensitivityReaction	473077006
HypersensitivityReaction	473078001
HypersensitivityReaction	473080007
HypersensitivityReaction	473081006
HypersensitivityReaction	473104002
HypersensitivityReaction	473105001
HypersensitivityReaction	609328004
HypersensitivityReaction	609396006
HypersensitivityReaction	609397002
HypersensitivityReaction	609398007
HypersensitivityReaction	609406000
HypersensitivityReaction	609407009
HypersensitivityReaction	609409007
HypersensitivityReaction	609433001
HypersensitivityReaction	609532009
HypersensitivityReaction	609533004
HypersensitivityReaction	609534005
HypersensitivityReaction	609535006
HypersensitivityReaction	609536007
HypersensitivityReaction	609537003
HypersensitivityReaction	609538008
HypersensitivityReaction	609539000
HypersensitivityReaction	609540003
HypersensitivityReaction	609541004
HypersensitivityReaction	609542006
HypersensitivityReaction	609543001
HypersensitivityReaction	609544007
HypersensitivityReaction	609545008
HypersensitivityReaction	609546009
HypersensitivityReaction	609547000
HypersensitivityReaction	609548005
HypersensitivityReaction	609549002
HypersensitivityReaction	609551003
HypersensitivityReaction	609552005
HypersensitivityReaction	698300001

HypersensitivityReaction	699383009
HypersensitivityReaction	700468006
HypersensitivityReaction	702559001
HypersensitivityReaction	702602008
HypersensitivityReaction	702809001
HypersensitivityReaction	702810006
HypersensitivityReaction	702811005
HypersensitivityReaction	703076003
HypersensitivityReaction	703902000
HypersensitivityReaction	703911000
HypersensitivityReaction	703925004
HypersensitivityReaction	703926003
HypersensitivityReaction	703928002
HypersensitivityReaction	703930000
HypersensitivityReaction	703931001
HypersensitivityReaction	703932008
HypersensitivityReaction	703933003
HypersensitivityReaction	703934009
HypersensitivityReaction	703935005
HypersensitivityReaction	703936006
HypersensitivityReaction	703953004
HypersensitivityReaction	703954005
HypersensitivityReaction	704279007
HypersensitivityReaction	704280005
HypersensitivityReaction	705097000
HypersensitivityReaction	706887003
HypersensitivityReaction	706888008
HypersensitivityReaction	707989006
HypersensitivityReaction	708093000
HypersensitivityReaction	708095007
HypersensitivityReaction	710158004
HypersensitivityReaction	712838009
HypersensitivityReaction	712839001
HypersensitivityReaction	712840004
HypersensitivityReaction	712841000
HypersensitivityReaction	712842007
HypersensitivityReaction	712843002
HypersensitivityReaction	712844008
HypersensitivityReaction	712845009
HypersensitivityReaction	713018004
HypersensitivityReaction	713276002
HypersensitivityReaction	713296005
HypersensitivityReaction	713690006
HypersensitivityReaction	713707006

HypersensitivityReaction	713725004
HypersensitivityReaction	714035009
HypersensitivityReaction	714205005
HypersensitivityReaction	714332003
HypersensitivityReaction	715887004
HypersensitivityReaction	716187007
HypersensitivityReaction	716374005
HypersensitivityReaction	717234006
HypersensitivityReaction	718534009
HypersensitivityReaction	718535005
HypersensitivityReaction	718536006
HypersensitivityReaction	718537002
HypersensitivityReaction	719040004
HypersensitivityReaction	720691008
HypersensitivityReaction	721134005
HypersensitivityReaction	725415009
HypersensitivityReaction	725873003
HypersensitivityReaction	735173007
HypersensitivityReaction	735445000
HypersensitivityReaction	735446004
HypersensitivityReaction	735447008
HypersensitivityReaction	735448003
HypersensitivityReaction	735449006
HypersensitivityReaction	735456000
HypersensitivityReaction	735458004
HypersensitivityReaction	735460002
HypersensitivityReaction	735468009
HypersensitivityReaction	735587000
HypersensitivityReaction	735588005
HypersensitivityReaction	735933002
HypersensitivityReaction	735975001
HypersensitivityReaction	737144005
HypersensitivityReaction	737493007
HypersensitivityReaction	762455001
HypersensitivityReaction	762521001
HypersensitivityReaction	764442003
HypersensitivityReaction	767014001
HypersensitivityReaction	767015000
HypersensitivityReaction	767099007
HypersensitivityReaction	767116002
HypersensitivityReaction	767198002
HypersensitivityReaction	767203005
HypersensitivityReaction	767468001
HypersensitivityReaction	767469009

HypersensitivityReaction	767470005
HypersensitivityReaction	767473007
HypersensitivityReaction	767474001
HypersensitivityReaction	767475000
HypersensitivityReaction	767478003
HypersensitivityReaction	767479006
HypersensitivityReaction	767642003
HypersensitivityReaction	770712008
HypersensitivityReaction	770713003
HypersensitivityReaction	770714009
HypersensitivityReaction	770715005
HypersensitivityReaction	770891008
HypersensitivityReaction	772021008
HypersensitivityReaction	773399002
HypersensitivityReaction	773417002
HypersensitivityReaction	773420005
HypersensitivityReaction	773424001
HypersensitivityReaction	773427008
HypersensitivityReaction	773428003
HypersensitivityReaction	773453000
HypersensitivityReaction	773481006
HypersensitivityReaction	773482004
HypersensitivityReaction	773483009
HypersensitivityReaction	773484003
HypersensitivityReaction	773485002
HypersensitivityReaction	773486001
HypersensitivityReaction	773487005
HypersensitivityReaction	773490004
HypersensitivityReaction	773491000
HypersensitivityReaction	773496005
HypersensitivityReaction	773499003
HypersensitivityReaction	773500007
HypersensitivityReaction	773502004
HypersensitivityReaction	773505002
HypersensitivityReaction	773510003
HypersensitivityReaction	773515008
HypersensitivityReaction	773518005
HypersensitivityReaction	781684006
HypersensitivityReaction	782513000
HypersensitivityReaction	782520007
HypersensitivityReaction	782529008
HypersensitivityReaction	782530003
HypersensitivityReaction	782552007
HypersensitivityReaction	782554008

HypersensitivityReaction	782555009
HypersensitivityReaction	782558006
HypersensitivityReaction	782560008
HypersensitivityReaction	782561007
HypersensitivityReaction	782562000
HypersensitivityReaction	782563005
HypersensitivityReaction	782575000
HypersensitivityReaction	782580009
HypersensitivityReaction	782587007
HypersensitivityReaction	782588002
HypersensitivityReaction	782589005
HypersensitivityReaction	782590001
HypersensitivityReaction	782591002
HypersensitivityReaction	782592009
HypersensitivityReaction	782594005
HypersensitivityReaction	782595006
HypersensitivityReaction	782596007
HypersensitivityReaction	782597003
HypersensitivityReaction	782598008
HypersensitivityReaction	782599000
HypersensitivityReaction	782600002
HypersensitivityReaction	782607004
HypersensitivityReaction	788163005
HypersensitivityReaction	788164004
HypersensitivityReaction	788779003
HypersensitivityReaction	788781001
HypersensitivityReaction	788801007
HypersensitivityReaction	788802000
HypersensitivityReaction	788803005
HypersensitivityReaction	789251000
HypersensitivityReaction	829976001
HypersensitivityReaction	830080006
HypersensitivityReaction	830081005
HypersensitivityReaction	830165002
HypersensitivityReaction	830167005
HypersensitivityReaction	830168000
HypersensitivityReaction	830169008
HypersensitivityReaction	830170009
HypersensitivityReaction	830171008
HypersensitivityReaction	830172001
HypersensitivityReaction	830173006
HypersensitivityReaction	830174000
HypersensitivityReaction	830175004
HypersensitivityReaction	830176003

HypersensitivityReaction	830177007
HypersensitivityReaction	830178002
HypersensitivityReaction	830212002
HypersensitivityReaction	830259009
HypersensitivityReaction	830260004
HypersensitivityReaction	830277004
HypersensitivityReaction	838367000
HypersensitivityReaction	860604008
HypersensitivityReaction	860695004
HypersensitivityReaction	860764004
HypersensitivityReaction	860765003
HypersensitivityReaction	860773007
HypersensitivityReaction	860775000
HypersensitivityReaction	860776004
HypersensitivityReaction	860777008
HypersensitivityReaction	860778003
HypersensitivityReaction	860900002
HypersensitivityReaction	860901003
HypersensitivityReaction	860902005
HypersensitivityReaction	860903000
HypersensitivityReaction	860904006
HypersensitivityReaction	860907004
HypersensitivityReaction	860927003
HypersensitivityReaction	860931009
HypersensitivityReaction	860936004
HypersensitivityReaction	860956003
HypersensitivityReaction	863903001
HypersensitivityReaction	865878007
HypersensitivityReaction	870608005
HypersensitivityReaction	870712009
HypersensitivityReaction	870714005
HypersensitivityReaction	870727009
HypersensitivityReaction	870730002
HypersensitivityReaction	870731003
HypersensitivityReaction	870746005
HypersensitivityReaction	870747001
HypersensitivityReaction	870748006
HypersensitivityReaction	871500003
HypersensitivityReaction	871501004
HypersensitivityReaction	871503001
HypersensitivityReaction	871505008
HypersensitivityReaction	871506009
HypersensitivityReaction	871508005
HypersensitivityReaction	871509002

HypersensitivityReaction	871511006
HypersensitivityReaction	871554001
HypersensitivityReaction	871920005
HypersensitivityReaction	871926004
HypersensitivityReaction	871927008
HypersensitivityReaction	871930001
HypersensitivityReaction	871931002
HypersensitivityReaction	890231006
HypersensitivityReaction	1003755004
HypersensitivityReaction	1003757007
HypersensitivityReaction	1003758002
HypersensitivityReaction	1119209001
HypersensitivityReaction	1145003007
HypersensitivityReaction	11000119105
HypersensitivityReaction	441000119109
HypersensitivityReaction	601000119109
HypersensitivityReaction	5361000122104
HypersensitivityReaction	5611000122107
HypersensitivityReaction	6071000119100
HypersensitivityReaction	6671000122109
HypersensitivityReaction	11861000122107
HypersensitivityReaction	12921000122102
HypersensitivityReaction	13181000122107
HypersensitivityReaction	13221000122100
HypersensitivityReaction	13511000122108
HypersensitivityReaction	18321000122100
HypersensitivityReaction	18361000122109
HypersensitivityReaction	18401000122101
HypersensitivityReaction	21191000122102
HypersensitivityReaction	22481000122100
HypersensitivityReaction	23171000122102
HypersensitivityReaction	23181000122104
HypersensitivityReaction	48821000119104
HypersensitivityReaction	89171000000103
HypersensitivityReaction	103781000119103
HypersensitivityReaction	107561000000103
HypersensitivityReaction	107581000000107
HypersensitivityReaction	107591000000109
HypersensitivityReaction	107601000000103
HypersensitivityReaction	109991000119100
HypersensitivityReaction	129571000119102
HypersensitivityReaction	129581000119104
HypersensitivityReaction	139851000119105
HypersensitivityReaction	151201000119107

HypersensitivityReaction	164441000000106
HypersensitivityReaction	165991000000105
HypersensitivityReaction	171481000000107
HypersensitivityReaction	171491000000109
HypersensitivityReaction	171721000000102
HypersensitivityReaction	185971000000108
HypersensitivityReaction	185981000000105
HypersensitivityReaction	185991000000107
HypersensitivityReaction	186001000000106
HypersensitivityReaction	189411000000106
HypersensitivityReaction	189421000000100
HypersensitivityReaction	189431000000103
HypersensitivityReaction	189441000000107
HypersensitivityReaction	192851000000103
HypersensitivityReaction	192891000000106
HypersensitivityReaction	192901000000107
HypersensitivityReaction	192911000000109
HypersensitivityReaction	192921000000103
HypersensitivityReaction	192931000000101
HypersensitivityReaction	196481000000105
HypersensitivityReaction	201551000000109
HypersensitivityReaction	215661000000107
HypersensitivityReaction	215671000000100
HypersensitivityReaction	218731000000100
HypersensitivityReaction	218741000000109
HypersensitivityReaction	243351000000105
HypersensitivityReaction	243611000000104
HypersensitivityReaction	243621000000105
HypersensitivityReaction	243631000000107
HypersensitivityReaction	243641000000103
HypersensitivityReaction	243651000000100
HypersensitivityReaction	243661000000102
HypersensitivityReaction	311211000000101
HypersensitivityReaction	325361000000100
HypersensitivityReaction	325651000000108
HypersensitivityReaction	338301000000109
HypersensitivityReaction	338311000000106
HypersensitivityReaction	338451000000103
HypersensitivityReaction	339761000000102
HypersensitivityReaction	340681000000108
HypersensitivityReaction	344851000000108
HypersensitivityReaction	368911000000100
HypersensitivityReaction	369131000000104
HypersensitivityReaction	398841000000105

HypersensitivityReaction	407711000000108
HypersensitivityReaction	410901000000105
HypersensitivityReaction	413881000000102
HypersensitivityReaction	416991000000101
HypersensitivityReaction	417551000000100
HypersensitivityReaction	417751000000105
HypersensitivityReaction	417761000000108
HypersensitivityReaction	424251000000100
HypersensitivityReaction	427311000000103
HypersensitivityReaction	427321000000109
HypersensitivityReaction	427351000000104
HypersensitivityReaction	431491000000108
HypersensitivityReaction	441321000000103
HypersensitivityReaction	452311000000101
HypersensitivityReaction	465621000000104
HypersensitivityReaction	470141000000104
HypersensitivityReaction	470331000000106
HypersensitivityReaction	477001000000102
HypersensitivityReaction	479561000000104
HypersensitivityReaction	509081000000101
HypersensitivityReaction	509181000000102
HypersensitivityReaction	509191000000100
HypersensitivityReaction	509201000000103
HypersensitivityReaction	509441000000101
HypersensitivityReaction	509851000000101
HypersensitivityReaction	509871000000105
HypersensitivityReaction	509881000000107
HypersensitivityReaction	509891000000109
HypersensitivityReaction	509901000000105
HypersensitivityReaction	510241000000108
HypersensitivityReaction	510251000000106
HypersensitivityReaction	510261000000109
HypersensitivityReaction	510341000000101
HypersensitivityReaction	510621000000103
HypersensitivityReaction	510631000000101
HypersensitivityReaction	510641000000105
HypersensitivityReaction	522581000000107
HypersensitivityReaction	562151000000100
HypersensitivityReaction	579691000000102
HypersensitivityReaction	589231000000108
HypersensitivityReaction	601611000000106
HypersensitivityReaction	609161000000100
HypersensitivityReaction	644281000000107
HypersensitivityReaction	653841000000104

HypersensitivityReaction	680131000000102
HypersensitivityReaction	680451000000104
HypersensitivityReaction	707361000000103
HypersensitivityReaction	771231000000101
HypersensitivityReaction	776431000000108
HypersensitivityReaction	776441000000104
HypersensitivityReaction	778401000000101
HypersensitivityReaction	784551000000103
HypersensitivityReaction	803791000000102
HypersensitivityReaction	806161000000101
HypersensitivityReaction	806171000000108
HypersensitivityReaction	806181000000105
HypersensitivityReaction	806191000000107
HypersensitivityReaction	808301000000104
HypersensitivityReaction	831681000000104
HypersensitivityReaction	848481000000105
HypersensitivityReaction	848491000000107
HypersensitivityReaction	848501000000101
HypersensitivityReaction	848511000000104
HypersensitivityReaction	854161000000102
HypersensitivityReaction	855281000006112
HypersensitivityReaction	856161000000105
HypersensitivityReaction	863481000000106
HypersensitivityReaction	863491000000108
HypersensitivityReaction	885921000000102
HypersensitivityReaction	922181000000106
HypersensitivityReaction	922191000000108
HypersensitivityReaction	956271000000104
HypersensitivityReaction	956281000000102
HypersensitivityReaction	956291000000100
HypersensitivityReaction	956301000000101
HypersensitivityReaction	956311000000104
HypersensitivityReaction	956321000000105
HypersensitivityReaction	963681000000101
HypersensitivityReaction	963721000000108
HypersensitivityReaction	963731000000105
HypersensitivityReaction	963911000000100
HypersensitivityReaction	981221000000103
HypersensitivityReaction	981231000000101
HypersensitivityReaction	985251000000106
HypersensitivityReaction	985261000000109
HypersensitivityReaction	985271000000102
HypersensitivityReaction	985281000000100
HypersensitivityReaction	990561000000102

HypersensitivityReaction	1033621000000103
HypersensitivityReaction	1033631000000101
HypersensitivityReaction	1053591000000102
HypersensitivityReaction	1077861000000101
HypersensitivityReaction	1077871000000108
HypersensitivityReaction	1082641000119102
HypersensitivityReaction	1086701000000102
HypersensitivityReaction	1104821000000102
HypersensitivityReaction	1240341000000102
HypersensitivityReaction	1240351000000104
HypersensitivityReaction	1324711000000102
HypersensitivityReaction	1326121000000101
HypersensitivityReaction	1326141000000108
HypersensitivityReaction	1757211000006111
HypersensitivityReaction	1779631000006110
HypersensitivityReaction	1899821000006117
HypersensitivityReaction	1954031000006112
HypersensitivityReaction	2019561000006113
HypersensitivityReaction	10629471000119106
HypersensitivityReaction	10674991000119104
HypersensitivityReaction	10675431000119106
HypersensitivityReaction	10675471000119109
HypersensitivityReaction	10675871000119106
HypersensitivityReaction	10675911000119109
HypersensitivityReaction	10676391000119108
HypersensitivityReaction	10676431000119103
HypersensitivityReaction	10692681000119108
HypersensitivityReaction	15911801000119104
HypersensitivityReaction	15919441000119101
HypersensitivityReaction	15919781000119104
HypersensitivityReaction	15919821000119109
HypersensitivityReaction	15920041000119105
HypersensitivityReaction	15920121000119103
HypersensitivityReaction	15920161000119108
HypersensitivityReaction	15920201000119103
HypersensitivityReaction	15920361000119106
HypersensitivityReaction	15920401000119102
HypersensitivityReaction	15920481000119105
HypersensitivityReaction	15920521000119105
HypersensitivityReaction	15920601000119100
HypersensitivityReaction	16067171000119102
HypersensitivityReaction	16067291000119109
HypersensitivityReaction	16224471000119103
HypersensitivityReaction	16224591000119103

Influenza-likeIllness-WRpt	6142004
Influenza-likeIllness-WRpt	24662006
Influenza-likeIllness-WRpt	41269000
Influenza-likeIllness-WRpt	42964004
Influenza-likeIllness-WRpt	43692000
Influenza-likeIllness-WRpt	46171006
Influenza-likeIllness-WRpt	55604004
Influenza-likeIllness-WRpt	61700007
Influenza-likeIllness-WRpt	63039003
Influenza-likeIllness-WRpt	74644004
Influenza-likeIllness-WRpt	78046005
Influenza-likeIllness-WRpt	78431007
Influenza-likeIllness-WRpt	81524006
Influenza-likeIllness-WRpt	84037004
Influenza-likeIllness-WRpt	95891005
Influenza-likeIllness-WRpt	139168000
Influenza-likeIllness-WRpt	161913008
Influenza-likeIllness-WRpt	194946005
Influenza-likeIllness-WRpt	195878008
Influenza-likeIllness-WRpt	195920000
Influenza-likeIllness-WRpt	195923003
Influenza-likeIllness-WRpt	195924009
Influenza-likeIllness-WRpt	195929004
Influenza-likeIllness-WRpt	309789002
Influenza-likeIllness-WRpt	309806000
Influenza-likeIllness-WRpt	313251006
Influenza-likeIllness-WRpt	315642008
Influenza-likeIllness-WRpt	408687004
Influenza-likeIllness-WRpt	427873006
Influenza-likeIllness-WRpt	442438000
Influenza-likeIllness-WRpt	442696006
Influenza-likeIllness-WRpt	450715004
Influenza-likeIllness-WRpt	450716003
Influenza-likeIllness-WRpt	707448003
Influenza-likeIllness-WRpt	711128004
Influenza-likeIllness-WRpt	713083002
Influenza-likeIllness-WRpt	719590007
Influenza-likeIllness-WRpt	719865001
Influenza-likeIllness-WRpt	772810003
Influenza-likeIllness-WRpt	772828001
Influenza-likeIllness-WRpt	772839003
Influenza-likeIllness-WRpt	16311000119108
Influenza-likeIllness-WRpt	142921000119103
Influenza-likeIllness-WRpt	142931000119100

Influenza-likeIllness-WRpt	142941000119109
Influenza-likeIllness-WRpt	142951000119106
Influenza-likeIllness-WRpt	142961000119108
Influenza-likeIllness-WRpt	142981000119104
Influenza-likeIllness-WRpt	142991000119101
Influenza-likeIllness-WRpt	143111000119103
Influenza-likeIllness-WRpt	280331000000102
Influenza-likeIllness-WRpt	292631000000106
Influenza-likeIllness-WRpt	328531000119104
Influenza-likeIllness-WRpt	418181000000104
Influenza-likeIllness-WRpt	418191000000102
Influenza-likeIllness-WRpt	430891000000103
Influenza-likeIllness-WRpt	441131000000104
Influenza-likeIllness-WRpt	505131000000104
Influenza-likeIllness-WRpt	510671000000104
Influenza-likeIllness-WRpt	540141000000105
Influenza-likeIllness-WRpt	540151000000108
Influenza-likeIllness-WRpt	616161000000107
Influenza-likeIllness-WRpt	677811000000106
Influenza-likeIllness-WRpt	856211000006111
Influenza-likeIllness-WRpt	1033051000000101
Influenza-likeIllness-WRpt	1033061000000103
Influenza-likeIllness-WRpt	1033071000000105
Influenza-likeIllness-WRpt	1033081000000107
Influenza-likeIllness-WRpt	1033091000000109
Influenza-likeIllness-WRpt	1033101000000101
Influenza-likeIllness-WRpt	1033111000000104
Influenza-likeIllness-WRpt	1033121000000105
Influenza-likeIllness-WRpt	1050601000000101
Influenza-likeIllness-WRpt	1050981000000100
Influenza-likeIllness-WRpt	1787121000006116
Influenza-likeIllness-WRpt	1787131000006118
Influenza-likeIllness-WRpt	10628871000119101
Influenza-likeIllness-WRpt	10628911000119103
Influenza-likeIllness-WRpt	10629191000119100
Influenza-likeIllness-WRpt	10629351000119108
Influenza-likeIllness-WRpt	10685111000119102
InjectionSiteBruising	95401009
InjectionSiteBurning	95389008
InjectionSiteErythema	213338000
InjectionSiteInduration	95402002
InjectionSiteInduration	213337005
InjectionSiteInflammation	95391000
InjectionSiteIrritated	95379009

InjectionSitePain	95388000
InjectionSitePain	213339008
InjectionSitePruritus	863894003
InjectionSiteRash	860897009
InjectionSiteSwelling	213340005
InjectionSiteUrticaria	95394008
Insomnia	3277001
Insomnia	3972004
Insomnia	24121004
Insomnia	38376001
Insomnia	41975002
Insomnia	45291004
Insomnia	53758003
Insomnia	54230003
Insomnia	59050008
Insomnia	67062000
Insomnia	67233009
Insomnia	81608000
Insomnia	83157008
Insomnia	88982005
Insomnia	162204000
Insomnia	191997003
Insomnia	192454004
Insomnia	193462001
Insomnia	198437004
Insomnia	206748000
Insomnia	206749008
Insomnia	248259004
Insomnia	268652009
Insomnia	272025006
Insomnia	274233003
Insomnia	425832009
Insomnia	426257002
Insomnia	472819006
Insomnia	724748004
Insomnia	762348004
Insomnia	27971000119105
Insomnia	90361000119105
Insomnia	91411000119105
Insomnia	93681000119102
Insomnia	135311000119100
Insomnia	288081000119106
Insomnia	288091000119109
Insomnia	288101000119104

Insomnia	477671000000104
Insomnia	495661000000105
Insomnia	502171000000108
Insomnia	671491000000108
Lethargy	52663000
Lethargy	158171005
Lethargy	161871003
Lethargy	206769003
Lethargy	214264003
Lethargy	248275001
Lethargy	271797003
Lethargy	495781000000109
Lymphadenopathy	3502005
Lymphadenopathy	6136003
Lymphadenopathy	10893003
Lymphadenopathy	13272007
Lymphadenopathy	15170009
Lymphadenopathy	16409009
Lymphadenopathy	19471005
Lymphadenopathy	20197001
Lymphadenopathy	23794001
Lymphadenopathy	30746006
Lymphadenopathy	32035007
Lymphadenopathy	34287003
Lymphadenopathy	36585007
Lymphadenopathy	41174002
Lymphadenopathy	43766001
Lymphadenopathy	44897000
Lymphadenopathy	46958009
Lymphadenopathy	47238006
Lymphadenopathy	48573006
Lymphadenopathy	49498000
Lymphadenopathy	49631001
Lymphadenopathy	52324001
Lymphadenopathy	54084005
Lymphadenopathy	64757003
Lymphadenopathy	74387008
Lymphadenopathy	75053002
Lymphadenopathy	77038006
Lymphadenopathy	78436002
Lymphadenopathy	82495004
Lymphadenopathy	85692003
Lymphadenopathy	87117006
Lymphadenopathy	87500009

Lymphadenopathy	92152005
Lymphadenopathy	92215004
Lymphadenopathy	92324006
Lymphadenopathy	93182006
Lymphadenopathy	94347008
Lymphadenopathy	94686001
Lymphadenopathy	95892003
Lymphadenopathy	111002006
Lymphadenopathy	125574005
Lymphadenopathy	127074004
Lymphadenopathy	127075003
Lymphadenopathy	127076002
Lymphadenopathy	127077006
Lymphadenopathy	127078001
Lymphadenopathy	127079009
Lymphadenopathy	127080007
Lymphadenopathy	127081006
Lymphadenopathy	127082004
Lymphadenopathy	127083009
Lymphadenopathy	127084003
Lymphadenopathy	127085002
Lymphadenopathy	127086001
Lymphadenopathy	127087005
Lymphadenopathy	127088000
Lymphadenopathy	127089008
Lymphadenopathy	127090004
Lymphadenopathy	127091000
Lymphadenopathy	127092007
Lymphadenopathy	127093002
Lymphadenopathy	127094008
Lymphadenopathy	127095009
Lymphadenopathy	127096005
Lymphadenopathy	127097001
Lymphadenopathy	127098006
Lymphadenopathy	127099003
Lymphadenopathy	127100006
Lymphadenopathy	127101005
Lymphadenopathy	127102003
Lymphadenopathy	127103008
Lymphadenopathy	127104002
Lymphadenopathy	127105001
Lymphadenopathy	127106000
Lymphadenopathy	127107009
Lymphadenopathy	127108004

Lymphadenopathy	127109007
Lymphadenopathy	127110002
Lymphadenopathy	127111003
Lymphadenopathy	127112005
Lymphadenopathy	127113000
Lymphadenopathy	127114006
Lymphadenopathy	127116008
Lymphadenopathy	127117004
Lymphadenopathy	127118009
Lymphadenopathy	127119001
Lymphadenopathy	127120007
Lymphadenopathy	127121006
Lymphadenopathy	127122004
Lymphadenopathy	127123009
Lymphadenopathy	127124003
Lymphadenopathy	127125002
Lymphadenopathy	127126001
Lymphadenopathy	127127005
Lymphadenopathy	127128000
Lymphadenopathy	127129008
Lymphadenopathy	127130003
Lymphadenopathy	127131004
Lymphadenopathy	127132006
Lymphadenopathy	127133001
Lymphadenopathy	127134007
Lymphadenopathy	127135008
Lymphadenopathy	127136009
Lymphadenopathy	127137000
Lymphadenopathy	127138005
Lymphadenopathy	127139002
Lymphadenopathy	127140000
Lymphadenopathy	127141001
Lymphadenopathy	127142008
Lymphadenopathy	127143003
Lymphadenopathy	127144009
Lymphadenopathy	127145005
Lymphadenopathy	127146006
Lymphadenopathy	127147002
Lymphadenopathy	127148007
Lymphadenopathy	127149004
Lymphadenopathy	127150004
Lymphadenopathy	127151000
Lymphadenopathy	127152007
Lymphadenopathy	127153002

Lymphadenopathy	127154008
Lymphadenopathy	127155009
Lymphadenopathy	127156005
Lymphadenopathy	127157001
Lymphadenopathy	127158006
Lymphadenopathy	127159003
Lymphadenopathy	127160008
Lymphadenopathy	127161007
Lymphadenopathy	127162000
Lymphadenopathy	127163005
Lymphadenopathy	127164004
Lymphadenopathy	127166002
Lymphadenopathy	127167006
Lymphadenopathy	127168001
Lymphadenopathy	127169009
Lymphadenopathy	127170005
Lymphadenopathy	127171009
Lymphadenopathy	127172002
Lymphadenopathy	127173007
Lymphadenopathy	127174001
Lymphadenopathy	127175000
Lymphadenopathy	127176004
Lymphadenopathy	127177008
Lymphadenopathy	127178003
Lymphadenopathy	127179006
Lymphadenopathy	127180009
Lymphadenopathy	127181008
Lymphadenopathy	127182001
Lymphadenopathy	127183006
Lymphadenopathy	127184000
Lymphadenopathy	127185004
Lymphadenopathy	127186003
Lymphadenopathy	127187007
Lymphadenopathy	127188002
Lymphadenopathy	127189005
Lymphadenopathy	127190001
Lymphadenopathy	127191002
Lymphadenopathy	127192009
Lymphadenopathy	127193004
Lymphadenopathy	127194005
Lymphadenopathy	127195006
Lymphadenopathy	127196007
Lymphadenopathy	127197003
Lymphadenopathy	127198008

Lymphadenopathy	127199000
Lymphadenopathy	127200002
Lymphadenopathy	127201003
Lymphadenopathy	127202005
Lymphadenopathy	127203000
Lymphadenopathy	127204006
Lymphadenopathy	127205007
Lymphadenopathy	127206008
Lymphadenopathy	127207004
Lymphadenopathy	127208009
Lymphadenopathy	127209001
Lymphadenopathy	127210006
Lymphadenopathy	127211005
Lymphadenopathy	127212003
Lymphadenopathy	127213008
Lymphadenopathy	127214002
Lymphadenopathy	127217009
Lymphadenopathy	127250009
Lymphadenopathy	127251008
Lymphadenopathy	127252001
Lymphadenopathy	127253006
Lymphadenopathy	127268007
Lymphadenopathy	127270003
Lymphadenopathy	127273001
Lymphadenopathy	164145000
Lymphadenopathy	164147008
Lymphadenopathy	164149006
Lymphadenopathy	164153008
Lymphadenopathy	181225007
Lymphadenopathy	186198005
Lymphadenopathy	186259007
Lymphadenopathy	186326007
Lymphadenopathy	191375005
Lymphadenopathy	191377002
Lymphadenopathy	200697006
Lymphadenopathy	200698001
Lymphadenopathy	200699009
Lymphadenopathy	207033006
Lymphadenopathy	207034000
Lymphadenopathy	207035004
Lymphadenopathy	207036003
Lymphadenopathy	207037007
Lymphadenopathy	207038002
Lymphadenopathy	207039005

Lymphadenopathy	233743002
Lymphadenopathy	233744008
Lymphadenopathy	234090004
Lymphadenopathy	234091000
Lymphadenopathy	234092007
Lymphadenopathy	234094008
Lymphadenopathy	238405002
Lymphadenopathy	238676008
Lymphadenopathy	238809002
Lymphadenopathy	240414006
Lymphadenopathy	264508001
Lymphadenopathy	267548000
Lymphadenopathy	274740001
Lymphadenopathy	274741002
Lymphadenopathy	274744005
Lymphadenopathy	281898005
Lymphadenopathy	300929003
Lymphadenopathy	302061009
Lymphadenopathy	328488009
Lymphadenopathy	329489009
Lymphadenopathy	394677008
Lymphadenopathy	399945005
Lymphadenopathy	400131007
Lymphadenopathy	403962006
Lymphadenopathy	409648008
Lymphadenopathy	421977001
Lymphadenopathy	425061006
Lymphadenopathy	426121003
Lymphadenopathy	426874006
Lymphadenopathy	445718006
Lymphadenopathy	446292002
Lymphadenopathy	447803000
Lymphadenopathy	704281009
Lymphadenopathy	707149004
Lymphadenopathy	713507008
Lymphadenopathy	714460000
Lymphadenopathy	870348009
Lymphadenopathy	49991000119104
Lymphadenopathy	239701000000105
Lymphadenopathy	415111000000104
Lymphadenopathy	425661000000104
Lymphadenopathy	463481000000101
Lymphadenopathy	497821000000106
Lymphadenopathy	497831000000108

Lymphadenopathy	497841000000104
Lymphadenopathy	497851000000101
Lymphadenopathy	497861000000103
Lymphadenopathy	538021000000100
Lymphadenopathy	541001000000104
Lymphadenopathy	541111000000101
Lymphadenopathy	541121000000107
Lymphadenopathy	541131000000109
Lymphadenopathy	564571000000108
Lymphadenopathy	571381000000105
Lymphadenopathy	583771000000101
Lymphadenopathy	587001000000103
Lymphadenopathy	588971000000106
Lymphadenopathy	597831000000100
Lymphadenopathy	725531000000101
Lymphadenopathy	757021000000101
Lymphadenopathy	1090191000000107
Lymphadenopathy	10629071000119102
Lymphadenopathy	10629111000119109
Myocarditis	279001
Myocarditis	488007
Myocarditis	756003
Myocarditis	2024009
Myocarditis	4082005
Myocarditis	8402005
Myocarditis	8676001
Myocarditis	8916007
Myocarditis	11176009
Myocarditis	13452004
Myocarditis	18484008
Myocarditis	22653005
Myocarditis	26117009
Myocarditis	28880005
Myocarditis	30328003
Myocarditis	30496006
Myocarditis	31993003
Myocarditis	34336004
Myocarditis	37217002
Myocarditis	37925008
Myocarditis	40532006
Myocarditis	44630002
Myocarditis	45093008
Myocarditis	46701001
Myocarditis	47292005

Myocarditis	50920009
Myocarditis	54830008
Myocarditis	55087008
Myocarditis	55482007
Myocarditis	60812006
Myocarditis	61012002
Myocarditis	63462008
Myocarditis	64043005
Myocarditis	64190005
Myocarditis	65718001
Myocarditis	69589006
Myocarditis	72527006
Myocarditis	74918002
Myocarditis	76534005
Myocarditis	78046005
Myocarditis	79096004
Myocarditis	88782004
Myocarditis	89141000
Myocarditis	91025000
Myocarditis	91468009
Myocarditis	187195003
Myocarditis	194709000
Myocarditis	194750008
Myocarditis	194942007
Myocarditis	194943002
Myocarditis	194944008
Myocarditis	194945009
Myocarditis	194946005
Myocarditis	194947001
Myocarditis	194948006
Myocarditis	194949003
Myocarditis	194950003
Myocarditis	194956009
Myocarditis	194957000
Myocarditis	194958005
Myocarditis	195033009
Myocarditis	195136004
Myocarditis	195569005
Myocarditis	233867000
Myocarditis	233868005
Myocarditis	233869002
Myocarditis	251060004
Myocarditis	266238009
Myocarditis	275512002

Myocarditis	413933004
Myocarditis	421272004
Myocarditis	421929001
Myocarditis	427372006
Myocarditis	427443009
Myocarditis	460317007
Myocarditis	460329003
Myocarditis	460338001
Myocarditis	460345001
Myocarditis	460352004
Myocarditis	460620006
Myocarditis	471841009
Myocarditis	472707009
Myocarditis	472811009
Myocarditis	703274008
Myocarditis	713318009
Myocarditis	723863003
Myocarditis	723865005
Myocarditis	870301007
Myocarditis	871587002
Myocarditis	871640001
Myocarditis	880012008
Myocarditis	1052287002
Myocarditis	142951000119106
Myocarditis	142981000119104
Myocarditis	402301000000105
Myocarditis	412881000000106
Myocarditis	419381000000107
Myocarditis	443881000000106
Myocarditis	466071000000102
Myocarditis	665471000000100
Myocarditis	1089931000119105
Myocarditis	1240531000000103
Myocarditis	10629351000119108
Pericarditis	2554006
Pericarditis	3238004
Pericarditis	3589003
Pericarditis	5836005
Pericarditis	11176009
Pericarditis	14455008
Pericarditis	15016005
Pericarditis	15555002
Pericarditis	15676002
Pericarditis	17079009

Pericarditis	17668000
Pericarditis	23842007
Pericarditis	27806003
Pericarditis	40959008
Pericarditis	41739008
Pericarditis	44595001
Pericarditis	48228003
Pericarditis	50902000
Pericarditis	53874003
Pericarditis	55043004
Pericarditis	61558009
Pericarditis	65447009
Pericarditis	66704002
Pericarditis	67256000
Pericarditis	70189005
Pericarditis	81580000
Pericarditis	86504008
Pericarditis	90428001
Pericarditis	105982005
Pericarditis	173574009
Pericarditis	174511002
Pericarditis	176401009
Pericarditis	177291008
Pericarditis	194903007
Pericarditis	194905000
Pericarditis	194906004
Pericarditis	194907008
Pericarditis	194908003
Pericarditis	194909006
Pericarditis	194910001
Pericarditis	194911002
Pericarditis	194916007
Pericarditis	194917003
Pericarditis	194918008
Pericarditis	195556006
Pericarditis	233881003
Pericarditis	233883000
Pericarditis	233884006
Pericarditis	233886008
Pericarditis	266235007
Pericarditis	275513007
Pericarditis	459184006
Pericarditis	460439008
Pericarditis	460440005

Pericarditis	460445000
Pericarditis	460450006
Pericarditis	460457009
Pericarditis	460464006
Pericarditis	703384007
Pericarditis	723088005
Pericarditis	34791000119103
Pericarditis	402071000000104
Pericarditis	419151000000103
Pericarditis	455351000000108
Pericarditis	455371000000104
Pericarditis	608501000000102
Pericarditis	624891000000105
Pericarditis	654251000000104
Pericarditis	682791000000101
SneezingSymptom	76067001
SneezingSymptom	119251002
SneezingSymptom	162367006
SneezingSymptom	206999004
SneezingSymptom	497551000000104
SneezingSymptom	564161000000106

Supplementary Table S2. Model 2 results with age centred at 30.

**Model 2**	BNT162b2	ChAdOx1	mRNA-1273
D1: -7 to -1	1.00 (0.98-1.03)	0.99 (0.96-1.02)	0.96 (0.86-1.07)
D1: 0 to 7	0.98 (0.95-1.00)	1.00 (0.97-1.03)	1.12 (1.02-1.23)*
D2: -7 to -1	0.95 (0.92-0.98)**	0.98 (0.95-1.01)	0.95 (0.83-1.09)
D2: 0 to 7	1.03 (1.00-1.06)*	1.01 (0.98-1.04)	1.15 (1.03-1.30)*
D1: -7 to -1 x age	0.9978 (0.9970-0.9987)***	0.9988 (0.9978-0.9999)*	1.0094 (0.9990-1.0199)
D1: 0 to 7 x age	0.9973 (0.9965-0.9982)***	0.9982 (0.9973-0.9992)***	1.0035 (0.9944-1.0128)
D2: -7 to -1 x age	0.9981 (0.9972-0.9991)***	0.9993 (0.9982-1.0004)	1.0062 (0.9932-1.0193)
D2: 0 to 7 x age	0.9965 (0.9956-0.9973)***	0.9979 (0.9968-0.9989)***	0.9988 (0.9874-1.0103)

Supplementary Table S3. Model 2 results with age centred at 70 years.

**Model 2**	BNT162b2	ChAdOx1	mRNA-1273
D1: -7 to -1	0.92 (0.90-0.95)***	0.94 (0.92-0.97)***	1.39 (0.94-2.07)
D1: 0 to 7	0.88 (0.86-0.90)***	0.93 (0.91-0.95)***	1.29 (0.91-1.84)
D2: -7 to -1	0.88 (0.86-0.90)***	0.95 (0.93-0.97)***	1.22 (0.76-1.95)
D2: 0 to 7	0.89 (0.87-0.92)***	0.92 (0.90-0.95)***	1.10 (0.72-1.68)
D1: -7 to -1 x age	0.9978 (0.9970-0.9987)***	0.9988 (0.9978-0.9999)*	1.0094 (0.9990-1.0199)
D1: 0 to 7 x age	0.9973 (0.9965-0.9982)***	0.9982 (0.9973-0.9992)***	1.0035 (0.9944-1.0128)
D2: -7 to -1 x age	0.9981 (0.9972-0.9991)***	0.9993 (0.9982-1.0004)	1.0062 (0.9932-1.0193)
D2: 0 to 7 x age	0.9965 (0.9956-0.9973)***	0.9979 (0.9968-0.9989)***	0.9988 (0.9874-1.0103)

Supplementary Table S4. Model 2 results with the addition of risk periods of 8-14 days and 15-21 days after vaccination, relative incidence (95% confidence interval)

**Model 2**	BNT162b2	ChAdOx1	mRNA-1273
D1: -7 to -1	0.96 (0.94-0.98)***	0.97 (0.95-0.98)***	1.18 (0.96-1.44)
D1: 0 to 7	0.92 (0.91-0.94)***	0.97 (0.95-0.98)***	1.23 (1.03-1.47)*
D1: 8 to 14	0.99 (0.97-1.01)	1.00 (0.98-1.02)	1.55 (1.30-1.85)***
D1: 15 to 21	0.99 (0.97-1.01)	1.00 (0.98-1.02)	0.94 (0.76-1.17)
D2: -7 to -1	0.92 (0.90-0.94)***	0.97 (0.95-0.99)**	1.09 (0.87-1.38)
D2: 0 to 7	0.96 (0.94-0.98)***	0.97 (0.95-0.99)***	1.14 (0.92-1.41)
D2: 8 to 14	1.00 (0.98-1.02)	1.03 (1.02-1.05)**	1.06 (0.83-1.35)
D2: 15 to 21	1.00 (0.97-1.02)	1.02 (1.00-1.04)*	0.94 (0.72-1.22)
D1: -7 to -1 x age	0.9977 (0.9969-0.9986)***	0.9988 (0.9977-0.9998)*	1.0097 (0.9993-1.0202)
D1: 0 to 7 x age	0.9972 (0.9964-0.9980)***	0.9981 (0.9972-0.9991)***	1.0040 (0.9948-1.0132)
D1: 8 to 14 x age	0.9984 (0.9976-0.9993)***	0.9987 (0.9976-0.9997)*	1.0151 (1.0056-1.0247)**
D1: 15 to 21 x age	0.9990 (0.9981-0.9999)*	0.9991 (0.9980-1.0001)	0.9973 (0.9866-1.0081)
D2: -7 to -1 x age	0.9980 (0.9971-0.9990)***	0.9992 (0.9981-1.0003)	1.0072 (0.9941-1.0203)
D2: 0 to 7 x age	0.9964 (0.9955-0.9973)***	0.9978 (0.9968-0.9988)***	0.9996 (0.9882-1.0112)
D2: 8 to 14 x age	0.9995 (0.9986-1.0004)	0.9993 (0.9982-1.0003)	1.0055 (0.9916-1.0196)
D2: 15 to 21 x age	1.0010 (1.0000-1.0019)*	1.0008 (0.9997-1.0019)	0.9983 (0.9831-1.0137)

Note: * p<0.05, ** p<0.01, *** p<0.001
Age centred at 50 years.
